# New principle of busbar protection based on a fundamental frequency polarity comparison

**DOI:** 10.1371/journal.pone.0213308

**Published:** 2019-03-21

**Authors:** Hao Wu, Xingxing Dong, Qiaomei Wang

**Affiliations:** Automation and Information Engineering, Sichuan University of Science and Engineering, Zigong, China; Newcastle University, UNITED KINGDOM

## Abstract

To overcome the contradiction between speed and reliability in existing busbar protection schemes, a new busbar protection algorithm based on a polarity comparison of fundamental frequency currents is proposed. The algorithm extracts the fundamental frequency components of the fault reference current and the virtual current through a wavelet transform. The angle between the two currents is used to characterize the polarity relationship. The polarities of the virtual current and the reference current are the same when an internal fault occurs, and the angle will be small. The polarities of the two currents are opposite for an external fault, in which case the angle is larger. By analysing the variation characteristics of the angle between faults inside and outside busbar, a protection criterion is established, and the fault area is determined. In simulation results based on PSCAD/EMTDC, the algorithm can quickly and reliably identify the faults inside and outside the busbar area, and its performance is not affected by the initial fault angle, fault resistance, fault type or capacitor voltage transformer (CVT) transmission characteristics.

## Introduction

Accurate identification of the fault area after a busbar fault will help quickly remove the fault and improve the stability of the power system [1]. There are two kinds of busbar protection: fundamental frequency protection and transient protection [2]. Busbar protection at the fundamental frequency is mainly used to distinguish the fault region by the combination of a polarity comparison and an amplitude comparison. The polarity relationship is mainly expressed by comparing the phase relation between the voltage of the fundamental frequency fault component and the current and correlation degree of the current sampling value of each connection branch of the busbar [3,4]. In [5], fault discrimination was realized by analysing the ratio of the voltage phasor and the sum of the current phase of the busbar branch. The ratio for an external fault is large, and the phase angle is close to 90. The ratio for an internal fault is small, and the phase angle is close to 0. Accordingly, the fault area of the busbar can be distinguished. In [6], the degree of correlation between the current sampling value of each branch of a busbar was calculated, and a criterion for busbar protection was constructed by using the waveform correlation of the fault in the busbar area and the saturation of the current transformer (CT). However, to accurately obtain the fundamental frequency phase, a strict filtering measure is needed. Filtering delay greatly reduces the speed of the protection action and does not meet the ultra-high-speed requirements of a smart grid. Due to the lack of anti-CT saturation in traditional power frequency busbar protection, in [7], a digital differential busbar protection scheme based on the generalized *α*-plane method was proposed. The algorithm mapped a periodic CT secondary current signal to the plane for fault zone identification, which could effectively address the insufficient anti-CT saturation ability of a traditional differential busbar. However, for reliable protection, a filtering process was added, and the protection response took a long time. In [8], a busbar differential protection principle with adaptive characteristics was proposed, which used the principles of alienation protection and differential protection to achieve better adaptive characteristics. Although this approach led to better relay performance than traditional differential protection, the time for fault diagnosis when CT saturation occurs was longer. Although the above reference solved the problem of an insufficient anti-CT saturation capability in traditional power frequency protection, the reaction speed of the protection was slightly slow in a super/extra-high voltage power grid.

Busbar protection based on the travelling wave principle has become a research hotspot because the response is rapid and not susceptible to CT saturation and distributed capacitor current. Busbar protection based on a transient polarity comparison mainly analyses the fault zone by analysing the polarity of the current travelling wave [9]. In [10], a busbar protection scheme based on wave impedance was proposed. The busbar fault zone was detected by analysing the polarity and magnitude of the current travelling wave of each branch of the busbar. [11] proposed a busbar protection principle based on a wavelet transform and a travelling wave polarity comparison. The method used the wavelet transform to extract the polarity of the initial travelling wave current and distinguished internal and external faults by comparing the current polarity of each branch of the busbar. In [12], a busbar fault zone identification scheme based on a superposed current polarity comparison was proposed. However, filtering the transient high-frequency signal of the fault requires strict filtering measures. The filtering delay reduces the protection speed and does not satisfy the requirements of the grid pair. Additionally, it slows the response of protection and does not meet the requirements of the grid for ultra-high-speed protection. In [13], when a fault occurred in the busbar zone, the wave impedance of each loop was equal, and the polarity was negative. When a fault occurred outside the protection zone, the impedances of the faulted line and non-faulted line were opposite to each other. Thus, internal and external faults of the busbar zone could be detected. However, only the travelling wave front information was utilized, leading to a lack of reliability. When the fault was outside the busbar zone, the impedances of the faulted and non-faulted lines were opposite to each other, allowing determination of the fault in the busbar area. However, only the traveling wave head information was utilized, and the criterion reliability was insufficient. [14] used a support vector machine (SVM) and an S transform to identify the fault zone, achieving highly accurate fault classification with diverse system parameters. In [15], a busbar protection scheme based on a relevance vector machine (RVM) was proposed. Based on a traditional SVM, the related parameters and kernel functions in the calculation were reduced. However, probabilistic predictions were not feasible because of the abrupt behaviour of the kernel functions.

Compared with fundamental frequency directional protection, traditional travelling wave protection mainly has two problems [16]. On the one hand, it is limited by a defect in travelling wave protection, i.e., failure when the voltage is zero. On the other hand, the capacitor voltage transformer (CVT) widely used in a high-voltage (HV) transmission system is greatly influenced by the transient process, and it cannot effectively transfer the high-frequency voltage signal [17–19].

To overcome the shortcomings of traditional travelling wave protection, a new principle of busbar protection based on a polarity comparison of the fundamental frequency components is proposed in this paper. The algorithm first defines the current as the reference current of an associated branch of the busbar, defines the sum of the other related branch currents as the virtual current, and uses the polarity relationship between the reference current and virtual current after the fault to determine the fault region. The algorithm uses the wavelet transform to decompose the reference and virtual currents into different frequency bands and obtain the corresponding reconstruction coefficient in the reconstruction of the frequency band of the fundamental frequency component. Obtaining the angle between the reconfiguration coefficient of the reference current and the reconstruction coefficient of the virtual current then allows characterization of the polarity relationship between the two and does not need additional filtering processing and a phase quantity. The calculation greatly improves the protection speed. At the same time, the fault current is only taken into account to avoid the influence of transient characteristics of the CVT on the rapidity and reliability of the protection elements. The simulation results based on PSCAD show that the proposed protection algorithm is simple, reliable and sensitive, and the performance is not affected by the initial angle of the fault, the fault resistance, the type of fault and other factors.

The remainder of this article is arranged as follows: The second section introduces the principle of current polarity comparison for busbar protection. The third section establishes a protection criterion according to the protection principle. The fourth section introduces the implementation process of the protection algorithm. The fifth section presents the fault simulation analysis. Finally, a summary is given.

## Principle of current polarity comparison for busbar protection

### Problems of the traditional travelling wave polarity comparator in busbar protection

For a traditional travelling wave polarity comparison in busbar protection, the results in the literature [20] show that when the fault occurs outside the busbar area, the initial travelling wave of the fault line voltage has opposite polarity to the initial travelling wave of the busbar, and the initial travelling wave of the non-faulted line voltage is the same as the initial travelling wave of the current. In the case of a fault in the area, the initial travelling wave and the initial wave polarity of the busbar line voltage are the same as in [20]. Table 1 analyses the fault characteristics of the voltage and current travelling wave detected on the line. All of these circuits consist of a voltage initial travelling wave and a current initial travelling wave Δ*S* = Δ*u*×Δ*i*.

**Table 1:** Characteristics of the electrical polarities of the travelling wave when there is a fault within the busbar zone or external to the zone.

Fault location	Electrical polarity of the travelling wave of the busbar Δ*u*	Electrical polarity of the travelling wave of the transmission line Δ*i*		Characteristic of the fault	
		Faulted transmission line	Non-faulted transmission line	Faulted transmission lineΔ*S*	Non-faulted transmission line Δ*S*
**Busbar fault**	Positive (+)		(+)		Δ*S* is greater than 0
	Negative (−)		(−)		Δ*S* is greater than 0
**Fault occurred on the transmission line**	Positive (+)	(−)	(+)	Δ*S* is less than 0	Δ*S* is greater than 0
	Negative (−)	(+)	(−)	Δ*S* is less than 0	Δ*S* is greater than 0

Table 1 shows the following:

1. When the busbar malfunction is external, for the faulted lines:
  Δu×Δi<0 (1)
  For the non-faulted lines:
  Δu×Δi>0 (2)
2. For an internal fault of the busbar,
  Δu×Δi>0 (3)

Although the fault region can be quickly judged by comparing the initial travelling wave and the polarity of the initial current, the traditional travelling wave polarity discrimination method has the following problems in a practical application:

1. Busbar protection based on a polarity-discriminated fault area needs a high-frequency transient voltage, while the widely used CVT is not good for the transmission of a high-frequency transient voltage. Thus, the performance of the travelling wave polarity comparison for busbar protection is greatly influenced by the CVT.
2. When the initial angle of the voltage fault is small, the amplitude of the fault voltage is small. At this time, the sensitivity of the busbar protection based on the comparison of the travelling wave polarity is very low, which will affect the judgement of the fault area.

To solve the above problems, a new principle of busbar protection based on a comparison of the reference current and the virtual current polarity is proposed to improve the reliability and sensitivity of the protection.

### New current polarity comparison busbar protection principle

**Fig 1:**
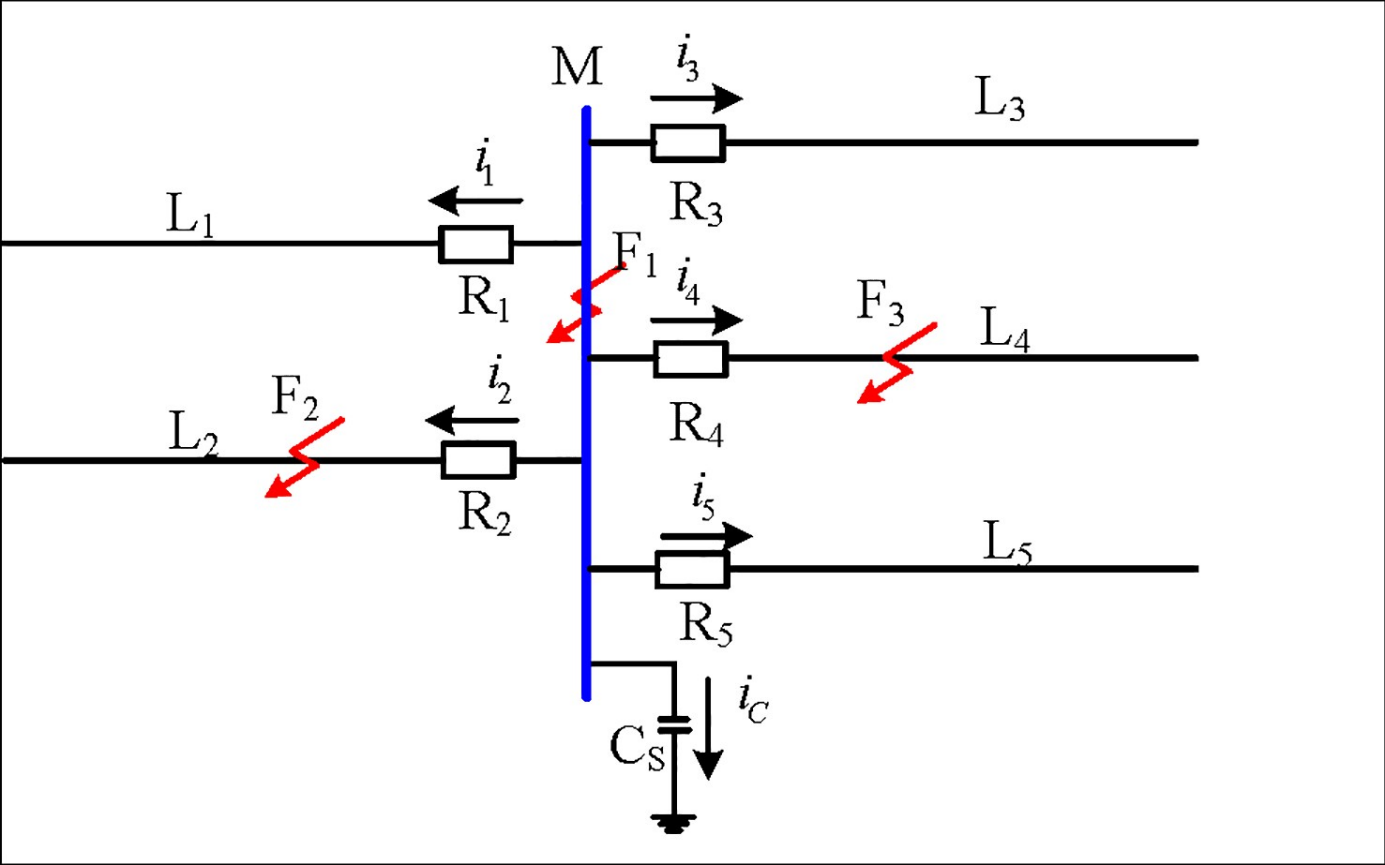
500kV busbar system.

Fig 1 shows a 500 kV substation busbar, in which L_1_-L_5_ are five lines connected by busbar M, and R_1_-R_5_ is the travelling wave protection unit installed near busbar M. The equivalent impedance of the busbar M to the ground stray capacitance is C_S_.

#### Analysis of the fault characteristics of a busbar internal fault

**Fig 2:**
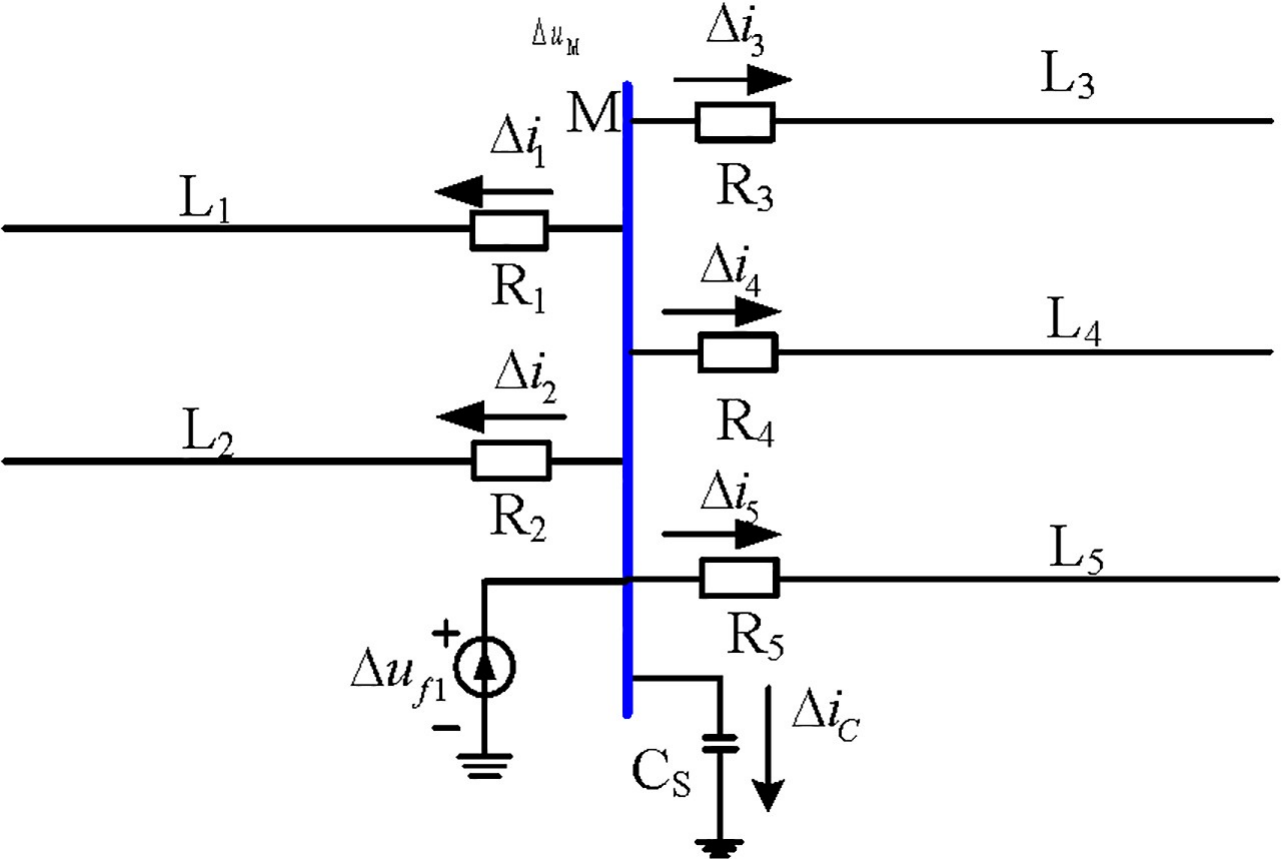
Internal fault additional network.

The positive direction of the current is defined as the direction of the busbar flow to the line. When a fault occurs at F_1_ on the busbar, the additional fault network is the additional voltage at F_1,_ as shown in Fig 2, and the fault component current flows through the protection unit Δ*i*_*m*_ (*m* = 1,2,3,4,5).

When an internal fault occurs, as shown from Table 1 and Eq (3), the current polarities of the busbar branches are the same.

$$\{\begin{array}{c} \Delta u\times (\Delta i_{1}+\Delta i_{2}+\Delta i_{3}+\Delta i_{4})>0\\ \Delta u\times \Delta i_{5}>0 \end{array}$$ (4)

According to Eq (4), this is the same as

$$(\Delta i_{1}+\Delta i_{2}+\Delta i_{3}+\Delta i_{4})\times \Delta i_{5}>0$$ (5)

#### Analysis of the fault characteristics of a busbar external fault

**Fig 3:**
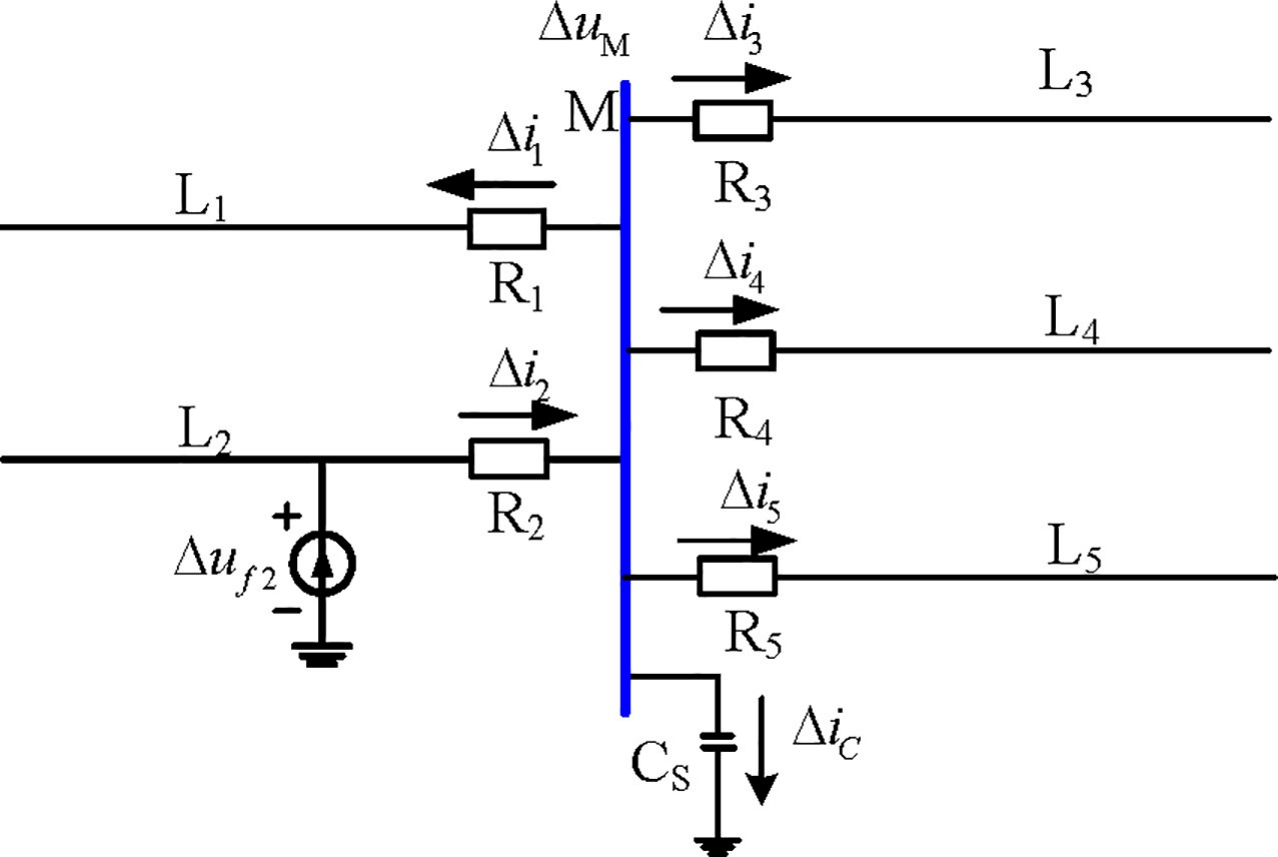
External fault additional network.

Taking the L_2_ line fault as an example, the fault attachment network is shown in Fig 3, which is the additional voltage at F_2_ of the fault point.

From Table 1 and Eqs (1) and (2), the relationship between the current polarities of the busbar branches in Fig 3 can be obtained:

$$\{\begin{array}{c} \begin{array}{l} \Delta u\times \Delta i_{1}>0;\Delta u\times \Delta i_{3}>0;\\ \Delta u\times \Delta i_{4}>0;\Delta u\times \Delta i_{5}>0; \end{array}\\ \Delta u\times \Delta i_{2}<0 \end{array}$$ (6)

If the influence is neglected, according to Kirchhoff's current law and Eq (6), we can obtain:

$$\{\begin{array}{c} \Delta i_{1}+\Delta i_{2}+\Delta i_{3}+\Delta i_{4}\approx -\Delta i_{5}\\ \Delta u\times (\Delta i_{1}+\Delta i_{2}+\Delta i_{3}+\Delta i_{4})\approx -\Delta u\times \Delta i_{5}<0 \end{array}$$ (7)

$$\Rightarrow \{\begin{array}{c} \Delta u\times (\Delta i_{1}+\Delta i_{2}+\Delta i_{3}+\Delta i_{4})<0\\ \Delta u\times \Delta i_{5}>0 \end{array}$$ (8)

The polarity of the current component is opposite to that of the other fault components.

$$(\Delta i_{1}+\Delta i_{2}+\Delta i_{3}+\Delta i_{4})\times \Delta i_{5}<0$$ (9)

The analysis of the fault characteristics of the above busbar gives:

$$\{\begin{array}{c} (\Delta i_{1}+\Delta i_{2}+\Delta i_{3}+\Delta i_{4})\times \Delta i_{5}>0\;\mathrm{Internal}\;\mathrm{fault}\\ (\Delta i_{1}+\Delta i_{2}+\Delta i_{3}+\Delta i_{4})\times \Delta i_{5}<0\;\mathrm{External}\;\mathrm{fault} \end{array}$$ (10)

It should be pointed out that the polarity of the current is determined by the setting of the fault current from the busbar to the line.

## Busbar protection criterion based on a current polarity comparison

From the above analysis, we can accurately determine the fault area. If the reference current is the fault component current of line L_5_, the virtual current is the sum of the fault components of lines L_1_~L_4_. Then,

$$\{\begin{array}{c} \Delta i_{r}=\Delta i_{5}\;\left(\mathrm{reference}\;\mathrm{current}\right)\\ \Delta i_{v}=\Delta i_{1}+\Delta i_{2}+\Delta i_{3}+\Delta i_{4}\;\left(\mathrm{virtual}\;\mathrm{curren}\right) \end{array}$$ (11)

The fault area can be accurately judged by the polarity relationship between the reference current and the virtual current.

The traditional travelling wave busbar protection principle uses the maximum value of the wavelet modulus to characterize the travelling wave polarity, but in the case of a slow wave front, the maximum value of the wavelet mode is small, and it is easily affected by noise interference [21]. In this paper, a new polarity comparison method is proposed that uses the angle between the reference current and the virtual current to realize the polarity comparison.

Wavelet analysis, as a tool for time-frequency analysis, has been very suitable for power system fault signal processing in recent years [22]. The two-input discrete wavelet transform (DWT) of the signal is defined as:

$$d_{j}\left(k\right)=<x\left(t\right),\psi_{j,k}\left(t\right)>,\;j,k\in Z$$ (12)

In Eq (12), *ψ*_*j*,*k*_(*t*) = 2^*j*/2^*ψ*(2^*j*^*t*−*k*) is the discrete wavelet function family for the mother wavelet, and j is the scale coefficient.

Supposing that the discrete sampling of signal *x*(*t*) is *c*_0_(*n*), then the approximate coefficient *c*_*j*_(*n*) and detail coefficient for the j-th scale can be obtained by the Mallat fast algorithm [23].

$$\{\begin{array}{l} c_{j}\left(n\right)=\sum_{k}h\left(k-2n\right)c_{j-1}\left(k\right)\\ d_{j}\left(n\right)=\sum_{k}g\left(k-2n\right)c_{j-1}\left(k\right) \end{array}$$ (13)

In (13), *h*(*n*) and *g*(*n*) are the coefficients for the wavelet decomposition filters, determined by the mother wavelet *ψ*(*t*). The discrete wavelet transform (DWT) is a multiresolution analysis process in which signals are decomposed into components of different frequency bands after the DWT. Single factor reconstruction of the approximate coefficients and detailed coefficients can provide information about signals in different frequency bands.

There will be a transient component in the current after the fault; hence, a discrete wavelet transform is performed on the reference current Δ*i*_*r*_ and virtual current Δ*i*_*v*_. The wavelet decomposition coefficient containing the fundamental frequency component is reconstructed, supposing *C*_*jr*_ and *C*_*jv*_ are the reference current reconstruction coefficient and virtual current reconstruction coefficient for the j-th scale containing the fundamental component.

The angle between *C*_*jr*_ and *C*_*jv*_, *θ* can be calculated with Eq (14) [24]:

$$\theta =\arccos(\frac{C_{jr}\cdot C_{jv}}{\left|C_{jr}\right|\left|C_{jv}\right|})$$ (14)

In (14), *C*_*jr*_⋅*C*_*jv*_ is the dot product of *C*_*jr*_ and *C*_*jv*_, and |*C*_*jr*_| and |*C*_*jv*_|are the 2-norms of *C*_*jr*_ and *C*_*jv*_ respectively.

The angle between *C*_*jr*_ and *C*_*jv*_ is different for different polarities. If the polarity of *C*_*jr*_ and *C*_*jv*_ are the same, the waves will be similar in a certain period, and the angle *θ* will be nearly 0°. If the polarities of Δ*i*_*rs*_ and Δ*i*_*vs*_ are opposite, the waves will be opposite, and *θ* will be nearly 180°. A criterion of the busbar fault area is established according to the angle *θ*.

The fault current flow into the busbar is positive if

$$\theta <\theta_{set}$$ (15)

and the judgement is that it is an internal fault; otherwise, it is an external fault. In (15), *θ*_*set*_ is the threshold value. *θ*_*set*_ is selected as *π*/2 considering the fault characteristics and criterion sensitivity of internal or external faults.

## Implementation of the current polarity comparison busbar protection algorithm

### Algorithm implementation process

**Fig 4:**
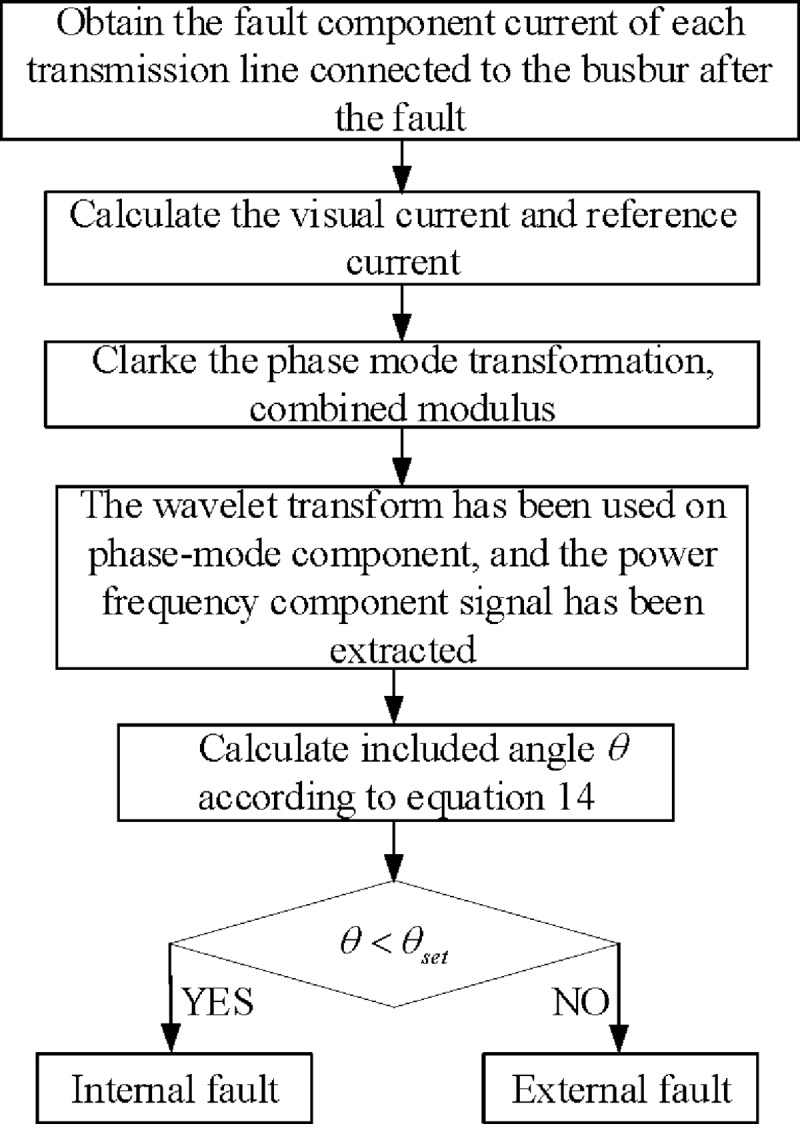
Busbar protection algorithm flow based on the current polarity comparison.

According to the criterion of the current polarity comparison, the specific implementation of the algorithm is proposed, as shown in Fig 4.

1. Data sampling

Obtain the fault travelling wave current for a short time after the failure. Since the proposed algorithm does not need to extract high-frequency transient components, it does not require an excessive sampling rate. The sampling frequency in this article is set as 20 kHz.

2. Phase mode transformation

For a three-phase transmission system, there is coupling between the voltages and currents of each phase. To avoid the influence of the coupling between phases, a phase mode transformation is usually used for decoupling. In this paper, the Clarke transform matrix of the voltage and current in [21] is used.

$$\left[\begin{array}{c} u_{0}\\ u_{\alpha }\\ u_{\beta } \end{array}\right]=\frac{1}{3}\left[\begin{array}{ccc} 1 & 1 & 1\\ 2 & -1 & -1\\ 0 & \sqrt{3} & -\sqrt{3} \end{array}\right]\left[\begin{array}{c} u_{a}\\ u_{b}\\ u_{c} \end{array}\right]$$ (16)

$$\left[\begin{array}{c} i_{0}\\ i_{\alpha }\\ i_{\beta } \end{array}\right]=\frac{1}{3}\left[\begin{array}{ccc} 1 & 1 & 1\\ 2 & -1 & -1\\ 0 & \sqrt{3} & -\sqrt{3} \end{array}\right]\left[\begin{array}{c} i_{a}\\ i_{b}\\ i_{c} \end{array}\right]$$ (17)

The use of single modulus analysis may lead to almost no transient travelling wave components being observed under certain faults, which will cause the protection to fail in a certain case. This is not allowed in HV power grids. To improve the sensitivity of the fault diagnosis, the combined modulus method is adopted in this paper. The voltage and current combinatorial moduli [16] are:

$$\Delta i_{m}=4\Delta i_{\alpha }+\Delta i_{\beta }$$ (18)

3. Extraction of the current signal of the fundamental frequency in the time domain

Multiresolution analysis of the fault travelling wave current is carried out by the wavelet transform with a decomposition into sub-band space. When the sampling frequency is 20 kHz, the seventh layer approximation coefficient contains the fundamental frequency component (0~78.125 Hz). The approximate coefficients of the layer are reconstructed to obtain the signal components of the corresponding fundamental frequency components. Because Daubechies (DB) wavelets have compact support properties and higher-order vanishing moments, DB wavelets are widely used in power system fault signal analysis. In this paper, DB4 wavelets are selected as the wavelet base.

4. Fault region judgement

After calculating the reference current and virtual current, the length of the data window is selected as 1 ms, and the angle between the currents Δ*i*_*rs*_ and Δ*i*_*vs*_, *θ*, is obtained with Eq (14). If Eq (15) is satisfied, the fault is within the area; otherwise, it is outside the area.

### Selection of the threshold value

The threshold selection affects the sensitivity of the protection and is crucial to the protection criterion. For the protection criterion of this paper, the sensitivity of the criterion is reduced for a fault outside the area if the threshold value is too large and for a fault in the area if the threshold value is too small. From the above theoretical analysis, it can be seen that when the fault occurs in the area, *θ* is close to 0°, and when the fault is outside the area, it is nearly 180°.

In practical engineering, there is a loss of sampling point data. From the analysis of Eq (14), it can be seen that:

When a busbar internal fault occurs, *C*_*jr*_⋅*C*_*jv*_>0, |*C*_*jr*_|⋅|*C*_*jv*_|>0, and $\theta <\frac{\pi }{2}$. If the sampling point of the reference current Δ*i*_5_ is lost, *C*_*jr*_⋅*C*_*jv*_ and |*C*_*jr*_|⋅|*C*_*jv*_| decrease. According to Eq (4), after an internal fault, the polarity of the reference current is the same as that of the virtual current. Because the situation in which all of the reference current sampling points are lost will almost never occur, *C*_*jr*_⋅*C*_*jv*_ and will always be larger than 0.
  According to the analysis above: $\frac{C_{jr}\cdot C_{jv}}{\left|C_{jr}\right|\cdot \left|C_{jv}\right|}>0$, and $\theta <\frac{\pi }{2}$. Similarly, for a loss of virtual current sampling points in internal faults, $\frac{C_{jr}\cdot C_{jv}}{\left|C_{jr}\right|\cdot \left|C_{jv}\right|}>0$, and $\theta <\frac{\pi }{2}$.
When a busbar external fault occurs, *C*_*jr*_⋅*C*_*jv*_<0, |*C*_*jr*_|⋅|*C*_*jv*_|<0, and $\theta <\frac{\pi }{2}$. If the virtual current occurrence sampling point is lost, *C*_*jr*_⋅*C*_*jv*_ will increase, and |*C*_*jr*_|⋅|*C*_*jv*_| will decrease. According to Eq (6), the polarities of the virtual current and the reference current will be opposite when an external fault occurs. Supposing that the situation in which all 20 sampling points are lost will not occur, *C*_*jr*_⋅*C*_*jv*_ will always be smaller than 0, and |*C*_*jr*_|⋅|*C*_*jv*_| will always be larger than 0. According to the analysis above: $\frac{C_{jr}\cdot C_{jv}}{\left|C_{jr}\right|\cdot \left|C_{jv}\right|}<0$, and $\theta >\frac{\pi }{2}$. Similarly, the following can be derived by analysing the sampling point lost when a busbar external fault occurs and $\theta >\frac{\pi }{2}$.

Based on the above analysis, $\theta <\frac{\pi }{2}$ when an internal fault occurs, and $\theta >\frac{\pi }{2}$ when an external fault occurs. In this paper, the threshold value is selected as $\theta_{set}=\frac{\pi }{2}$. Many simulations are performed to verify the correctness of the threshold selection.

## Simulation analysis

### Simulation model and parameters

To verify the reliability of the proposed algorithm for different failure initial angles, different fault resistances and different fault types, corresponding simulations were performed to verify the proposed algorithm, and the results are shown in Tables 2–7.

**Table 2:** Test results of the protection algorithm for different initial angles when there is a fault within the busbar.

Fault location Fault type	Fault resistance/Ω	Fault initial angles /(°)	*θ*	Test result
B phase to ground fault (F_1_) occurring on busbar M	100	5	0.50	Internal
		15	0.38	Internal
		45	0.080	Internal
		90	0.090	Internal
		120	0.027	Internal
AB phase to ground fault (F_1_) occurring on busbar M	300	5	0.016	Internal
		15	0.014	Internal
		45	0.0076	Internal
		90	0.025	Internal
		120	0.0032	Internal

**Table 3:** Test results of the protection algorithm for different fault resistances for internal fault cases.

Fault location	Fault resistance /Ω	*θ*	Test result
A phase to ground fault (F_1_) occurring on busbar M (fault initial angle of 45°)	0	0.011	Internal fault
	200	0.021	Internal
	500	0.043	Internal
	800	0.070	Internal

**Table 4:** Test results of the protection algorithm for different fault positions and fault types for internal faults.

Fault location	Type of fault	*θ*	Test result
A fault (F_1_) occurring on busbar M, fault resistance of 300 Ω (Fault initial angle of 60°)	AG	0.033	Internal
	BCG	0.0095	Internal
	AB	0.010	Internal
	ABC	0.0059	Internal

**Table 5:** Test results of the protection algorithm for different fault initial angles for external fault cases.

Fault location Fault type	Fault resistance /Ω	Fault initial angle /(°)	*θ*	Test result
B phase to ground fault occurring at F_2_ on transmission line L_2_ at a distance of 80 km from busbar M	100	5	3.03	External
		15	3.09	External
		45	3.13	External
		90	3.14	External
		120	3.14	External
AB phase to ground fault occurring at F_3_ on transmission line L_4_ at a distance of 50 km from busbar M	150	5	3.14	External
		15	3.14	External
		45	3.14	External
		90	3.14	External
		120	3.14	External

**Table 6:** Test results of the protection algorithm for different fault resistances for external fault cases.

Fault location	Fault resistance /Ω	*θ*	Test result
BC phase to ground fault occurring on transmission line L_2_ at a distance of 100 km from busbar M (fault initial angle of 45°)	0	3.14	External
	200	3.14	External
	500	3.13	External
	800	3.12	External
A phase to ground fault occurring on transmission line L_4_ at a distance of 10 km from busbar M (fault initial angle of 60°)	0	3.14	External
	200	3.14	External
	500	3.14	External
	800	3.14	External

**Table 7:** Test results of the protection algorithm for different fault locations and with different fault types for external fault cases.

Fault location	Type of fault	*θ*	Test result
A fault occurring on transmission line L_2_ at a distance of 20 km from busbar M, fault resistance of 80 Ω (F_2_) (fault initial angle of 60°)	AG	3.14	External
	ABG	3.14	External
	BC	3.13	External
	ABC	3.14	External
A fault occurring on transmission line L_4_ at a distance of 120 km from busbar M, fault resistance of 150 Ω (F_3_) (fault initial angle of 60°)	AG	3.14	External
	ABG	3.14	External
	BC	3.13	External
	ABC	3.14	External

### Simulation test results of a busbar fault

**Fig 5:**
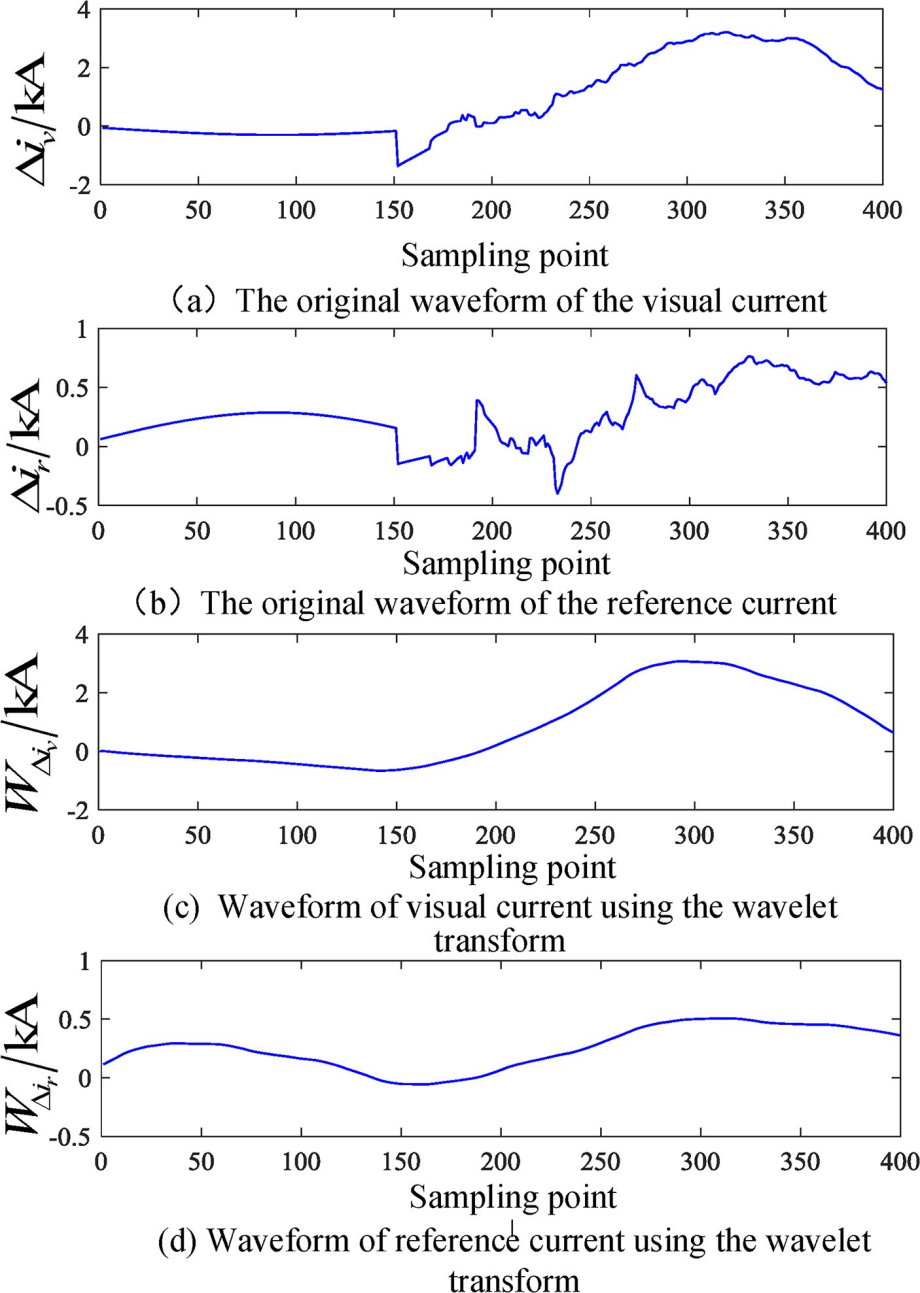
The related current waveform in an internal fault of the busbar.

**Fig 6:**
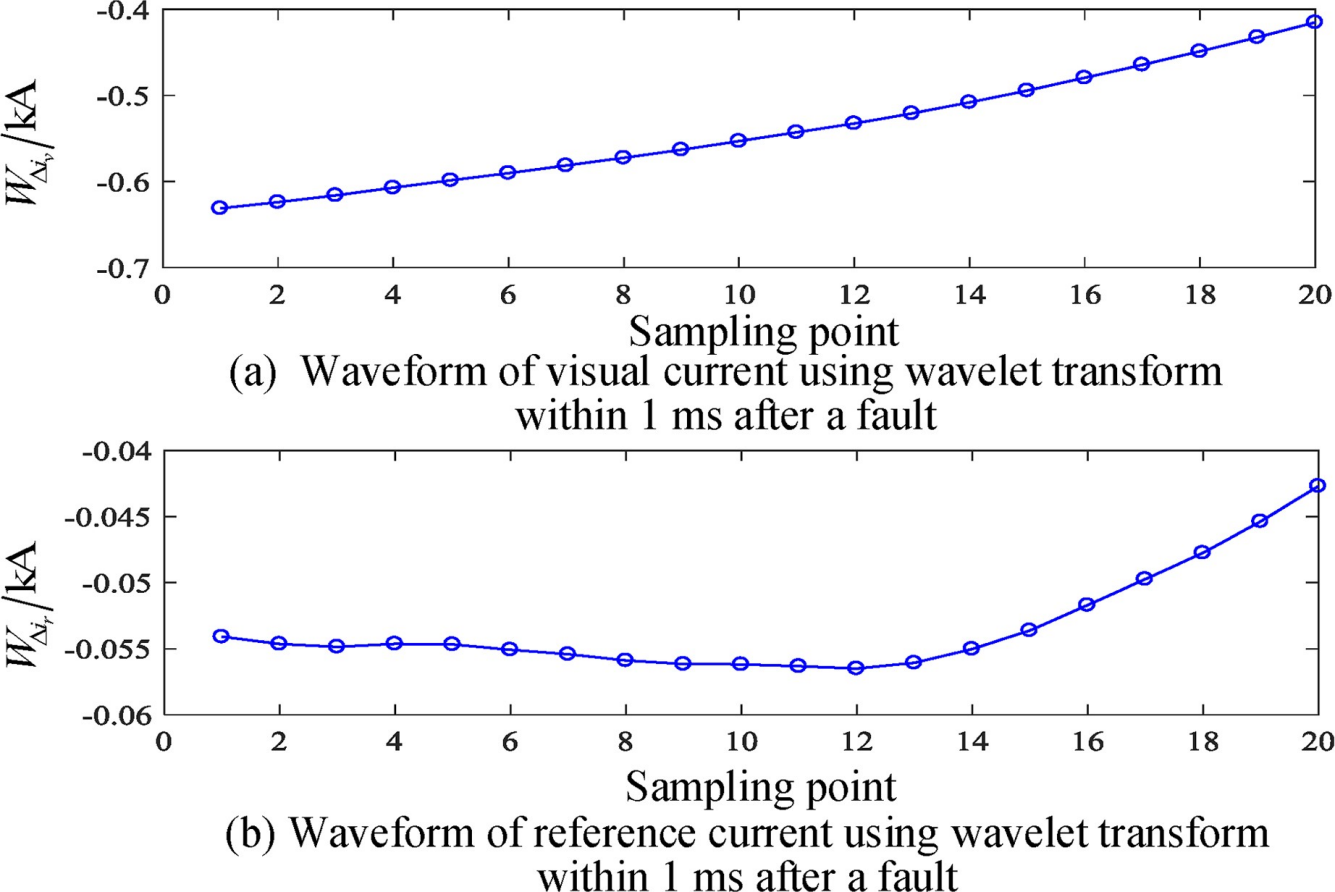
Waveform of correlation current in 1 ms after internal fault of busbar.

The busbar M is set up with an A phase grounding fault, the initial angle of fault is 45°, the fault resistance is 200 Ω, and the correlation waveform is shown in Figs 4–6. Based on the above analysis, the angle between the reference current and the virtual current in the 1 ms data window after failure is calculated.

$$C_{jr}\cdot C_{jv}=0.5783,\;\left|C_{jr}\right|\cdot \left|C_{jv}\right|=0.5801,\;\theta =0.0797rad$$

Table 2 shows the test results of different initial angles for internal faults. The simulation results show that, with the change in the initial fault angle, the included angle will change, but it is essentially stable below 0.5 rad. That is, the initial angle is much smaller than the threshold value, which satisfies Eq (15) and is judged to be an internal fault.

Table 3 shows the test results of the protection algorithm under different fault resistances in an internal fault. From the analysis of the simulation results, it can be seen that the angle gradually increases with an increase in the fault resistance, but both angles are less than 0.1 rad; all are smaller than the threshold value, satisfy Eq (15) and are judged to be internal obstacles; that is, the algorithm is basically not influenced by the fault resistance.

Table 4 shows the test results of the internal fault algorithm for different fault types. The analysis shows that the angle will be different for different types of faults, but all the results are smaller than the threshold value, satisfying Eq (15) as internal faults.

In conclusion, the angle between the reference current and virtual current for different faults is basically within the range of 0–1 rad, which is less than the threshold value. That is, the algorithm proposed in this paper can reliably identify faults in the busbar area.

### Simulation test results of a busbar fault

**Fig 7:**
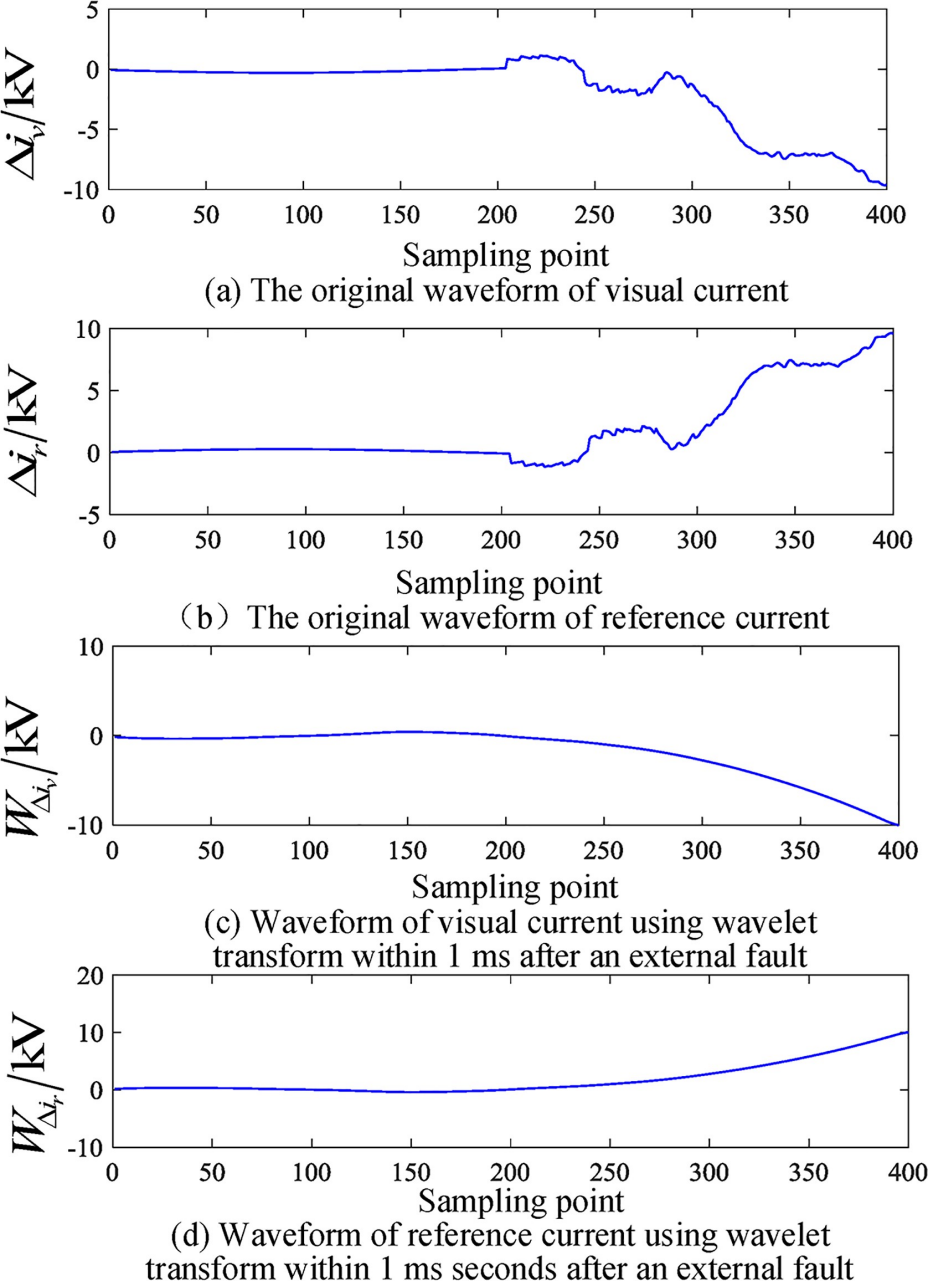
Current waveform of an external fault.

**Fig 8:**
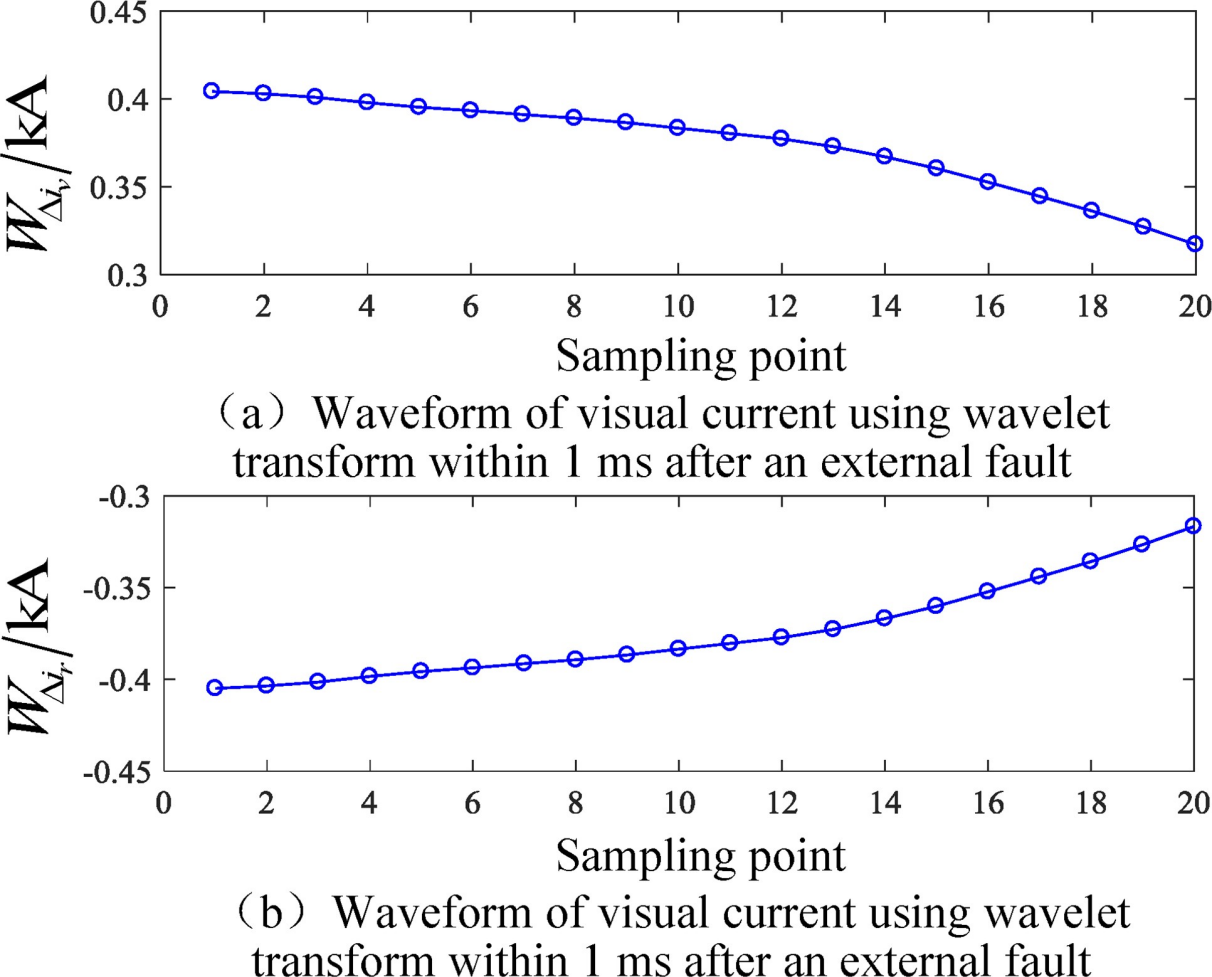
Waveforms of the correlated current over 1 ms after external faults.

An AB phase to ground fault occurs 50 km from busbar M in L4. The initial fault angle is 90°, and the fault resistance is 150 Ω. The related current waveform is shown in Figs 7 and 8. Based on the above analysis and calculation, the initial reactive power in the 1 ms data window after the fault is obtained.

$$C_{jr}\cdot C_{jv}=‑1.3999,\;\left|C_{jr}\right|\cdot \left|C_{jv}\right|=1.3999,\;\theta =3.1408rad$$

To verify the effect of different fault initial angles on the proposed algorithm when external faults occur, a corresponding simulation is performed, and the results are given in Table 5. The simulation results show that when the fault occurs outside the area, the angle between the reference current and virtual current is not affected by the initial angle of the fault, criterion (15) is not satisfied, and the fault is judged to be an external fault.

Table 6 shows the simulation results of the algorithm for different fault resistance. The analysis shows that with a change in the fault resistance, the angle between the reference current and virtual current is basically the same, the value is approximately 3.14, criterion (15) is not satisfied (15), and the fault is reliably judged as an out-of-area fault.

Table 7 shows the simulation results for different fault locations and different fault types when out-of-zone faults are verified. The analysis shows that the angle between the reference current and virtual current is basically unchanged for different fault types, which is always larger than the threshold value and does not satisfy criterion (15); thus, the faults are judged to be external faults.

**Fig 9:**
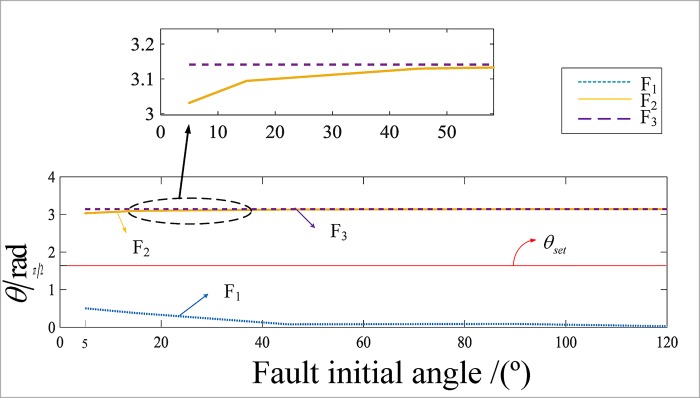
The value of included angle under different fault initial angles when failure occurs.

Fig 9 shows a curve of the angle between the internal and external faults of the busbar within the range of 5° to 120°. F_1_ for busbar M occurs for a B grounding fault, and the fault resistance 100 Ω; F_2_ occurs for a busbar line L_2_ distance from busbar M of80 km with a B phase short circuit; F_3_ occurs for a line L_4_ distance from busbar M of 50 km with an AB phase short circuit. As shown in Fig 9, the difference between the reference current and the virtual current is obvious.

**Fig 10:**
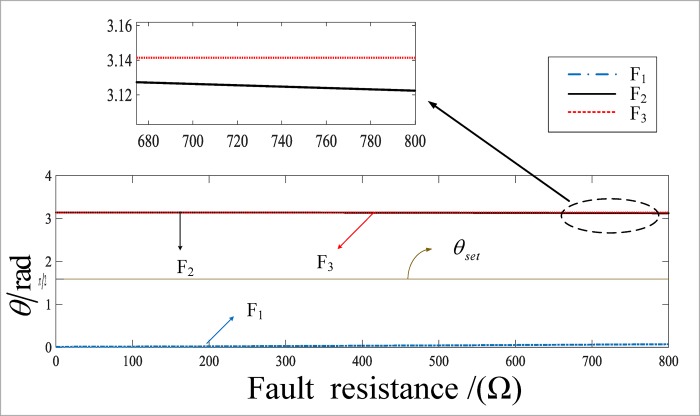
The value of included angle under different fault resistances when failure occurs.

Fig 10 shows a curve of the angle between the internal and external faults in the busbar area when the fault resistance changes in the range of 0 to 800 Ω. F1 has a connection fault for the busbar. The initial angle of the fault is 45°. F_2_ occurs for a line L_2_ distance from the busbar M of 100 km with a BC phase short circuit, and the initial angle of 45°. F_3_ occurs for a line L_4_ distance from the busbar M of 10 km with an ABC phase to ground short circuit, and the initial angle of 60°. It can be seen that the angle of the internal fault is almost zero, and the angle of the external fault is equal to *π*; that is, the difference in the angle between the internal and external faults of the busbar is obvious, and the reliability of the protection criterion is high.

The simulation results show that the proposed algorithm has a distinct difference in the angle when the fault occurs inside and outside the region, which can reliably be used to identify the fault area, and it is not affected by the initial angle of the fault, the fault resistance or the type of fault.

### Algorithm characteristic analysis

#### Resistance to CT saturation

The CT may not change the primary side current correctly because of saturation, which may cause incorrect operation of the protection [25]. However, in the 1/4 fundamental frequency cycle after failure, the CT basically does not produce a saturation phenomenon [26]. To verify the effect of CT saturation on the proposed algorithm, the fault travelling wave current of the 1 ms data window in the 1/20 fundamental frequency cycle after the fault is determined in this paper, as it is not influenced by the effect of CT saturation in theory. Table 8 shows the results of the corresponding simulation experiments to test the anti-CT saturation ability of the algorithm. The CT saturation simulation model adopts a nonlinear time-domain equivalent circuit model with better time-frequency characteristics.

**Table 8:** Test results of the protection algorithm when transmission line l_2_ reaches ct saturation for internal and external busbar faults.

Fault location	Type of fault	*θ*	Test result
A fault occurring on transmission line L_2_ at a distance of 20 km from busbar M, Fault resistance 300 Ω (F_2_) (fault initial angle of 45°)	AG	3.01	External
	ABG	3.11	External
	AC	3.13	External
	ABC	3.12	External
A fault occurring on busbar M, transmission line L_2_ reaches TA saturation, fault resistance of 200 Ω (F_2_) (fault initial angle of 60°)	AG	0.03	Internal
	AB	0.009	Internal
	BCG	0.07	Internal
	ABC	0.007	Internal

From the analysis of the simulation data in the table, it can be seen that the protection criterion can accurately distinguish the fault area whether the CT saturation is caused by the fault in or out of the zone.

#### The effect of the loss of sampling points on the protection algorithm

The loss of sampling data of the reference current and virtual current in an actual engineering measurement influences the analysis of the fault area. For the simulation tests shown in Tables 8 and 9, data are randomly lost for 20 sampling points.

**Table 9:** Test results of the protection algorithm when a number of sampling points are randomly lost for internal busbar faults.

**Sample points of the** **reference** **current randomly lost**	**Fault type**	**The number of sample points randomly lost**	***θ***	**Test result**
	A phase to ground fault occurring on busbar M fault, resistance of 200 Ω	2	0.33	Internal
		4	0.47	Internal
		6	0.59	Internal
		Not data lost	0.02	Internal
**Sample points of the virtual** **current** **randomly lost**	AB phase to ground fault occurring on busbar M, fault resistance of 300 Ω	2	0.48	Internal
		4	0.68	Internal
		6	0.83	Internal
		Not data lost	0.03	Internal

From the simulation results of Tables 9 and 10, it is seen that in the case of a random loss of the data, the loss of the fault data in the area will cause the angle of the inclusion to be larger, and in the area can cause the angle of the inclusion to be smaller. However, even if the data of 6 sampling points are lost, criterion (15) is still satisfied for internal faults, and the criterion is not satisfied for external faults. That is, the protection criterion is basically not affected by the loss of sampled data.

**Table 10:** Test results of the protection algorithm when a number of sample points are randomly lost for external busbar faults.

**Sample points of the** **reference** **current randomly lost**	**Type of** **fault**	**The number of sample points being dropped at random**	***θ***	**Test result**
	A fault occurring at point BC on transmission line L_2_ at a distance of 20 km from busbar M	2	2.55	External
		4	2.32	External
		6	2.15	External
		No data lost	3.13	External
**Sample points of virtual** **current randomly lost**	A phase to ground fault occurring on transmission line L_4_ at a distance of 120 km from busbar M	2	2.81	External
		4	2.66	External
		6	2.54	External
		No data lost	3.14	External

#### Simulation tests under different noise conditions

**Fig 11:**
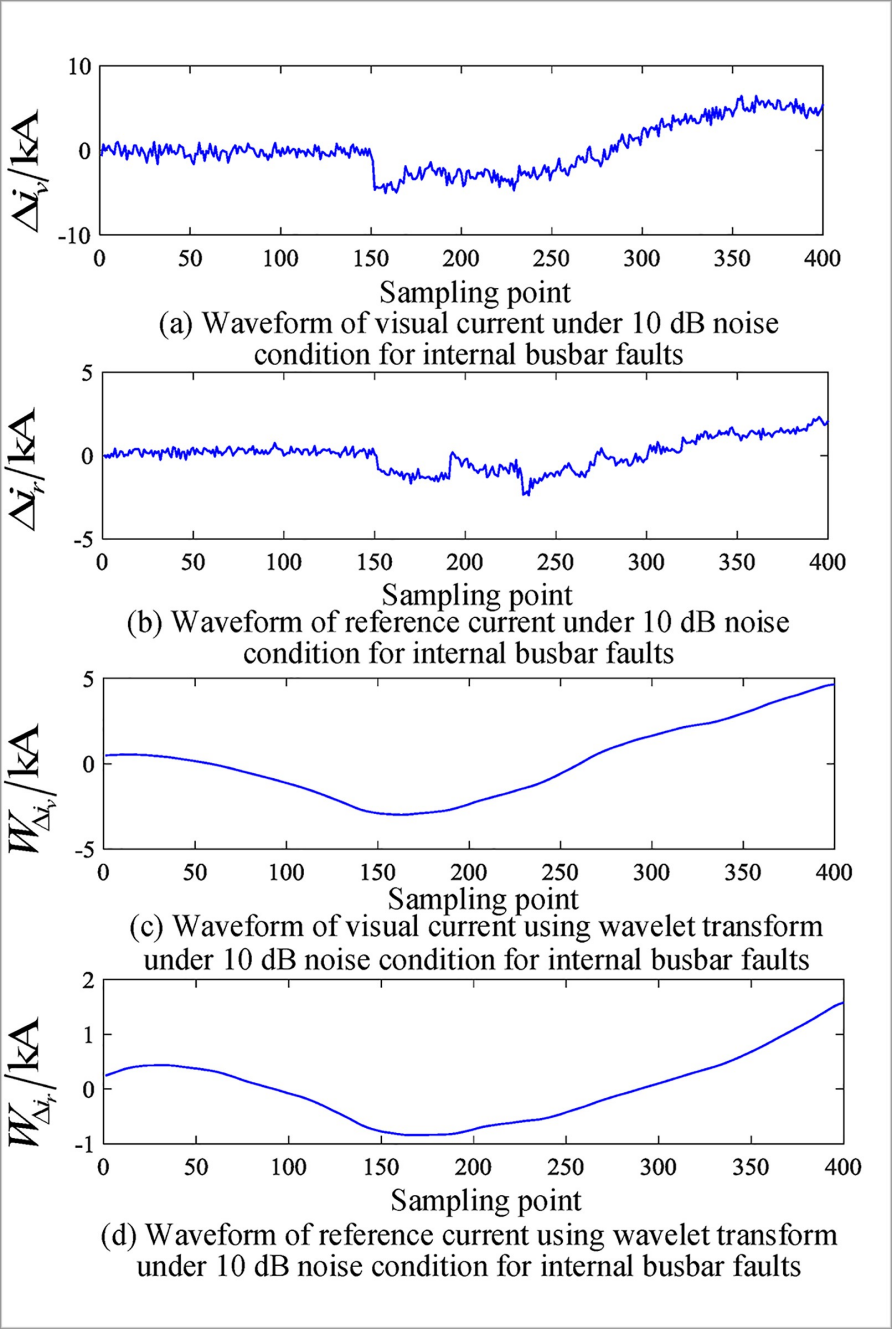
Corresponding current waveforms under 10 db noise conditions for internal busbar faults.

**Fig 12:**
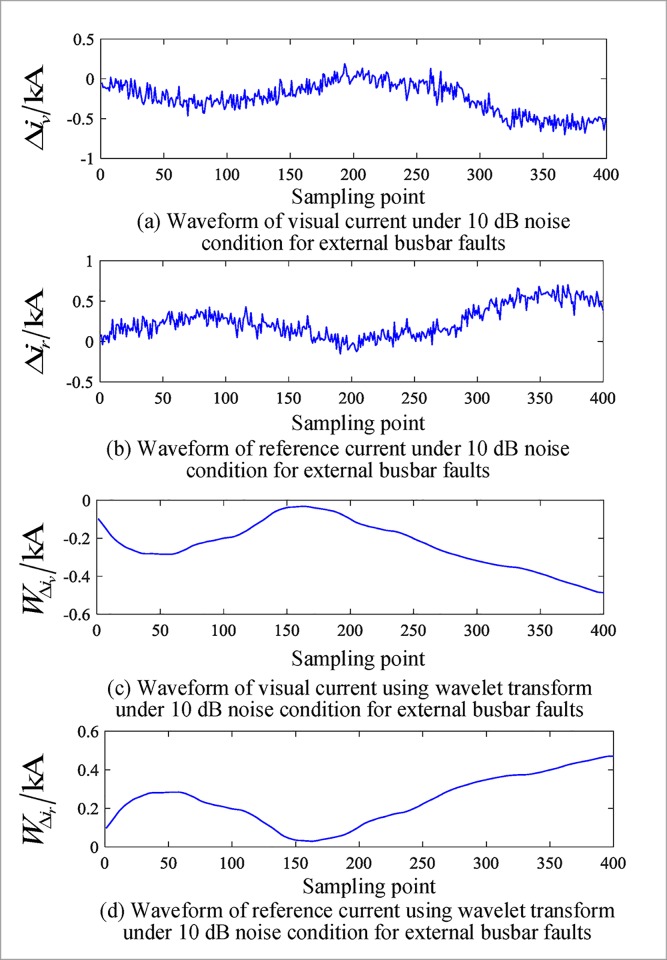
Corresponding current waveforms under 10 db noise conditions for external busbar faults.

The above simulation is based on an ideal simulation signal, and the adaptability of the method is investigated on the basis of noise. Noise of different signal to noise ratios (SNRs) is set up inside and outside the busbar zone, and the partial test results of the algorithm under the influence of different noise intensities are given in Table 11. Figs 11 and 12 show the correlation current waveforms inside and outside the busbar area when the noise ratio is 10 dB.

**Table 11:** Test results of the protection algorithm under different noise conditions for internal and external busbar faults.

Fault location	SNR/dB	*θ*	Test result
A phase to ground fault occurring at on busbar M, fault resistance of 200 Ω (F_3_) (fault initial angle of 45°)	40	0.021	Internal
	30	0.021	Internal
	20	0.021	Internal
	10	0.021	Internal
AB phase to ground short circuit occurring on transmission line L2 at a distance of 80 km from busbar M, fault resistance of 100 Ω (fault initial angle of 90°)	40	3.14	External
	30	3.14	External
	20	3.14	External
	10	3.14	External

Table 11 shows that in the case of noise, the algorithm can still correctly judge the fault area and has a certain noise tolerance, which is consistent with the theoretical analysis.

#### Analysis of the operation speed

Busbar protection widely used in current power systems is based on the current differential protection scheme. Differential busbar protection mainly uses Kirchhoff's current law to detect whether a fault occurs on the busbar. In the scenario of normal operation or when a fault occurs outside the busbar zone, the sum of the currents of the outgoing lines connected to the busbar is 0; when a fault occurs on the busbar, the currents of all the outgoing lines connected to the bus are equal to the total current at the fault point. Current differential protection schemes use a full-circle or half-cycle Fourier algorithm for phasor calculations, the speed of which depends on the computational load of the algorithm and the required data window length. In terms of computational load, when sampling N points per power frequency cycle, applying a full-cycle Fourier algorithm to compute a phasor requires 2N multiplications and additions, while the half-cycle Fourier algorithm requires N multiplications and additions. With a 1600 Hz (32-point sampling) sampling rate, the full-cycle Fourier algorithm of the phasor calculation requires 64 multiplications and additions, and the half-cycle Fourier algorithm requires 32 multiplication algorithms. In terms of data window length, to ensure the accuracy of the calculation, the full-cycle Fourier algorithm requires a data window of 20 ms, while the half-cycle Fourier algorithm requires a data window of 10 ms.

The computational load of the busbar protection algorithm flow (shown in Fig 4) is mainly embodied in the Clarke phase mode transformation, wavelet decomposition and reconstruction, and angle calculation.

After a rough estimation, the phase mode transformation requires 18 multiplications, wavelet decomposition and reconstruction requires approximately 1264 multiplications, and the angle calculation requires 62 multiplications. The total computational load involves approximately 1344 multiplications and a small number of accumulation operations. Fast digital signal processing (DSP) can achieve the above operations. Taking the DS1003 processor based on TMS320C40 as an example, the above operation will not exceed 0.5 ms. If a higher-frequency DSP processor is applied, the operation speed will be faster. The response time is less than 5 ms. Table 12 compares the response times of the traditional current differential protection scheme and the proposed algorithm, where Ta is the protection response time.

**Table 12:** Comparison of protection response times of the busbar protection algorithm.

The current differential protection		The algorithm proposed in this paper
The full-cycle Fourier algorithm	The half-cycle Fourier algorithm	
Ta is greater than 20 ms	Ta is greater than 10 ms	Ta is less than 5 ms

In summary, although the method proposed in this paper is large in terms of computational load, the DSP unit can be applied to complete the operation in 0.5 ms, and the required data window length is only 1 ms, which is greatly shortened compared with that of the traditional power frequency protection algorithm. Therefore, the speed of the reaction of the proposed algorithm will be much higher than that of the traditional power frequency protection scheme.

## Conclusion

In this paper, based on the analysis of the polarity relationship between the associated lines of the busbar, the reference current and the virtual current are defined. By analysing the angle of the two currents, the polarity relationship is determined. A busbar protection algorithm based on the comparison of the fundamental frequency currents is proposed, which solves the problem associated with the slow speed of traditional busbar protection. The reliability of the fault initial angle and the high impedance fault is low, and the transient characteristics of the CVT will be greatly affected. Through theoretical analysis and simulation analysis, the following conclusions can be drawn:

1. When a fault occurs, the reference current has the same polarity as the virtual current. Therefore, the polarity relationship between the reference current and the virtual current can be effectively characterized by the angle between the currents.
2. The busbar protection principle based on the comparison of the polarity of the power frequency currents can detect the fault zone within 1 ms after the fault occurs and has ultra-high speed; the sampling frequency is only 20 kHz, which is relatively low, thus facilitating the implementation of the relay protection component. The algorithm can realize fault zone identification without a fault voltage component and avoids the influence of the CVT transient process on the protection response speed and reaction performance. The proposed algorithm uses the relationship of angles between the reference current and virtual current to characterize the polarity, which can better adapt to the case of a small fault initial angle.
3. The algorithm uses a data window of 1 ms to distinguish the fault area, the speed is fast, and the theoretical analysis and the PSCAD simulation results show that the proposed algorithm can quickly and accurately identify the fault area in various circumstances. The results are not affected by the initial angle of the fault, the fault resistance, the type of fault and the CVT transmission characteristics.

## Supporting information

S1 Fig 500kV busbar model built by PSCAD. (TIF)

S1 Table Test results of the protection algorithm for different initial angles when there is a fault within the busbar. (DOCX)

S2 Table Test results of the protection algorithm for different fault resistances for internal fault cases. (DOCX)

S3 Table Test results of the protection algorithm for different fault positions and fault types for internal faults. (DOCX)

S4 Table Test results of the protection algorithm for different fault initial angles for external fault cases. (DOCX)

S5 Table Test results of the protection algorithm for different fault resistances for external fault cases. (DOCX)

S6 Table Test results of the protection algorithm for different fault locations and with different fault types for external fault cases. (DOCX)

S7 Table Test results of the protection algorithm when transmission line l_2_ reaches ct saturation for internal and external busbar faults. (DOCX)

S8 Table Test results of the protection algorithm when a number of sampling points are randomly lost for internal busbar faults. (DOCX)

S9 Table Test results of the protection algorithm when a number of sample points are randomly lost for external busbar faults. (DOCX)

S10 Table Test results of the protection algorithm under different noise conditions for internal and external busbar faults. (DOCX)

S11 Table The data obtained from Fig 3 and Fig 4 is as follows. (DOCX)

S12 Table The data obtained from Fig 5 and Fig 6 is as follows. (DOCX)

S13 Table The data obtained from Fig 9 and Fig 10 is as follows. (DOCX)

S14 Table The data after wavelet transform of 400 sampling points in Fig 3, Fig 4, Fig 9, and Fig 10 are as follows. (DOCX)
