# Supplementary figures and images for: New principle of busbar protection based on a fundamental frequency polarity comparison
Source: PLoS One. 2019 Mar 21;14(3):e0213308. doi: 10.1371/journal.pone.0213308 (PMC6428346; doi:10.1371/journal.pone.0213308)

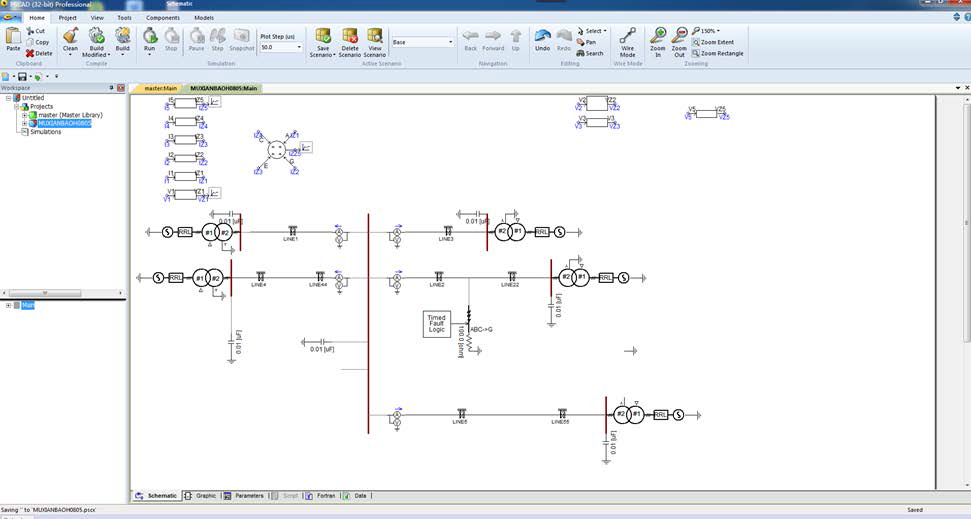

Supplement: S1 Fig — (TIF) [file pone.0213308.s001.tif]
