# Supplementary material for: New principle of busbar protection based on a fundamental frequency polarity comparison
Source: PLoS One. 2019 Mar 21;14(3):e0213308. doi: 10.1371/journal.pone.0213308 (PMC6428346; doi:10.1371/journal.pone.0213308)
Supplement: S1 Table — (DOCX) [file pone.0213308.s002.docx]

| **S1 Table. Test Results of the Protection Algorithm for Different Initial Angles When There is a Fault within the Busbar.** | | | | | | | | | | |
| --- | --- | --- | --- | --- | --- | --- | --- | --- | --- | --- |
| B phase to ground fault (F_1_) occurring on busbar M | | | | | | | | | | |
| The fault initial angle | 5° | | 15° | | 45° | | 90° | | 120° | |
| N-th sampling point after failure | Virtual current(kA) | Reference current(kA) | Virtual current(kA) | Reference current(kA) | Virtual current(kA) | Reference current(kA) | Virtual current(kA) | Reference current(kA) | Virtual current(kA) | Reference current(kA) |
| 1 | -0.989 | -0.0014 | -0.9699 | -0.0203 | -0.6311 | -0.0541 | 0.4755 | 0.1468 | 1.1638 | 0.1968 |
| 2 | -1.0042 | -0.0062 | -0.9837 | -0.0276 | -0.6239 | -0.0546 | 0.5023 | 0.1529 | 1.1933 | 0.1991 |
| 3 | -1.0195 | -0.0118 | -0.9976 | -0.0354 | -0.616 | -0.0549 | 0.5288 | 0.1586 | 1.2237 | 0.202 |
| 4 | -1.0349 | -0.0178 | -1.0115 | -0.0436 | -0.6072 | -0.0546 | 0.5549 | 0.1636 | 1.2547 | 0.2052 |
| 5 | -1.0504 | -0.0244 | -1.0255 | -0.0521 | -0.5985 | -0.0547 | 0.581 | 0.1684 | 1.2866 | 0.2088 |
| 6 | -1.066 | -0.0311 | -1.0396 | -0.0608 | -0.5901 | -0.0551 | 0.6068 | 0.1728 | 1.3188 | 0.2124 |
| 7 | -1.0817 | -0.0379 | -1.0539 | -0.0693 | -0.5813 | -0.0554 | 0.6328 | 0.1771 | 1.3514 | 0.2161 |
| 8 | -1.0975 | -0.0453 | -1.0682 | -0.0775 | -0.5725 | -0.0559 | 0.659 | 0.1814 | 1.3847 | 0.2202 |
| 9 | -1.1134 | -0.053 | -1.0827 | -0.0856 | -0.563 | -0.0562 | 0.6849 | 0.1852 | 1.4186 | 0.2245 |
| 10 | -1.1295 | -0.0608 | -1.0973 | -0.0935 | -0.553 | -0.0562 | 0.7107 | 0.1887 | 1.4528 | 0.2289 |
| 11 | -1.1456 | -0.0686 | -1.112 | -0.1016 | -0.5429 | -0.0563 | 0.737 | 0.1924 | 1.4872 | 0.2332 |
| 12 | -1.1619 | -0.0765 | -1.1268 | -0.1098 | -0.5325 | -0.0565 | 0.7638 | 0.1963 | 1.5221 | 0.2376 |
| 13 | -1.1783 | -0.0846 | -1.1417 | -0.1178 | -0.5209 | -0.0561 | 0.7908 | 0.2001 | 1.5572 | 0.242 |
| 14 | -1.1948 | -0.0931 | -1.1568 | -0.1257 | -0.508 | -0.055 | 0.8181 | 0.204 | 1.593 | 0.2467 |
| 15 | -1.2113 | -0.1021 | -1.1719 | -0.134 | -0.4943 | -0.0536 | 0.8454 | 0.2076 | 1.6295 | 0.2518 |
| 16 | -1.228 | -0.1113 | -1.1871 | -0.1427 | -0.4795 | -0.0517 | 0.8725 | 0.2108 | 1.6665 | 0.257 |
| 17 | -1.2449 | -0.1208 | -1.2023 | -0.1516 | -0.4643 | -0.0497 | 0.8997 | 0.2139 | 1.7041 | 0.2625 |
| 18 | -1.2618 | -0.1301 | -1.2177 | -0.1609 | -0.4488 | -0.0477 | 0.9271 | 0.2168 | 1.7416 | 0.2677 |
| 19 | -1.2789 | -0.139 | -1.2332 | -0.1701 | -0.4324 | -0.0454 | 0.9549 | 0.22 | 1.7789 | 0.2725 |
| 20 | -1.2961 | -0.1479 | -1.2488 | -0.1792 | -0.4152 | -0.0427 | 0.9832 | 0.2234 | 1.8165 | 0.2773 |
| *θ* | 0.50 | | 0.38 | | 0.08 | | 0.09 | | 0.027 | |
| AB phase to ground fault (F_1_) occurring on busbar M | | | | | | | | | | |
| The fault initial angle | 5° | | 15° | | 45° | | 90° | | 120° | |
| N-th sampling point after failure | Virtual current(kA) | Reference current(kA) | Virtual current(kA) | Reference current(kA) | Virtual current(kA) | Reference current(kA) | Virtual current(kA) | Reference current(kA) | Virtual current(kA) | Reference current(kA) |
| 1 | -9.1991 | -2.4441 | -9.6536 | -2.6049 | -7.7289 | -2.2016 | 0.6854 | 0.2092 | 4.8023 | 1.2893 |
| 2 | -9.4033 | -2.5052 | -9.8676 | -2.6715 | -7.7656 | -2.2154 | 0.7947 | 0.2402 | 4.9241 | 1.3195 |
| 3 | -9.6161 | -2.5695 | -10.0869 | -2.7402 | -7.7978 | -2.2276 | 0.901 | 0.2699 | 5.0514 | 1.3515 |
| 4 | -9.8344 | -2.6358 | -10.3119 | -2.8109 | -7.8238 | -2.2378 | 1.0036 | 0.2981 | 5.1821 | 1.3846 |
| 5 | -10.0602 | -2.7049 | -10.54 | -2.8828 | -7.8525 | -2.2491 | 1.1053 | 0.3257 | 5.3176 | 1.4194 |
| 6 | -10.2884 | -2.7748 | -10.7721 | -2.9561 | -7.8851 | -2.2618 | 1.2047 | 0.3523 | 5.4545 | 1.4545 |
| 7 | -10.5188 | -2.8455 | -11.0027 | -3.0287 | -7.9162 | -2.2741 | 1.3041 | 0.3787 | 5.5928 | 1.49 |
| 8 | -10.756 | -2.9187 | -11.231 | -3.1002 | -7.9483 | -2.2869 | 1.4038 | 0.4052 | 5.7355 | 1.527 |
| 9 | -10.999 | -2.994 | -11.4603 | -3.172 | -7.9771 | -2.2986 | 1.5011 | 0.4305 | 5.8818 | 1.5651 |
| 10 | -11.2432 | -3.0696 | -11.689 | -3.2434 | -8.0024 | -2.3092 | 1.5963 | 0.455 | 6.0288 | 1.6033 |
| 11 | -11.4888 | -3.1456 | -11.9198 | -3.3154 | -8.0283 | -2.3201 | 1.6941 | 0.4802 | 6.1766 | 1.6417 |
| 12 | -11.7374 | -3.2227 | -12.1528 | -3.3882 | -8.0541 | -2.3311 | 1.7946 | 0.5063 | 6.3263 | 1.6807 |
| 13 | -11.988 | -3.3004 | -12.3854 | -3.4607 | -8.0713 | -2.3392 | 1.8955 | 0.5325 | 6.4773 | 1.72 |
| 14 | -12.2447 | -3.3803 | -12.618 | -3.5331 | -8.0797 | -2.3444 | 1.9982 | 0.5591 | 6.6322 | 1.7605 |
| 15 | -12.5079 | -3.4627 | -12.8557 | -3.6073 | -8.0829 | -2.3479 | 2.099 | 0.5849 | 6.7911 | 1.8024 |
| 16 | -12.7746 | -3.5463 | -13.0984 | -3.6834 | -8.0786 | -2.3489 | 2.1977 | 0.6097 | 6.9522 | 1.845 |
| 17 | -13.046 | -3.6315 | -13.3441 | -3.7606 | -8.0729 | -2.3496 | 2.2968 | 0.6344 | 7.1163 | 1.8885 |
| 18 | -13.3156 | -3.716 | -13.5941 | -3.8394 | -8.0661 | -2.35 | 2.3956 | 0.6589 | 7.2792 | 1.9314 |
| 19 | -13.5828 | -3.7992 | -13.8447 | -3.9182 | -8.0535 | -2.3484 | 2.4967 | 0.6842 | 7.4406 | 1.9735 |
| 20 | -13.8511 | -3.8828 | -14.0956 | -3.9971 | -8.0363 | -2.3455 | 2.6007 | 0.7105 | 7.6027 | 2.0158 |
| *θ* | 0.016 | | 0.014 | | 0.0076 | | 0.025 | | 0.0032 | |
