# Supplementary material for: New principle of busbar protection based on a fundamental frequency polarity comparison
Source: PLoS One. 2019 Mar 21;14(3):e0213308. doi: 10.1371/journal.pone.0213308 (PMC6428346; doi:10.1371/journal.pone.0213308)
Supplement: S2 Table — (DOCX) [file pone.0213308.s003.docx]

| **S2 Table. Test Results of the Protection Algorithm for Different Fault Resistances for Internal Fault Cases.** | | | | | | | | |
| --- | --- | --- | --- | --- | --- | --- | --- | --- |
| A phase to ground fault (F_1_) occurring on busbar M (fault initial angle of 45°) | | | | | | | | |
| The Fault Resistances | 0Ω | | 200Ω | | 500Ω | | 800Ω | |
| N-th sampling point after failure | Virtual current(kA) | Reference current(kA) | Virtual current(kA) | Reference current(kA) | Virtual current(kA) | Reference current(kA) | Virtual current(kA) | Reference current(kA) |
| 1 | -6.1934 | -1.741 | -2.9766 | -0.758 | -1.5835 | -0.3386 | -1.0922 | -0.1916 |
| 2 | -6.258 | -1.763 | -2.9861 | -0.7636 | -1.5861 | -0.3421 | -1.093 | -0.1945 |
| 3 | -6.3211 | -1.7845 | -2.9939 | -0.7685 | -1.5877 | -0.3451 | -1.0932 | -0.1972 |
| 4 | -6.382 | -1.8051 | -2.9991 | -0.7725 | -1.5879 | -0.3476 | -1.0925 | -0.1993 |
| 5 | -6.4446 | -1.8264 | -3.0053 | -0.7769 | -1.5886 | -0.3504 | -1.0921 | -0.2018 |
| 6 | -6.5094 | -1.8487 | -3.0129 | -0.7821 | -1.59 | -0.3538 | -1.092 | -0.2047 |
| 7 | -6.5741 | -1.871 | -3.0199 | -0.7872 | -1.591 | -0.357 | -1.0916 | -0.2076 |
| 8 | -6.6397 | -1.8938 | -3.0271 | -0.7925 | -1.592 | -0.3604 | -1.0913 | -0.2106 |
| 9 | -6.7044 | -1.9162 | -3.033 | -0.7973 | -1.5923 | -0.3636 | -1.0904 | -0.2134 |
| 10 | -6.768 | -1.9382 | -3.0373 | -0.8016 | -1.5917 | -0.3664 | -1.089 | -0.216 |
| 11 | -6.8324 | -1.9607 | -3.0419 | -0.8062 | -1.5912 | -0.3695 | -1.0876 | -0.2187 |
| 12 | -6.8973 | -1.9833 | -3.0462 | -0.8107 | -1.5906 | -0.3725 | -1.0861 | -0.2215 |
| 13 | -6.959 | -2.0047 | -3.0471 | -0.814 | -1.5881 | -0.3748 | -1.0833 | -0.2237 |
| 14 | -7.0176 | -2.0249 | -3.0445 | -0.816 | -1.5837 | -0.3763 | -1.0793 | -0.2253 |
| 15 | -7.0744 | -2.0444 | -3.0397 | -0.8172 | -1.5781 | -0.3773 | -1.0746 | -0.2266 |
| 16 | -7.1286 | -2.0629 | -3.0318 | -0.8173 | -1.5709 | -0.3776 | -1.0688 | -0.2273 |
| 17 | -7.1828 | -2.0814 | -3.0233 | -0.8173 | -1.5633 | -0.3779 | -1.0627 | -0.2281 |
| 18 | -7.237 | -2.1 | -3.0142 | -0.8172 | -1.5554 | -0.3782 | -1.0563 | -0.2288 |
| 19 | -7.2893 | -2.1179 | -3.0028 | -0.8163 | -1.5462 | -0.3779 | -1.0491 | -0.2292 |
| 20 | -7.3402 | -2.1352 | -2.9894 | -0.8147 | -1.5359 | -0.3773 | -1.0412 | -0.2292 |
| *θ* | 0.011 | | 0.021 | | 0.043 | | 0.070 | |
