# Supplementary material for: New principle of busbar protection based on a fundamental frequency polarity comparison
Source: PLoS One. 2019 Mar 21;14(3):e0213308. doi: 10.1371/journal.pone.0213308 (PMC6428346; doi:10.1371/journal.pone.0213308)
Supplement: S3 Table — (DOCX) [file pone.0213308.s004.docx]

| **S3 Table. Test Results of the Protection Algorithm for Different Fault Positions and Fault Types for Internal Faults.** | | | | | | | | |
| --- | --- | --- | --- | --- | --- | --- | --- | --- |
| A fault (F1) occurring on busbar M, fault resistance of 300 Ω(Fault initial angle of 60°) | | | | | | | | |
| The Fault Resistances | AG | | BCG | | AB | | ABC | |
| N-th sampling point after failure | Virtual current(kA) | Reference current(kA) | Virtual current(kA) | Reference current(kA) | Virtual current(kA) | Reference current(kA) | Virtual current(kA) | Reference current(kA) |
| 1 | -1.6793 | -0.4115 | -5.0012 | -1.4135 | -4.7221 | -1.3299 | -8.5548 | -2.4908 |
| 2 | -1.6711 | -0.4117 | -4.9748 | -1.4081 | -4.6936 | -1.324 | -8.6781 | -2.5299 |
| 3 | -1.6615 | -0.4113 | -4.9435 | -1.4012 | -4.6605 | -1.3165 | -8.802 | -2.5691 |
| 4 | -1.6507 | -0.4106 | -4.9085 | -1.393 | -4.6236 | -1.3078 | -8.9267 | -2.6084 |
| 5 | -1.6409 | -0.4102 | -4.8764 | -1.3859 | -4.5896 | -1.3001 | -9.0522 | -2.6481 |
| 6 | -1.632 | -0.4101 | -4.8476 | -1.3798 | -4.5588 | -1.2934 | -9.1785 | -2.6881 |
| 7 | -1.6233 | -0.4102 | -4.8196 | -1.374 | -4.5288 | -1.287 | -9.3055 | -2.7285 |
| 8 | -1.6154 | -0.4106 | -4.7941 | -1.369 | -4.5012 | -1.2815 | -9.4334 | -2.7692 |
| 9 | -1.6068 | -0.4108 | -4.7664 | -1.3634 | -4.4714 | -1.2752 | -9.5621 | -2.81 |
| 10 | -1.5975 | -0.4107 | -4.7362 | -1.357 | -4.4392 | -1.2682 | -9.6914 | -2.851 |
| 11 | -1.5887 | -0.4108 | -4.7078 | -1.3511 | -4.4087 | -1.2617 | -9.8216 | -2.8923 |
| 12 | -1.5801 | -0.411 | -4.68 | -1.3455 | -4.3788 | -1.2554 | -9.9525 | -2.9339 |
| 13 | -1.5707 | -0.4109 | -4.6493 | -1.339 | -4.3461 | -1.2483 | -10.0843 | -2.9757 |
| 14 | -1.5604 | -0.4106 | -4.616 | -1.3316 | -4.3107 | -1.2404 | -10.217 | -3.0177 |
| 15 | -1.5497 | -0.4102 | -4.5812 | -1.3239 | -4.2739 | -1.232 | -10.3506 | -3.06 |
| 16 | -1.5383 | -0.4095 | -4.5441 | -1.3154 | -4.2348 | -1.223 | -10.4851 | -3.1025 |
| 17 | -1.5272 | -0.409 | -4.5079 | -1.3072 | -4.1967 | -1.2142 | -10.6204 | -3.1453 |
| 18 | -1.5164 | -0.4086 | -4.473 | -1.2995 | -4.1596 | -1.2059 | -10.7565 | -3.1884 |
| 19 | -1.5053 | -0.4081 | -4.4369 | -1.2914 | -4.1215 | -1.1972 | -10.8934 | -3.2318 |
| 20 | -1.4941 | -0.4076 | -4.4006 | -1.2833 | -4.0831 | -1.1885 | -11.0312 | -3.2755 |
| *θ* | 0.033 | | 0.0095 | | 0.010 | | 0.0059 | |
