# Supplementary material for: New principle of busbar protection based on a fundamental frequency polarity comparison
Source: PLoS One. 2019 Mar 21;14(3):e0213308. doi: 10.1371/journal.pone.0213308 (PMC6428346; doi:10.1371/journal.pone.0213308)
Supplement: S4 Table — (DOCX) [file pone.0213308.s005.docx]

| **S4 Table. Test Results of the Protection Algorithm for Different Fault Initial Angles for External Fault Cases.** | | | | | | | | | | |
| --- | --- | --- | --- | --- | --- | --- | --- | --- | --- | --- |
| B phase to ground fault occurring at F_2_ on transmission line L_2_ at a distance of 80 km from busbar M | | | | | | | | | | |
| The fault initial angle | 5° | | 15° | | 45° | | 90° | | 120° | |
| N-th sampling point after failure | Virtual current(kA) | Reference current(kA) | Virtual current(kA) | Reference current(kA) | Virtual current(kA) | Reference current(kA) | Virtual current(kA) | Reference current(kA) | Virtual current(kA) | Reference current(kA) |
| 1 | -0.0498 | 0.0442 | -0.0287 | 0.024 | 0.0256 | -0.0263 | -0.1023 | 0.1042 | -0.1658 | 0.1701 |
| 2 | -0.0456 | 0.0400 | -0.022 | 0.0173 | 0.0266 | -0.0273 | -0.1067 | 0.1086 | -0.1669 | 0.1713 |
| 3 | -0.0406 | 0.03510 | -0.0147 | 0.0102 | 0.0274 | -0.0281 | -0.1106 | 0.1125 | -0.1684 | 0.173 |
| 4 | -0.0353 | 0.02980 | -0.007 | 0.0027 | 0.0279 | -0.0284 | -0.1139 | 0.1159 | -0.1702 | 0.1749 |
| 5 | -0.0293 | 0.0240 | 0.0009 | -0.0051 | 0.0285 | -0.029 | -0.1171 | 0.1191 | -0.1724 | 0.1772 |
| 6 | -0.0233 | 0.0180 | 0.0091 | -0.0132 | 0.0295 | -0.03 | -0.1198 | 0.1219 | -0.1746 | 0.1795 |
| 7 | -0.0171 | 0.0119 | 0.0171 | -0.0211 | 0.0305 | -0.0309 | -0.1225 | 0.1246 | -0.1768 | 0.1818 |
| 8 | -0.0104 | 0.0053 | 0.0246 | -0.0285 | 0.0316 | -0.0319 | -0.1251 | 0.1273 | -0.1794 | 0.1844 |
| 9 | -0.0033 | -0.0017 | 0.0322 | -0.036 | 0.0325 | -0.0328 | -0.1273 | 0.1295 | -0.1822 | 0.1873 |
| 10 | 0.0038 | -0.0087 | 0.0396 | -0.0432 | 0.0333 | -0.0334 | -0.1292 | 0.1315 | -0.185 | 0.1902 |
| 11 | 0.0109 | -0.0157 | 0.0471 | -0.0506 | 0.0341 | -0.0342 | -0.1312 | 0.1336 | -0.1877 | 0.193 |
| 12 | 0.0182 | -0.0229 | 0.0547 | -0.0581 | 0.035 | -0.035 | -0.1334 | 0.1358 | -0.1905 | 0.1959 |
| 13 | 0.0255 | -0.0302 | 0.0621 | -0.0654 | 0.0354 | -0.0353 | -0.1355 | 0.138 | -0.1933 | 0.1988 |
| 14 | 0.0333 | -0.0379 | 0.0694 | -0.0726 | 0.0353 | -0.0351 | -0.1377 | 0.1403 | -0.1964 | 0.202 |
| 15 | 0.0416 | -0.0461 | 0.0772 | -0.0802 | 0.0348 | -0.0346 | -0.1396 | 0.1422 | -0.1998 | 0.2055 |
| 16 | 0.0502 | -0.0545 | 0.0853 | -0.0882 | 0.0339 | -0.0337 | -0.1411 | 0.1438 | -0.2034 | 0.209 |
| 17 | 0.0590 | -0.0632 | 0.0937 | -0.0964 | 0.033 | -0.0327 | -0.1425 | 0.1453 | -0.2071 | 0.2128 |
| 18 | 0.0676 | -0.0716 | 0.1023 | -0.105 | 0.0321 | -0.0317 | -0.1437 | 0.1466 | -0.2105 | 0.2163 |
| 19 | 0.0758 | -0.0797 | 0.1109 | -0.1135 | 0.0308 | -0.0305 | -0.1452 | 0.1481 | -0.2136 | 0.2195 |
| 20 | 0.0839 | -0.0878 | 0.1195 | -0.1219 | 0.0293 | -0.0289 | -0.1468 | 0.1499 | -0.2167 | 0.2227 |
| *θ* | 3.03 | | 3.09 | | 3.13 | | 3.14 | | 3.14 | |
| AB phase to ground fault occurring at F_3_ on transmission line L_~~4~~_ at a distance of 50 km from busbar M | | | | | | | | | | |
| The fault initial angle | 5° | | 15° | | 45° | | 90° | | 120° | |
| N-th sampling point after failure | Virtual current(kA) | Reference current(kA) | Virtual current(kA) | Reference current(kA) | Virtual current(kA) | Reference current(kA) | Virtual current(kA) | Reference current(kA) | Virtual current(kA) | Reference current(kA) |
| 1 | 1.8213 | -1.8266 | 1.9516 | -1.9573 | 1.8927 | -1.8938 | -0.0812 | 0.0821 | -0.8512 | 0.854 |
| 2 | 1.868 | -1.8733 | 2.0044 | -2.01 | 1.9059 | -1.9069 | -0.1017 | 0.1026 | -0.8712 | 0.8741 |
| 3 | 1.9173 | -1.9226 | 2.0588 | -2.0644 | 1.9177 | -1.9187 | -0.1213 | 0.1221 | -0.8927 | 0.8957 |
| 4 | 1.9682 | -1.9734 | 2.1151 | -2.1205 | 1.9277 | -1.9287 | -0.1396 | 0.1404 | -0.915 | 0.918 |
| 5 | 2.0213 | -2.0265 | 2.1722 | -2.1775 | 1.9387 | -1.9396 | -0.1575 | 0.1584 | -0.9386 | 0.9416 |
| 6 | 2.075 | -2.0802 | 2.2305 | -2.2358 | 1.9511 | -1.9519 | -0.1746 | 0.1755 | -0.9624 | 0.9655 |
| 7 | 2.1293 | -2.1344 | 2.2882 | -2.2934 | 1.9631 | -1.9638 | -0.1915 | 0.1924 | -0.9864 | 0.9896 |
| 8 | 2.1855 | -2.1907 | 2.345 | -2.3501 | 1.9756 | -1.9762 | -0.2084 | 0.2094 | -1.0115 | 1.0147 |
| 9 | 2.2435 | -2.2486 | 2.4019 | -2.407 | 1.9871 | -1.9878 | -0.2245 | 0.2255 | -1.0376 | 1.0408 |
| 10 | 2.3017 | -2.3068 | 2.4585 | -2.4635 | 1.9977 | -1.9982 | -0.2398 | 0.2409 | -1.0636 | 1.0669 |
| 11 | 2.3602 | -2.3652 | 2.5157 | -2.5206 | 2.0085 | -2.0091 | -0.2558 | 0.2568 | -1.0898 | 1.0932 |
| 12 | 2.4196 | -2.4245 | 2.5735 | -2.5783 | 2.0195 | -2.02 | -0.2723 | 0.2734 | -1.1164 | 1.1198 |
| 13 | 2.4794 | -2.4843 | 2.631 | -2.6357 | 2.0279 | -2.0283 | -0.2888 | 0.2899 | -1.1432 | 1.1466 |
| 14 | 2.541 | -2.5458 | 2.6883 | -2.693 | 2.0338 | -2.0341 | -0.3056 | 0.3068 | -1.1709 | 1.1744 |
| 15 | 2.6045 | -2.6092 | 2.7472 | -2.7518 | 2.0381 | -2.0384 | -0.3218 | 0.323 | -1.1997 | 1.2032 |
| 16 | 2.669 | -2.6737 | 2.8077 | -2.8122 | 2.0402 | -2.0405 | -0.3372 | 0.3385 | -1.2289 | 1.2325 |
| 17 | 2.7347 | -2.7394 | 2.869 | -2.8734 | 2.042 | -2.0423 | -0.3525 | 0.3539 | -1.2589 | 1.2624 |
| 18 | 2.7999 | -2.8044 | 2.9316 | -2.9359 | 2.0437 | -2.0439 | -0.3676 | 0.369 | -1.2883 | 1.2919 |
| 19 | 2.864 | -2.8685 | 2.9943 | -2.9985 | 2.0436 | -2.0439 | -0.3833 | 0.3847 | -1.3171 | 1.3207 |
| 20 | 2.9284 | -2.9329 | 3.057 | -3.0611 | 2.0423 | -2.0425 | -0.3997 | 0.4012 | -1.346 | 1.3496 |
| *θ* | 3.14 | | 3.14 | | 3.14 | | 3.14 | | 3.14 | |
