# Supplementary material for: New principle of busbar protection based on a fundamental frequency polarity comparison
Source: PLoS One. 2019 Mar 21;14(3):e0213308. doi: 10.1371/journal.pone.0213308 (PMC6428346; doi:10.1371/journal.pone.0213308)
Supplement: S5 Table — (DOCX) [file pone.0213308.s006.docx]

| **S5 Table. Test Results of the Protection Algorithm for Different Fault Resistances for External Fault Cases.** | | | | | | | | |
| --- | --- | --- | --- | --- | --- | --- | --- | --- |
| BC phase to ground fault occurring on transmission line L_2_ at a distance of 100 km from busbar M (fault initial angle of 45°) | | | | | | | | |
| Type of fault | 0Ω | | 200Ω | | 500Ω | | 800Ω | |
| N-th sampling point after failure | Virtual current(kA) | Reference current(kA) | Virtual current(kA) | Reference current(kA) | Virtual current(kA) | Reference current(kA) | Virtual current(kA) | Reference current(kA) |
| 1 | 0.2439 | -0.2435 | 0.0622 | -0.062 | -0.0354 | 0.0356 | -0.0715 | 0.0718 |
| 2 | 0.2544 | -0.254 | 0.0694 | -0.0691 | -0.0287 | 0.0289 | -0.065 | 0.0653 |
| 3 | 0.265 | -0.2644 | 0.0766 | -0.0763 | -0.0219 | 0.0222 | -0.0583 | 0.0587 |
| 4 | 0.2756 | -0.275 | 0.0839 | -0.0836 | -0.015 | 0.0154 | -0.0514 | 0.0519 |
| 5 | 0.2865 | -0.2858 | 0.0914 | -0.091 | -0.0079 | 0.0083 | -0.0445 | 0.045 |
| 6 | 0.2976 | -0.2969 | 0.0991 | -0.0986 | -0.0006 | 0.0012 | -0.0373 | 0.0379 |
| 7 | 0.3089 | -0.3081 | 0.107 | -0.1064 | 0.0067 | -0.0061 | -0.0301 | 0.0307 |
| 8 | 0.3203 | -0.3195 | 0.115 | -0.1143 | 0.0143 | -0.0136 | -0.0226 | 0.0234 |
| 9 | 0.332 | -0.331 | 0.1231 | -0.1224 | 0.022 | -0.0212 | -0.0151 | 0.0159 |
| 10 | 0.3437 | -0.3427 | 0.1313 | -0.1305 | 0.0298 | -0.0289 | -0.0073 | 0.0082 |
| 11 | 0.3556 | -0.3545 | 0.1396 | -0.1388 | 0.0377 | -0.0368 | 0.0006 | 0.0004 |
| 12 | 0.3677 | -0.3666 | 0.1481 | -0.1472 | 0.0458 | -0.0449 | 0.0086 | -0.0076 |
| 13 | 0.38 | -0.3787 | 0.1567 | -0.1557 | 0.0541 | -0.0531 | 0.0169 | -0.0158 |
| 14 | 0.3923 | -0.391 | 0.1653 | -0.1642 | 0.0626 | -0.0615 | 0.0253 | -0.0242 |
| 15 | 0.4047 | -0.4034 | 0.174 | -0.1729 | 0.0712 | -0.07 | 0.034 | -0.0328 |
| 16 | 0.4172 | -0.4158 | 0.1828 | -0.1816 | 0.08 | -0.0788 | 0.0429 | -0.0417 |
| 17 | 0.4299 | -0.4285 | 0.1917 | -0.1905 | 0.0889 | -0.0877 | 0.052 | -0.0507 |
| 18 | 0.4428 | -0.4414 | 0.2008 | -0.1996 | 0.0981 | -0.0968 | 0.0612 | -0.0599 |
| 19 | 0.4559 | -0.4544 | 0.21 | -0.2087 | 0.1074 | -0.106 | 0.0706 | -0.0692 |
| 20 | 0.4691 | -0.4675 | 0.2193 | -0.218 | 0.1168 | -0.1155 | 0.0802 | -0.0788 |
| *θ* | 3.14 | | 3.14 | | 3.13 | | 3.12 | |
| A phase to ground fault occurring on transmission line L_4_ at a distance of 10 km from busbar M (fault initial angle of 60°) | | | | | | | | |
| Type of fault | 0Ω | | 200Ω | | 500Ω | | 800Ω | |
| N-th sampling point after failure | Virtual current(kA) | Reference current(kA) | Virtual current(kA) | Reference current(kA) | Virtual current(kA) | Reference current(kA) | Virtual current(kA) | Reference current(kA) |
| 1 | 0.6494 | -0.6486 | 0.3659 | -0.3657 | 0.1792 | -0.1789 | 0.1027 | -0.1023 |
| 2 | 0.6599 | -0.6591 | 0.3674 | -0.3671 | 0.1803 | -0.1799 | 0.104 | -0.1035 |
| 3 | 0.6702 | -0.6694 | 0.3683 | -0.368 | 0.1811 | -0.1807 | 0.1051 | -0.1045 |
| 4 | 0.6805 | -0.6796 | 0.369 | -0.3686 | 0.1816 | -0.1812 | 0.1059 | -0.1053 |
| 5 | 0.6909 | -0.69 | 0.37 | -0.3695 | 0.1824 | -0.1819 | 0.1069 | -0.1063 |
| 6 | 0.7016 | -0.7007 | 0.3712 | -0.3708 | 0.1834 | -0.1828 | 0.1082 | -0.1075 |
| 7 | 0.7124 | -0.7114 | 0.3726 | -0.3721 | 0.1845 | -0.1838 | 0.1095 | -0.1087 |
| 8 | 0.7233 | -0.7223 | 0.3743 | -0.3737 | 0.1857 | -0.1851 | 0.1109 | -0.1101 |
| 9 | 0.7342 | -0.7332 | 0.3757 | -0.3751 | 0.1869 | -0.1861 | 0.1123 | -0.1114 |
| 10 | 0.7451 | -0.744 | 0.377 | -0.3763 | 0.1879 | -0.1871 | 0.1135 | -0.1126 |
| 11 | 0.7562 | -0.755 | 0.3784 | -0.3777 | 0.189 | -0.1881 | 0.1148 | -0.1139 |
| 12 | 0.7673 | -0.7661 | 0.3799 | -0.3791 | 0.1901 | -0.1892 | 0.1162 | -0.1152 |
| 13 | 0.7784 | -0.7772 | 0.3812 | -0.3804 | 0.1912 | -0.1902 | 0.1174 | -0.1164 |
| 14 | 0.7896 | -0.7883 | 0.3824 | -0.3815 | 0.1921 | -0.1911 | 0.1186 | -0.1175 |
| 15 | 0.8007 | -0.7994 | 0.3834 | -0.3824 | 0.1929 | -0.1918 | 0.1197 | -0.1186 |
| 16 | 0.8119 | -0.8106 | 0.3842 | -0.3832 | 0.1936 | -0.1925 | 0.1208 | -0.1196 |
| 17 | 0.8232 | -0.8218 | 0.3852 | -0.3842 | 0.1944 | -0.1932 | 0.1219 | -0.1206 |
| 18 | 0.8346 | -0.8332 | 0.3863 | -0.3852 | 0.1953 | -0.1941 | 0.1231 | -0.1218 |
| 19 | 0.8461 | -0.8446 | 0.3873 | -0.3862 | 0.1961 | -0.1948 | 0.1242 | -0.1229 |
| 20 | 0.8576 | -0.8561 | 0.3884 | -0.3872 | 0.197 | -0.1957 | 0.1254 | -0.124 |
| *θ* | 3.14 | | 3.14 | | 3.14 | | 3.14 | |
