# Supplementary material for: New principle of busbar protection based on a fundamental frequency polarity comparison
Source: PLoS One. 2019 Mar 21;14(3):e0213308. doi: 10.1371/journal.pone.0213308 (PMC6428346; doi:10.1371/journal.pone.0213308)
Supplement: S6 Table — (DOCX) [file pone.0213308.s007.docx]

| **S6 Table. Test Results of the Protection Algorithm** **for Different Fault Locations and with Different Fault Types for External Fault Cases.** | | | | | | | | |
| --- | --- | --- | --- | --- | --- | --- | --- | --- |
| A fault occurring on transmission line L_2_ at a distance of 20 km from busbar M, fault resistance of 80 Ω (F_2_) (fault initial angle of 60°) | | | | | | | | |
| Type of fault | AG | | ABG | | BC | | ABC | |
| N-th sampling point after failure | Virtual current(kA) | Reference current(kA) | Virtual current(kA) | Reference current(kA) | Virtual current(kA) | Reference current(kA) | Virtual current(kA) | Reference current(kA) |
| 1 | 1.0918 | -1.0924 | 2.0456 | -2.0434 | -0.1321 | 0.1326 | 2.7532 | -2.7468 |
| 2 | 1.1013 | -1.1019 | 2.0601 | -2.0578 | -0.1235 | 0.1241 | 2.7955 | -2.789 |
| 3 | 1.1101 | -1.1106 | 2.0733 | -2.0709 | -0.1146 | 0.1153 | 2.8373 | -2.8308 |
| 4 | 1.1178 | -1.1182 | 2.0846 | -2.0822 | -0.1053 | 0.106 | 2.8786 | -2.872 |
| 5 | 1.1261 | -1.1265 | 2.0969 | -2.0944 | -0.0959 | 0.0967 | 2.9208 | -2.9141 |
| 6 | 1.1353 | -1.1356 | 2.1106 | -2.108 | -0.0865 | 0.0874 | 2.9642 | -2.9575 |
| 7 | 1.1443 | -1.1446 | 2.1239 | -2.1213 | -0.0768 | 0.0778 | 3.0079 | -3.0011 |
| 8 | 1.1537 | -1.1538 | 2.1377 | -2.1351 | -0.067 | 0.068 | 3.0523 | -3.0454 |
| 9 | 1.1626 | -1.1626 | 2.1506 | -2.1479 | -0.0569 | 0.058 | 3.0965 | -3.0896 |
| 10 | 1.1708 | -1.1708 | 2.1625 | -2.1597 | -0.0465 | 0.0476 | 3.1406 | -3.1336 |
| 11 | 1.1794 | -1.1793 | 2.1747 | -2.1718 | -0.0359 | 0.0371 | 3.1853 | -3.1783 |
| 12 | 1.188 | -1.1879 | 2.187 | -2.1841 | -0.0251 | 0.0264 | 3.2305 | -3.2234 |
| 13 | 1.1951 | -1.195 | 2.1966 | -2.1937 | -0.0138 | 0.0152 | 3.2747 | -3.2676 |
| 14 | 1.2008 | -1.2005 | 2.2037 | -2.2007 | -0.002 | 0.0034 | 3.318 | -3.3108 |
| 15 | 1.2056 | -1.2053 | 2.2091 | -2.2061 | 0.0102 | -0.0088 | 3.3608 | -3.3536 |
| 16 | 1.2091 | -1.2088 | 2.2124 | -2.2093 | 0.023 | -0.0214 | 3.4029 | -3.3957 |
| 17 | 1.2125 | -1.2121 | 2.2153 | -2.2122 | 0.0359 | -0.0344 | 3.4453 | -3.4381 |
| 18 | 1.2158 | -1.2154 | 2.218 | -2.2149 | 0.0492 | -0.0475 | 3.488 | -3.4809 |
| 19 | 1.2182 | -1.2177 | 2.219 | -2.2159 | 0.0628 | -0.0612 | 3.5303 | -3.5231 |
| 20 | 1.2198 | -1.2194 | 2.2187 | -2.2156 | 0.0768 | -0.0751 | 3.5723 | -3.5652 |
| *θ* | 3.14 | | 3.14 | | 3.13 | | 3.14 | |
| A fault occurring on transmission line L_4_ at a distance of 120 km from busbar M, fault resistance of 150 Ω (F_3_) (fault initial angle of 60°) | | | | | | | | |
| Type of fault | AG | | ABG | | BC | | ABC | |
| N-th sampling point after failure | Virtual current(kA) | Reference current(kA) | Virtual current(kA) | Reference current(kA) | Virtual current(kA) | Reference current(kA) | Virtual current(kA) | Reference current(kA) |
| 1 | 0.423 | -0.4226 | 0.8132 | -0.8134 | -0.041 | 0.0423 | 3.4453 | -3.4381 |
| 2 | 0.4249 | -0.4244 | 0.8134 | -0.8135 | -0.0314 | 0.0328 | 3.488 | -3.4809 |
| 3 | 0.4264 | -0.4258 | 0.8127 | -0.8127 | -0.0216 | 0.023 | 3.5303 | -3.5231 |
| 4 | 0.4275 | -0.4269 | 0.8114 | -0.8114 | -0.0115 | 0.0129 | 3.5723 | -3.5652 |
| 5 | 0.4289 | -0.4283 | 0.8106 | -0.8106 | -0.0015 | 0.003 | 3.6151 | -3.608 |
| 6 | 0.4307 | -0.43 | 0.8105 | -0.8104 | 0.0084 | -0.0068 | 3.6586 | -3.6515 |
| 7 | 0.4326 | -0.4319 | 0.8105 | -0.8104 | 0.0183 | -0.0167 | 3.7026 | -3.6955 |
| 8 | 0.4348 | -0.434 | 0.8109 | -0.8108 | 0.0281 | -0.0265 | 3.7472 | -3.7401 |
| 9 | 0.4367 | -0.4359 | 0.811 | -0.8109 | 0.0381 | -0.0364 | 3.7918 | -3.7847 |
| 10 | 0.4385 | -0.4376 | 0.8108 | -0.8105 | 0.0482 | -0.0465 | 3.8363 | -3.8292 |
| 11 | 0.4405 | -0.4395 | 0.8108 | -0.8105 | 0.0583 | -0.0566 | 3.8813 | -3.8742 |
| 12 | 0.4425 | -0.4415 | 0.811 | -0.8106 | 0.0685 | -0.0666 | 3.9267 | -3.9196 |
| 13 | 0.4443 | -0.4433 | 0.8107 | -0.8103 | 0.0788 | -0.0769 | 3.9721 | -3.965 |
| 14 | 0.446 | -0.4449 | 0.8101 | -0.8096 | 0.0894 | -0.0875 | 4.0175 | -4.0104 |
| 15 | 0.4475 | -0.4464 | 0.8092 | -0.8087 | 0.1002 | -0.0982 | 4.063 | -4.0559 |
| 16 | 0.4488 | -0.4477 | 0.808 | -0.8075 | 0.1111 | -0.1091 | 4.1085 | -4.1015 |
| 17 | 0.4503 | -0.4491 | 0.807 | -0.8065 | 0.122 | -0.12 | 4.1545 | -4.1475 |
| 18 | 0.452 | -0.4507 | 0.8062 | -0.8056 | 0.133 | -0.1308 | 4.2009 | -4.1939 |
| 19 | 0.4535 | -0.4522 | 0.8053 | -0.8047 | 0.144 | -0.1418 | 4.2475 | -4.2406 |
| 20 | 0.4551 | -0.4537 | 0.8044 | -0.8037 | 0.1552 | -0.1529 | 4.2945 | -4.2875 |
| *θ* | 3.14 | | 3.14 | | 3.13 | | 3.14 | |
