# Supplementary material for: New principle of busbar protection based on a fundamental frequency polarity comparison
Source: PLoS One. 2019 Mar 21;14(3):e0213308. doi: 10.1371/journal.pone.0213308 (PMC6428346; doi:10.1371/journal.pone.0213308)
Supplement: S7 Table — (DOCX) [file pone.0213308.s008.docx]

| **S7 Table. Test Results of the Protection Algorithm when Transmission Line L_2_ Reaches CT Saturation for Internal and External Busbar Faults.** | | | | | | | | |
| --- | --- | --- | --- | --- | --- | --- | --- | --- |
| A fault occurring on transmission line L_2_ at a distance of 20 km from busbar M, Fault resistance 300 Ω (F_2_) (fault initial angle of 45°) | | | | | | | | |
| Type of fault | AG | | ABG | | BC | | ABC | |
| N-th sampling point after failure | Virtual current(kA) | Reference current(kA) | Virtual current(kA) | Reference current(kA) | Virtual current(kA) | Reference current(kA) | Virtual current(kA) | Reference current(kA) |
| 1 | -0.1633 | 0.0343 | 2.5956 | -1.9567 | 1.8489 | -2.2309 | 1.665 | -2.7467 |
| 2 | -0.161 | 0.033 | 2.6006 | -1.9695 | 1.8912 | -2.2768 | 1.697 | -2.7889 |
| 3 | -0.1588 | 0.0319 | 2.6031 | -1.981 | 1.9339 | -2.3229 | 1.729 | -2.8308 |
| 4 | -0.1566 | 0.031 | 2.6023 | -1.9906 | 1.9771 | -2.3694 | 1.761 | -2.872 |
| 5 | -0.1543 | 0.0299 | 2.6029 | -2.0012 | 2.0208 | -2.4165 | 1.7936 | -2.9141 |
| 6 | -0.1518 | 0.0286 | 2.6056 | -2.0131 | 2.0651 | -2.4644 | 1.8268 | -2.9575 |
| 7 | -0.1492 | 0.0272 | 2.6073 | -2.0247 | 2.11 | -2.5128 | 1.8604 | -3.0011 |
| 8 | -0.1465 | 0.0258 | 2.6096 | -2.0368 | 2.1555 | -2.5618 | 1.8945 | -3.0454 |
| 9 | -0.1437 | 0.0244 | 2.6101 | -2.0479 | 2.2015 | -2.6113 | 1.9287 | -3.0896 |
| 10 | -0.141 | 0.0232 | 2.6087 | -2.0579 | 2.248 | -2.6612 | 1.9631 | -3.1336 |
| 11 | -0.1382 | 0.0219 | 2.6077 | -2.0683 | 2.295 | -2.7117 | 1.9979 | -3.1782 |
| 12 | -0.1353 | 0.0205 | 2.6065 | -2.0788 | 2.3426 | -2.7628 | 2.0332 | -3.2233 |
| 13 | -0.1324 | 0.0194 | 2.6008 | -2.0866 | 2.3908 | -2.8142 | 2.0682 | -3.2675 |
| 14 | -0.1297 | 0.0188 | 2.5906 | -2.0918 | 2.4396 | -2.8659 | 2.1032 | -3.3108 |
| 15 | -0.127 | 0.0184 | 2.5775 | -2.0954 | 2.489 | -2.9181 | 2.1382 | -3.3536 |
| 16 | -0.1243 | 0.0182 | 2.5606 | -2.0967 | 2.539 | -2.9707 | 2.1731 | -3.3957 |
| 17 | -0.1216 | 0.0181 | 2.5429 | -2.0977 | 2.5896 | -3.0238 | 2.2083 | -3.4381 |
| 18 | -0.1188 | 0.018 | 2.5247 | -2.0986 | 2.6407 | -3.0775 | 2.244 | -3.4808 |
| 19 | -0.116 | 0.0181 | 2.5033 | -2.0976 | 2.6925 | -3.1316 | 2.2797 | -3.5231 |
| 20 | -0.1132 | 0.0183 | 2.4796 | -2.0954 | 2.7449 | -3.1863 | 2.3155 | -3.5652 |
| *θ* | 3.01 | | 3.11 | | 3.13 | | 3.12 | |
| A fault occurring on transmission line L_4_ at a distance of 120 km from busbar M, fault resistance of 150 Ω (F_3_) (fault initial angle of 60°) | | | | | | | | |
| Type of fault | AG | | ABG | | BC | | ABC | |
| N-th sampling point after failure | Virtual current(kA) | Reference current(kA) | Virtual current(kA) | Reference current(kA) | Virtual current(kA) | Reference current(kA) | Virtual current(kA) | Reference current(kA) |
| 1 | -2.158 | -0.5554 | -4.8627 | -1.3299 | -1.0776 | -0.2356 | -8.6873 | -2.4908 |
| 2 | -2.1501 | -0.5557 | -4.8339 | -1.324 | -1.1154 | -0.2495 | -8.8099 | -2.5299 |
| 3 | -2.1404 | -0.5553 | -4.8002 | -1.3165 | -1.1548 | -0.2637 | -8.9331 | -2.5691 |
| 4 | -2.1294 | -0.5544 | -4.7628 | -1.3078 | -1.1954 | -0.2783 | -9.057 | -2.6084 |
| 5 | -2.1194 | -0.5539 | -4.7284 | -1.3001 | -1.2353 | -0.2927 | -9.1818 | -2.6481 |
| 6 | -2.1107 | -0.5539 | -4.6972 | -1.2934 | -1.2746 | -0.307 | -9.3074 | -2.6881 |
| 7 | -2.1022 | -0.5541 | -4.6667 | -1.287 | -1.3138 | -0.3214 | -9.4338 | -2.7285 |
| 8 | -2.0947 | -0.5546 | -4.6388 | -1.2815 | -1.3525 | -0.3357 | -9.5611 | -2.7692 |
| 9 | -2.0864 | -0.5548 | -4.6087 | -1.2752 | -1.3921 | -0.3502 | -9.6891 | -2.81 |
| 10 | -2.0772 | -0.5547 | -4.5761 | -1.2682 | -1.4324 | -0.3649 | -9.8178 | -2.851 |
| 11 | -2.0687 | -0.5549 | -4.5451 | -1.2617 | -1.4726 | -0.3796 | -9.9473 | -2.8923 |
| 12 | -2.0604 | -0.5552 | -4.5149 | -1.2554 | -1.5127 | -0.3943 | -10.0776 | -2.9339 |
| 13 | -2.051 | -0.5551 | -4.4817 | -1.2483 | -1.5538 | -0.4093 | -10.2087 | -2.9757 |
| 14 | -2.0407 | -0.5548 | -4.4459 | -1.2404 | -1.5959 | -0.4246 | -10.3406 | -3.0177 |
| 15 | -2.0298 | -0.5543 | -4.4085 | -1.232 | -1.6386 | -0.44 | -10.4734 | -3.06 |
| 16 | -2.0181 | -0.5535 | -4.3689 | -1.223 | -1.6821 | -0.4557 | -10.607 | -3.1025 |
| 17 | -2.0068 | -0.5529 | -4.3301 | -1.2142 | -1.7255 | -0.4715 | -10.7415 | -3.1453 |
| 18 | -1.9959 | -0.5524 | -4.2926 | -1.2059 | -1.7688 | -0.4872 | -10.8767 | -3.1884 |
| 19 | -1.9846 | -0.5519 | -4.2539 | -1.1972 | -1.8127 | -0.5031 | -11.0128 | -3.2318 |
| 20 | -1.9733 | -0.5513 | -4.2149 | -1.1885 | -1.8568 | -0.5191 | -11.1498 | -3.2755 |
| *θ* | 0.03 | | 0.009 | | 0.07 | | 0.007 | |
