# Supplementary material for: New principle of busbar protection based on a fundamental frequency polarity comparison
Source: PLoS One. 2019 Mar 21;14(3):e0213308. doi: 10.1371/journal.pone.0213308 (PMC6428346; doi:10.1371/journal.pone.0213308)
Supplement: S8 Table — (DOCX) [file pone.0213308.s009.docx]

| **S8 Table. Test Results of the Protection Algorithm When a Number of Sampling Points are Randomly Lost for Internal Busbar Faults.** | | | | | | | | |
| --- | --- | --- | --- | --- | --- | --- | --- | --- |
| A phase to ground fault occurring on busbar M fault, resistance of 200 Ω | | | | | | | | |
| The number of sample points being dropped at random | 2 | | 4 | | 6 | | Not data lost | |
| N-th sampling point after failure | Virtual current(kA) | Reference current(kA) | Virtual current(kA) | Reference current(kA) | Virtual current(kA) | Reference current(kA) | Virtual current(kA) | Reference current(kA) |
| 1 | -2.9766 | -0.758 | -2.9766 | -0.758 | -2.9766 | -0.758 | -2.9766 | -0.758 |
| 2 | -2.9861 | -0.7636 | -2.9861 | 0 | -2.9861 | -0.7636 | -2.9861 | -0.7636 |
| 3 | -2.9939 | -0.7685 | -2.9939 | 0 | -2.9939 | 0 | -2.9939 | -0.7685 |
| 4 | -2.9991 | -0.7725 | -2.9991 | -0.7725 | -2.9991 | -0.7725 | -2.9991 | -0.7725 |
| 5 | -3.0053 | -0.7769 | -3.0053 | -0.7769 | -3.0053 | 0 | -3.0053 | -0.7769 |
| 6 | -3.0129 | -0.7821 | -3.0129 | -0.7821 | -3.0129 | -0.7821 | -3.0129 | -0.7821 |
| 7 | -3.0199 | -0.7872 | -3.0199 | -0.7872 | -3.0199 | -0.7872 | -3.0199 | -0.7872 |
| 8 | -3.0271 | 0 | -3.0271 | -0.7925 | -3.0271 | -0.7925 | -3.0271 | -0.7925 |
| 9 | -3.033 | -0.7973 | -3.033 | -0.7973 | -3.033 | -0.7973 | -3.033 | -0.7973 |
| 10 | -3.0373 | -0.8016 | -3.0373 | 0 | -3.0373 | -0.8016 | -3.0373 | -0.8016 |
| 11 | -3.0419 | -0.8062 | -3.0419 | -0.8062 | -3.0419 | 0 | -3.0419 | -0.8062 |
| 12 | -3.0462 | -0.8107 | -3.0462 | -0.8107 | -3.0462 | -0.8107 | -3.0462 | -0.8107 |
| 13 | -3.0471 | 0 | -3.0471 | -0.814 | -3.0471 | -0.814 | -3.0471 | -0.814 |
| 14 | -3.0445 | -0.816 | -3.0445 | -0.816 | -3.0445 | 0 | -3.0445 | -0.816 |
| 15 | -3.0397 | -0.8172 | -3.0397 | -0.8172 | -3.0397 | 0 | -3.0397 | -0.8172 |
| 16 | -3.0318 | -0.8173 | -3.0318 | -0.8173 | -3.0318 | -0.8173 | -3.0318 | -0.8173 |
| 17 | -3.0233 | -0.8173 | -3.0233 | -0.8173 | -3.0233 | -0.8173 | -3.0233 | -0.8173 |
| 18 | -3.0142 | -0.8172 | -3.0142 | -0.8172 | -3.0142 | -0.8172 | -3.0142 | -0.8172 |
| 19 | -3.0028 | -0.8163 | -3.0028 | 0 | -3.0028 | -0.8163 | -3.0028 | -0.8163 |
| 20 | -2.9894 | -0.8147 | -2.9894 | -0.8147 | -2.9894 | 0 | -2.9894 | -0.8147 |
| *θ* | 0.33 | | 0.47 | | 0.59 | | 0.02 | |
| AB phase to ground fault occurring on busbar M, fault resistance of 300 Ω | | | | | | | | |
| The number of sample points being dropped at random | 2 | | 4 | | 6 | | Not data lost | |
| N-th sampling point after failure | Virtual current(kA) | Reference current(kA) | Virtual current(kA) | Reference current(kA) | Virtual current(kA) | Reference current(kA) | Virtual current(kA) | Reference current(kA) |
| 1 | 0.6854 | 0.2092 | 0.6854 | 0.2092 | 0.6854 | 0.2092 | 0.6854 | 0.2092 |
| 2 | 0 | 0.2402 | 0.7947 | 0.2402 | 0 | 0.2402 | 0.7947 | 0.2402 |
| 3 | 0.901 | 0.2699 | 0.901 | 0.2699 | 0.901 | 0.2699 | 0.901 | 0.2699 |
| 4 | 1.0036 | 0.2981 | 1.0036 | 0.2981 | 1.0036 | 0.2981 | 1.0036 | 0.2981 |
| 5 | 1.1053 | 0.3257 | 1.1053 | 0.3257 | 1.1053 | 0.3257 | 1.1053 | 0.3257 |
| 6 | 1.2047 | 0.3523 | 0 | 0.3523 | 0 | 0.3523 | 1.2047 | 0.3523 |
| 7 | 1.3041 | 0.3787 | 1.3041 | 0.3787 | 1.3041 | 0.3787 | 1.3041 | 0.3787 |
| 8 | 1.4038 | 0.4052 | 1.4038 | 0.4052 | 1.4038 | 0.4052 | 1.4038 | 0.4052 |
| 9 | 1.5011 | 0.4305 | 1.5011 | 0.4305 | 1.5011 | 0.4305 | 1.5011 | 0.4305 |
| 10 | 0 | 0.455 | 1.5963 | 0.455 | 1.5963 | 0.455 | 1.5963 | 0.455 |
| 11 | 1.6941 | 0.4802 | 1.6941 | 0.4802 | 0 | 0.4802 | 1.6941 | 0.4802 |
| 12 | 1.7946 | 0.5063 | 1.7946 | 0.5063 | 1.7946 | 0.5063 | 1.7946 | 0.5063 |
| 13 | 1.8955 | 0.5325 | 0 | 0.5325 | 0 | 0.5325 | 1.8955 | 0.5325 |
| 14 | 1.9982 | 0.5591 | 1.9982 | 0.5591 | 1.9982 | 0.5591 | 1.9982 | 0.5591 |
| 15 | 2.099 | 0.5849 | 2.099 | 0.5849 | 2.099 | 0.5849 | 2.099 | 0.5849 |
| 16 | 2.1977 | 0.6097 | 0 | 0.6097 | 0 | 0.6097 | 2.1977 | 0.6097 |
| 17 | 2.2968 | 0.6344 | 2.2968 | 0.6344 | 2.2968 | 0.6344 | 2.2968 | 0.6344 |
| 18 | 2.3956 | 0.6589 | 0 | 0.6589 | 0 | 0.6589 | 2.3956 | 0.6589 |
| 19 | 2.4967 | 0.6842 | 2.4967 | 0.6842 | 2.4967 | 0.6842 | 2.4967 | 0.6842 |
| 20 | 2.6007 | 0.7105 | 2.6007 | 0.7105 | 2.6007 | 0.7105 | 2.6007 | 0.7105 |
| *θ* | 0.48 | | 0.68 | | 0.83 | | 0.03 | |
