# Supplementary material for: New principle of busbar protection based on a fundamental frequency polarity comparison
Source: PLoS One. 2019 Mar 21;14(3):e0213308. doi: 10.1371/journal.pone.0213308 (PMC6428346; doi:10.1371/journal.pone.0213308)
Supplement: S9 Table — (DOCX) [file pone.0213308.s010.docx]

| **S9 Table. Test Results of the Protection Algorithm When a Number of Sample Points are Randomly Lost for External Busbar Faults** | | | | | | | | |
| --- | --- | --- | --- | --- | --- | --- | --- | --- |
| A fault occurring at point BC on transmission line L_2_ at a distance of 20 km from busbar M | | | | | | | | |
| The number of sample points being dropped at random | 2 | | 4 | | 6 | | Not data lost | |
| N-th sampling point after failure | Virtual current(kA) | Reference current(kA) | Virtual current(kA) | Reference current(kA) | Virtual current(kA) | Reference current(kA) | Virtual current(kA) | Reference current(kA) |
| 1 | 1.0918 | -1.0924 | 1.0918 | -1.0924 | -2.9766 | -0.758 | 1.0918 | -1.0924 |
| 2 | 1.1013 | -1.1019 | 1.1013 | -1.1019 | -2.9861 | -0.7636 | 1.1013 | -1.1019 |
| 3 | 1.1101 | -1.1106 | 1.1101 | -1.1106 | -2.9939 | 0 | 1.1101 | -1.1106 |
| 4 | 1.1178 | 0 | 1.1178 | -1.1182 | -2.9991 | -0.7725 | 1.1178 | -1.1182 |
| 5 | 1.1261 | -1.1265 | 1.1261 | -1.1265 | -3.0053 | 0 | 1.1261 | -1.1265 |
| 6 | 1.1353 | -1.1356 | 1.1353 | -1.1356 | -3.0129 | -0.7821 | 1.1353 | -1.1356 |
| 7 | 1.1443 | -1.1446 | 1.1443 | 0 | -3.0199 | -0.7872 | 1.1443 | -1.1446 |
| 8 | 1.1537 | -1.1538 | 1.1537 | -1.1538 | -3.0271 | -0.7925 | 1.1537 | -1.1538 |
| 9 | 1.1626 | -1.1626 | 1.1626 | -1.1626 | -3.033 | -0.7973 | 1.1626 | -1.1626 |
| 10 | 1.1708 | -1.1708 | 1.1708 | 0 | -3.0373 | -0.8016 | 1.1708 | -1.1708 |
| 11 | 1.1794 | -1.1793 | 1.1794 | -1.1793 | -3.0419 | 0 | 1.1794 | -1.1793 |
| 12 | 1.188 | -1.1879 | 1.188 | -1.1879 | -3.0462 | -0.8107 | 1.188 | -1.1879 |
| 13 | 1.1951 | -1.195 | 1.1951 | 0 | -3.0471 | -0.814 | 1.1951 | -1.195 |
| 14 | 1.2008 | -1.2005 | 1.2008 | -1.2005 | -3.0445 | 0 | 1.2008 | -1.2005 |
| 15 | 1.2056 | -1.2053 | 1.2056 | -1.2053 | -3.0397 | 0 | 1.2056 | -1.2053 |
| 16 | 1.2091 | -1.2088 | 1.2091 | -1.2088 | -3.0318 | -0.8173 | 1.2091 | -1.2088 |
| 17 | 1.2125 | -1.2121 | 1.2125 | -1.2121 | -3.0233 | -0.8173 | 1.2125 | -1.2121 |
| 18 | 1.2158 | -1.2154 | 1.2158 | 0 | -3.0142 | -0.8172 | 1.2158 | -1.2154 |
| 19 | 1.2182 | 0 | 1.2182 | -1.2177 | -3.0028 | -0.8163 | 1.2182 | -1.2177 |
| 20 | 1.2198 | -1.2194 | 1.2198 | -1.2194 | -2.9894 | 0 | 1.2198 | -1.2194 |
| *θ* | 0.33 | | 0.47 | | 0.59 | | 0.02 | |
| A phase to ground fault occurring on transmission line L_4_ at a distance of 120 km from busbar M | | | | | | | | |
| The number of sample points being dropped at random | 2 | | 4 | | 6 | | Not data lost | |
| N-th sampling point after failure | Virtual current(kA) | Reference current(kA) | Virtual current(kA) | Reference current(kA) | Virtual current(kA) | Reference current(kA) | Virtual current(kA) | Reference current(kA) |
| 1 | 0.423 | -0.4226 | 0.423 | -0.4226 | 0.423 | -0.4226 | 0.423 | -0.4226 |
| 2 | 0.4249 | -0.4244 | 0 | -0.4244 | 0.4249 | -0.4244 | 0.4249 | -0.4244 |
| 3 | 0.4264 | -0.4258 | 0.4264 | -0.4258 | 0.4264 | -0.4258 | 0.4264 | -0.4258 |
| 4 | 0 | -0.4269 | 0.4275 | -0.4269 | 0.4275 | -0.4269 | 0.4275 | -0.4269 |
| 5 | 0.4289 | -0.4283 | 0.4289 | -0.4283 | 0 | -0.4283 | 0.4289 | -0.4283 |
| 6 | 0.4307 | -0.43 | 0 | -0.43 | 0.4307 | -0.43 | 0.4307 | -0.43 |
| 7 | 0.4326 | -0.4319 | 0.4326 | -0.4319 | 0.4326 | -0.4319 | 0.4326 | -0.4319 |
| 8 | 0.4348 | -0.434 | 0.4348 | -0.434 | 0 | -0.434 | 0.4348 | -0.434 |
| 9 | 0.4367 | -0.4359 | 0.4367 | -0.4359 | 0.4367 | -0.4359 | 0.4367 | -0.4359 |
| 10 | 0.4385 | -0.4376 | 0.4385 | -0.4376 | 0 | -0.4376 | 0.4385 | -0.4376 |
| 11 | 0.4405 | -0.4395 | 0.4405 | -0.4395 | 0.4405 | -0.4395 | 0.4405 | -0.4395 |
| 12 | 0.4425 | -0.4415 | 0.4425 | -0.4415 | 0.4425 | -0.4415 | 0.4425 | -0.4415 |
| 13 | 0.4443 | -0.4433 | 0.4443 | -0.4433 | 0 | -0.4433 | 0.4443 | -0.4433 |
| 14 | 0.446 | -0.4449 | 0 | -0.4449 | 0.446 | -0.4449 | 0.446 | -0.4449 |
| 15 | 0.4475 | -0.4464 | 0.4475 | -0.4464 | 0.4475 | -0.4464 | 0.4475 | -0.4464 |
| 16 | 0.4488 | -0.4477 | 0.4488 | -0.4477 | 0 | -0.4477 | 0.4488 | -0.4477 |
| 17 | 0.4503 | -0.4491 | 0.4503 | -0.4491 | 0.4503 | -0.4491 | 0.4503 | -0.4491 |
| 18 | 0.452 | -0.4507 | 0.452 | -0.4507 | 0.452 | -0.4507 | 0.452 | -0.4507 |
| 19 | 0 | -0.4522 | 0.4535 | -0.4522 | 0 | -0.4522 | 0.4535 | -0.4522 |
| 20 | 0.4551 | -0.4537 | 0 | -0.4537 | 0.4551 | -0.4537 | 0.4551 | -0.4537 |
| *θ* | 2.81 | | 2.66 | | 2.54 | | 3.14 | |
