# Supplementary material for: New principle of busbar protection based on a fundamental frequency polarity comparison
Source: PLoS One. 2019 Mar 21;14(3):e0213308. doi: 10.1371/journal.pone.0213308 (PMC6428346; doi:10.1371/journal.pone.0213308)
Supplement: S10 Table — (DOCX) [file pone.0213308.s011.docx]

| **S10 Table. Test Results of the Protection Algorithm under Different Noise Conditions for Internal and External Busbar Faults.** | | | | | | | | |
| --- | --- | --- | --- | --- | --- | --- | --- | --- |
| A phase to ground fault occurring at on busbar M, fault resistance of 200 Ω (F_3_) (fault initial angle of 45°) | | | | | | | | |
| SNR/dB | 40 | | 30 | | 20 | | 10 | |
| N-th sampling point after failure | Virtual current(kA) | Reference current(kA) | Virtual current(kA) | Reference current(kA) | Virtual current(kA) | Reference current(kA) | Virtual current(kA) | Reference current(kA) |
| 1 | -2.9759 | -0.7576 | -2.9776 | -0.7549 | -2.9637 | -0.7463 | -2.9821 | -0.7617 |
| 2 | -2.9855 | -0.7632 | -2.9872 | -0.7604 | -2.9732 | -0.7517 | -2.9917 | -0.7673 |
| 3 | -2.9932 | -0.7681 | -2.995 | -0.7653 | -2.9808 | -0.7565 | -2.9995 | -0.7722 |
| 4 | -2.9984 | -0.772 | -3.0003 | -0.7693 | -2.986 | -0.7604 | -3.0048 | -0.7762 |
| 5 | -3.0046 | -0.7765 | -3.0065 | -0.7738 | -2.9921 | -0.7648 | -3.011 | -0.7807 |
| 6 | -3.0122 | -0.7817 | -3.0142 | -0.779 | -2.9997 | -0.7699 | -3.0187 | -0.7859 |
| 7 | -3.0191 | -0.7867 | -3.0212 | -0.784 | -3.0065 | -0.7749 | -3.0256 | -0.7909 |
| 8 | -3.0263 | -0.792 | -3.0284 | -0.7893 | -3.0136 | -0.7801 | -3.0329 | -0.7962 |
| 9 | -3.0322 | -0.7969 | -3.0344 | -0.7942 | -3.0194 | -0.7848 | -3.0388 | -0.8011 |
| 10 | -3.0366 | -0.8012 | -3.0388 | -0.7985 | -3.0237 | -0.7891 | -3.0432 | -0.8054 |
| 11 | -3.0411 | -0.8057 | -3.0434 | -0.803 | -3.0282 | -0.7935 | -3.0478 | -0.81 |
| 12 | -3.0455 | -0.8103 | -3.0478 | -0.8076 | -3.0325 | -0.798 | -3.0522 | -0.8145 |
| 13 | -3.0464 | -0.8136 | -3.0488 | -0.8109 | -3.0333 | -0.8012 | -3.0531 | -0.8178 |
| 14 | -3.0437 | -0.8155 | -3.0461 | -0.8129 | -3.0307 | -0.8032 | -3.0505 | -0.8198 |
| 15 | -3.0389 | -0.8168 | -3.0413 | -0.8141 | -3.0258 | -0.8044 | -3.0458 | -0.821 |
| 16 | -3.031 | -0.8169 | -3.0335 | -0.8143 | -3.0179 | -0.8045 | -3.038 | -0.8212 |
| 17 | -3.0225 | -0.8168 | -3.025 | -0.8143 | -3.0094 | -0.8045 | -3.0296 | -0.8211 |
| 18 | -3.0135 | -0.8167 | -3.016 | -0.8142 | -3.0004 | -0.8044 | -3.0207 | -0.821 |
| 19 | -3.002 | -0.8158 | -3.0045 | -0.8133 | -2.989 | -0.8035 | -3.0093 | -0.8201 |
| 20 | -2.9887 | -0.8142 | -2.9912 | -0.8117 | -2.9756 | -0.802 | -2.9961 | -0.8185 |
| *θ* | 0.21 | | 0.21 | | 0.21 | | 0.21 | |
| AB phase to ground short circuit occurring on transmission line L_2_ at a distance of 80 km from busbar M, fault resistance of 100 Ω (fault initial angle of 90°) | | | | | | | | |
| SNR/dB | 40 | | 30 | | 20 | | 10 | |
| N-th sampling point after failure | Virtual current(kA) | Reference current(kA) | Virtual current(kA) | Reference current(kA) | Virtual current(kA) | Reference current(kA) | Virtual current(kA) | Reference current(kA) |
| 1 | -0.1021 | 0.1061 | -0.1025 | 0.1032 | -0.1074 | 0.1018 | -0.1255 | 0.1228 |
| 2 | -0.1065 | 0.1104 | -0.1068 | 0.1076 | -0.112 | 0.1061 | -0.1293 | 0.1266 |
| 3 | -0.1104 | 0.1143 | -0.1106 | 0.1116 | -0.1162 | 0.1101 | -0.1327 | 0.1299 |
| 4 | -0.1138 | 0.1177 | -0.114 | 0.115 | -0.1197 | 0.1135 | -0.1357 | 0.1328 |
| 5 | -0.1169 | 0.1209 | -0.1171 | 0.1182 | -0.1231 | 0.1167 | -0.1384 | 0.1355 |
| 6 | -0.1197 | 0.1236 | -0.1198 | 0.1211 | -0.126 | 0.1196 | -0.1407 | 0.1379 |
| 7 | -0.1224 | 0.1263 | -0.1224 | 0.1238 | -0.1289 | 0.1223 | -0.143 | 0.1402 |
| 8 | -0.125 | 0.1289 | -0.125 | 0.1265 | -0.1317 | 0.125 | -0.1453 | 0.1424 |
| 9 | -0.1272 | 0.1312 | -0.1271 | 0.1288 | -0.1341 | 0.1273 | -0.1471 | 0.1442 |
| 10 | -0.129 | 0.1331 | -0.129 | 0.1307 | -0.1361 | 0.1293 | -0.1487 | 0.1458 |
| 11 | -0.1311 | 0.1352 | -0.131 | 0.1328 | -0.1383 | 0.1314 | -0.1504 | 0.1476 |
| 12 | -0.1333 | 0.1374 | -0.1331 | 0.1351 | -0.1407 | 0.1337 | -0.1522 | 0.1494 |
| 13 | -0.1354 | 0.1395 | -0.1352 | 0.1373 | -0.143 | 0.136 | -0.154 | 0.1512 |
| 14 | -0.1376 | 0.1418 | -0.1374 | 0.1396 | -0.1454 | 0.1383 | -0.1559 | 0.1531 |
| 15 | -0.1395 | 0.1437 | -0.1392 | 0.1415 | -0.1475 | 0.1403 | -0.1574 | 0.1547 |
| 16 | -0.141 | 0.1452 | -0.1407 | 0.1431 | -0.1491 | 0.1419 | -0.1586 | 0.1559 |
| 17 | -0.1424 | 0.1467 | -0.1421 | 0.1446 | -0.1507 | 0.1435 | -0.1597 | 0.1571 |
| 18 | -0.1436 | 0.148 | -0.1434 | 0.1459 | -0.1522 | 0.1449 | -0.1607 | 0.1582 |
| 19 | -0.1451 | 0.1495 | -0.1448 | 0.1475 | -0.1538 | 0.1465 | -0.1618 | 0.1594 |
| 20 | -0.1467 | 0.1512 | -0.1464 | 0.1492 | -0.1556 | 0.1483 | -0.1632 | 0.1608 |
| *θ* | 3.14 | | 3.14 | | 3.14 | | 3.14 | |
