# Supplementary material for: New principle of busbar protection based on a fundamental frequency polarity comparison
Source: PLoS One. 2019 Mar 21;14(3):e0213308. doi: 10.1371/journal.pone.0213308 (PMC6428346; doi:10.1371/journal.pone.0213308)
Supplement: S11 Table — (DOCX) [file pone.0213308.s012.docx]

**S11 Table. The data obtained from Fig 3 and Fig 4 is as follows**

| A phase to ground fault (F_1_) occurring on busbar M (fault initial angle of 45°) | | |
| --- | --- | --- |
| The Fault Resistances | 200Ω | |
| N-th sampling point after failure | Virtual current(kA) | Reference current(kA) |
| 1 | -2.9766 | -0.758 |
| 2 | -2.9861 | -0.7636 |
| 3 | -2.9939 | -0.7685 |
| 4 | -2.9991 | -0.7725 |
| 5 | -3.0053 | -0.7769 |
| 6 | -3.0129 | -0.7821 |
| 7 | -3.0199 | -0.7872 |
| 8 | -3.0271 | -0.7925 |
| 9 | -3.033 | -0.7973 |
| 10 | -3.0373 | -0.8016 |
| 11 | -3.0419 | -0.8062 |
| 12 | -3.0462 | -0.8107 |
| 13 | -3.0471 | -0.814 |
| 14 | -3.0445 | -0.816 |
| 15 | -3.0397 | -0.8172 |
| 16 | -3.0318 | -0.8173 |
| 17 | -3.0233 | -0.8173 |
| 18 | -3.0142 | -0.8172 |
| 19 | -3.0028 | -0.8163 |
| 20 | -2.9894 | -0.8147 |
| *θ* | 0.021 | |
