# Supplementary material for: New principle of busbar protection based on a fundamental frequency polarity comparison
Source: PLoS One. 2019 Mar 21;14(3):e0213308. doi: 10.1371/journal.pone.0213308 (PMC6428346; doi:10.1371/journal.pone.0213308)
Supplement: S12 Table — (DOCX) [file pone.0213308.s013.docx]

**S12 Table. The data obtained from Fig.5 and Fig.6 is as follows**

| AB phase to ground fault occurring at F_3_ on transmission line L_~~4~~_ at a distance of 50 km from busbar M | | |
| --- | --- | --- |
| The fault initial angle | 90° | |
| N-th sampling point after failure | Virtual current(kA) | Reference current(kA) |
| 1 | -0.0812 | 0.0821 |
| 2 | -0.1017 | 0.1026 |
| 3 | -0.1213 | 0.1221 |
| 4 | -0.1396 | 0.1404 |
| 5 | -0.1575 | 0.1584 |
| 6 | -0.1746 | 0.1755 |
| 7 | -0.1915 | 0.1924 |
| 8 | -0.2084 | 0.2094 |
| 9 | -0.2245 | 0.2255 |
| 10 | -0.2398 | 0.2409 |
| 11 | -0.2558 | 0.2568 |
| 12 | -0.2723 | 0.2734 |
| 13 | -0.2888 | 0.2899 |
| 14 | -0.3056 | 0.3068 |
| 15 | -0.3218 | 0.323 |
| 16 | -0.3372 | 0.3385 |
| 17 | -0.3525 | 0.3539 |
| 18 | -0.3676 | 0.369 |
| 19 | -0.3833 | 0.3847 |
| 20 | -0.3997 | 0.4012 |
| *θ* | 3.14 | |
