# Supplementary material for: New principle of busbar protection based on a fundamental frequency polarity comparison
Source: PLoS One. 2019 Mar 21;14(3):e0213308. doi: 10.1371/journal.pone.0213308 (PMC6428346; doi:10.1371/journal.pone.0213308)
Supplement: S13 Table — (DOCX) [file pone.0213308.s014.docx]

**S13 Table. The data obtained from Fig.9 and Fig.10 is as follows**

| A phase to ground fault occurring at on busbar M, fault resistance of 200 Ω (F_3_) (fault initial angle of 45°) | | | AB phase to ground short circuit occurring on transmission line L_2_ at a distance of 80 km from busbar M, fault resistance of 100 Ω (fault initial angle of 90°) | |
| --- | --- | --- | --- | --- |
| SNR/dB | 10 | | 10 | |
| N-th sampling point after failure | Virtual current(kA) | Reference current(kA) | Virtual current(kA) | Reference current(kA) |
| 1 | -2.9821 | -0.7617 | -0.1255 | 0.1228 |
| 2 | -2.9917 | -0.7673 | -0.1293 | 0.1266 |
| 3 | -2.9995 | -0.7722 | -0.1327 | 0.1299 |
| 4 | -3.0048 | -0.7762 | -0.1357 | 0.1328 |
| 5 | -3.011 | -0.7807 | -0.1384 | 0.1355 |
| 6 | -3.0187 | -0.7859 | -0.1407 | 0.1379 |
| 7 | -3.0256 | -0.7909 | -0.143 | 0.1402 |
| 8 | -3.0329 | -0.7962 | -0.1453 | 0.1424 |
| 9 | -3.0388 | -0.8011 | -0.1471 | 0.1442 |
| 10 | -3.0432 | -0.8054 | -0.1487 | 0.1458 |
| 11 | -3.0478 | -0.81 | -0.1504 | 0.1476 |
| 12 | -3.0522 | -0.8145 | -0.1522 | 0.1494 |
| 13 | -3.0531 | -0.8178 | -0.154 | 0.1512 |
| 14 | -3.0505 | -0.8198 | -0.1559 | 0.1531 |
| 15 | -3.0458 | -0.821 | -0.1574 | 0.1547 |
| 16 | -3.038 | -0.8212 | -0.1586 | 0.1559 |
| 17 | -3.0296 | -0.8211 | -0.1597 | 0.1571 |
| 18 | -3.0207 | -0.821 | -0.1607 | 0.1582 |
| 19 | -3.0093 | -0.8201 | -0.1618 | 0.1594 |
| 20 | -2.9961 | -0.8185 | -0.1632 | 0.1608 |
| *θ* | 0.21 | | 3.14 | |
