# Supplementary material for: New principle of busbar protection based on a fundamental frequency polarity comparison
Source: PLoS One. 2019 Mar 21;14(3):e0213308. doi: 10.1371/journal.pone.0213308 (PMC6428346; doi:10.1371/journal.pone.0213308)
Supplement: S14 Table — (DOCX) [file pone.0213308.s015.docx]

**S14 Table. The data after wavelet transform of 400 sampling points in Fig.3, Fig.4, Fig.9, and Fig.10 are as follows.**

| Wavelet data after wavelet transform in Fig 3. | | | Wavelet data after wavelet transform in Fig 4. | | Wavelet data after wavelet transform in Fig 9. | | Wavelet data after wavelet transform in Fig 10. | |
| --- | --- | --- | --- | --- | --- | --- | --- | --- |
| N-th sampling point | Virtual current  (kA) | Reference current  (kA) | Virtual current  (kA) | Reference current  (kA) | Virtual current  (kA) | Reference current  (kA) | Virtual current  (kA) | Reference current  (kA) |
| 1 | 0.4575 | 0.2481 | -0.1785 | 0.1748 | 0.4553 | 0.2559 | -0.0877 | 0.1066 |
| 2 | 0.4604 | 0.2592 | -0.1878 | 0.1839 | 0.4581 | 0.2664 | -0.0956 | 0.1156 |
| 3 | 0.4633 | 0.2705 | -0.1973 | 0.1933 | 0.461 | 0.2771 | -0.1038 | 0.1247 |
| 4 | 0.4663 | 0.282 | -0.2071 | 0.2028 | 0.464 | 0.2882 | -0.112 | 0.1341 |
| 5 | 0.4694 | 0.2937 | -0.2169 | 0.2125 | 0.4669 | 0.2994 | -0.1204 | 0.1436 |
| 6 | 0.4724 | 0.3057 | -0.227 | 0.2223 | 0.4699 | 0.3108 | -0.129 | 0.1534 |
| 7 | 0.4754 | 0.3177 | -0.2371 | 0.2322 | 0.4729 | 0.3223 | -0.1376 | 0.1631 |
| 8 | 0.4784 | 0.3298 | -0.2473 | 0.2422 | 0.4758 | 0.3338 | -0.1462 | 0.173 |
| 9 | 0.4814 | 0.342 | -0.2576 | 0.2523 | 0.4788 | 0.3455 | -0.155 | 0.183 |
| 10 | 0.4844 | 0.3544 | -0.2681 | 0.2625 | 0.4817 | 0.3573 | -0.1639 | 0.1931 |
| 11 | 0.4861 | 0.3654 | -0.2775 | 0.2717 | 0.4833 | 0.3678 | -0.1721 | 0.2023 |
| 12 | 0.4862 | 0.3749 | -0.2856 | 0.2797 | 0.4834 | 0.3768 | -0.1794 | 0.2106 |
| 13 | 0.4855 | 0.3836 | -0.2931 | 0.287 | 0.4827 | 0.3851 | -0.1863 | 0.2183 |
| 14 | 0.4835 | 0.391 | -0.2996 | 0.2934 | 0.4806 | 0.3921 | -0.1925 | 0.2253 |
| 15 | 0.4811 | 0.398 | -0.3059 | 0.2995 | 0.4781 | 0.3988 | -0.1985 | 0.232 |
| 16 | 0.4781 | 0.4047 | -0.3118 | 0.3053 | 0.4751 | 0.4051 | -0.2043 | 0.2385 |
| 17 | 0.474 | 0.4102 | -0.3168 | 0.3102 | 0.471 | 0.4103 | -0.2095 | 0.2443 |
| 18 | 0.4688 | 0.4147 | -0.321 | 0.3143 | 0.4657 | 0.4145 | -0.2141 | 0.2495 |
| 19 | 0.4636 | 0.4191 | -0.3252 | 0.3184 | 0.4604 | 0.4187 | -0.2188 | 0.2546 |
| 20 | 0.4581 | 0.4236 | -0.3294 | 0.3224 | 0.4549 | 0.4229 | -0.2235 | 0.2598 |
| 21 | 0.4522 | 0.4275 | -0.3333 | 0.3262 | 0.4489 | 0.4266 | -0.2279 | 0.2647 |
| 22 | 0.4459 | 0.4313 | -0.337 | 0.3298 | 0.4426 | 0.4302 | -0.2323 | 0.2695 |
| 23 | 0.4385 | 0.434 | -0.3398 | 0.3325 | 0.4351 | 0.4327 | -0.2361 | 0.2736 |
| 24 | 0.43 | 0.4355 | -0.3417 | 0.3344 | 0.4265 | 0.4341 | -0.2392 | 0.277 |
| 25 | 0.4208 | 0.4365 | -0.3432 | 0.3358 | 0.4173 | 0.4349 | -0.2421 | 0.2801 |
| 26 | 0.4108 | 0.4367 | -0.3441 | 0.3366 | 0.4072 | 0.435 | -0.2445 | 0.2826 |
| 27 | 0.4009 | 0.4373 | -0.3453 | 0.3377 | 0.3973 | 0.4355 | -0.2472 | 0.2855 |
| 28 | 0.3913 | 0.4383 | -0.3468 | 0.3392 | 0.3876 | 0.4363 | -0.2502 | 0.2886 |
| 29 | 0.3814 | 0.4391 | -0.3482 | 0.3405 | 0.3777 | 0.437 | -0.2531 | 0.2917 |
| 30 | 0.3714 | 0.44 | -0.3497 | 0.342 | 0.3676 | 0.4378 | -0.256 | 0.295 |
| 31 | 0.361 | 0.4406 | -0.3509 | 0.3431 | 0.3571 | 0.4383 | -0.2588 | 0.2979 |
| 32 | 0.35 | 0.4406 | -0.3517 | 0.3439 | 0.3461 | 0.4383 | -0.2614 | 0.3007 |
| 33 | 0.3389 | 0.4408 | -0.3527 | 0.3448 | 0.3349 | 0.4383 | -0.264 | 0.3035 |
| 34 | 0.3277 | 0.4409 | -0.3536 | 0.3456 | 0.3236 | 0.4384 | -0.2666 | 0.3063 |
| 35 | 0.3153 | 0.44 | -0.3536 | 0.3456 | 0.3112 | 0.4374 | -0.2687 | 0.3085 |
| 36 | 0.3018 | 0.438 | -0.3528 | 0.3448 | 0.2977 | 0.4354 | -0.2702 | 0.31 |
| 37 | 0.2877 | 0.4353 | -0.3515 | 0.3435 | 0.2834 | 0.4328 | -0.2713 | 0.3111 |
| 38 | 0.2725 | 0.4318 | -0.3495 | 0.3415 | 0.2682 | 0.4292 | -0.272 | 0.3116 |
| 39 | 0.257 | 0.428 | -0.3474 | 0.3393 | 0.2527 | 0.4255 | -0.2726 | 0.312 |
| 40 | 0.2413 | 0.4241 | -0.3451 | 0.3371 | 0.2368 | 0.4217 | -0.2731 | 0.3124 |
| 41 | 0.2247 | 0.4195 | -0.3423 | 0.3342 | 0.2202 | 0.4172 | -0.2733 | 0.3124 |
| 42 | 0.2075 | 0.4143 | -0.339 | 0.331 | 0.2029 | 0.4121 | -0.2731 | 0.312 |
| 43 | 0.1906 | 0.4096 | -0.3362 | 0.3281 | 0.186 | 0.4075 | -0.2733 | 0.3119 |
| 44 | 0.1741 | 0.4054 | -0.3338 | 0.3257 | 0.1694 | 0.4034 | -0.2738 | 0.3123 |
| 45 | 0.1576 | 0.4014 | -0.3315 | 0.3234 | 0.1529 | 0.3995 | -0.2745 | 0.3127 |
| 46 | 0.1414 | 0.3979 | -0.3296 | 0.3215 | 0.1366 | 0.3959 | -0.2754 | 0.3135 |
| 47 | 0.1247 | 0.394 | -0.3274 | 0.3193 | 0.1198 | 0.3921 | -0.2762 | 0.3141 |
| 48 | 0.1076 | 0.3897 | -0.3249 | 0.3169 | 0.1026 | 0.3879 | -0.2768 | 0.3145 |
| 49 | 0.0906 | 0.3858 | -0.3227 | 0.3147 | 0.0856 | 0.384 | -0.2776 | 0.3151 |
| 50 | 0.0737 | 0.3819 | -0.3207 | 0.3126 | 0.0685 | 0.3802 | -0.2785 | 0.3159 |
| 51 | 0.0562 | 0.3777 | -0.3183 | 0.3102 | 0.0509 | 0.376 | -0.2792 | 0.3164 |
| 52 | 0.0382 | 0.3731 | -0.3156 | 0.3075 | 0.0328 | 0.3714 | -0.2797 | 0.3167 |
| 53 | 0.0198 | 0.3682 | -0.3127 | 0.3046 | 0.0144 | 0.3667 | -0.2802 | 0.3169 |
| 54 | 0.001 | 0.3631 | -0.3096 | 0.3015 | -0.0045 | 0.3616 | -0.2804 | 0.3169 |
| 55 | -0.0177 | 0.3581 | -0.3067 | 0.2986 | -0.0233 | 0.3567 | -0.2809 | 0.317 |
| 56 | -0.0364 | 0.3533 | -0.3039 | 0.2958 | -0.0421 | 0.3519 | -0.2814 | 0.3173 |
| 57 | -0.0554 | 0.3484 | -0.301 | 0.293 | -0.0612 | 0.3471 | -0.282 | 0.3176 |
| 58 | -0.0745 | 0.3435 | -0.2982 | 0.2901 | -0.0804 | 0.3422 | -0.2825 | 0.3179 |
| 59 | -0.0946 | 0.3376 | -0.2946 | 0.2865 | -0.1006 | 0.3364 | -0.2826 | 0.3176 |
| 60 | -0.1159 | 0.3307 | -0.2901 | 0.2821 | -0.122 | 0.3296 | -0.282 | 0.3166 |
| 61 | -0.1378 | 0.3232 | -0.2852 | 0.2772 | -0.1441 | 0.3223 | -0.2812 | 0.3154 |
| 62 | -0.1607 | 0.3148 | -0.2797 | 0.2717 | -0.167 | 0.3141 | -0.28 | 0.3136 |
| 63 | -0.184 | 0.3062 | -0.2739 | 0.2659 | -0.1904 | 0.3056 | -0.2786 | 0.3116 |
| 64 | -0.2077 | 0.2972 | -0.2678 | 0.2599 | -0.2143 | 0.2968 | -0.277 | 0.3094 |
| 65 | -0.2323 | 0.2873 | -0.2611 | 0.2533 | -0.239 | 0.2873 | -0.275 | 0.3067 |
| 66 | -0.2578 | 0.2768 | -0.2539 | 0.2461 | -0.2645 | 0.277 | -0.2727 | 0.3037 |
| 67 | -0.2833 | 0.2664 | -0.2467 | 0.2391 | -0.2901 | 0.2669 | -0.2704 | 0.3007 |
| 68 | -0.3088 | 0.2561 | -0.2396 | 0.2321 | -0.3158 | 0.2568 | -0.2683 | 0.2978 |
| 69 | -0.3347 | 0.2455 | -0.2324 | 0.2249 | -0.3418 | 0.2465 | -0.266 | 0.2947 |
| 70 | -0.3608 | 0.2349 | -0.2251 | 0.2177 | -0.368 | 0.2361 | -0.2637 | 0.2917 |
| 71 | -0.3876 | 0.2236 | -0.2174 | 0.21 | -0.395 | 0.2251 | -0.2611 | 0.2883 |
| 72 | -0.4153 | 0.2116 | -0.209 | 0.2018 | -0.4227 | 0.2135 | -0.2582 | 0.2845 |
| 73 | -0.4433 | 0.1994 | -0.2005 | 0.1933 | -0.4509 | 0.2016 | -0.2551 | 0.2805 |
| 74 | -0.4718 | 0.1869 | -0.1918 | 0.1847 | -0.4795 | 0.1893 | -0.2519 | 0.2764 |
| 75 | -0.4997 | 0.175 | -0.1836 | 0.1766 | -0.5076 | 0.1778 | -0.2491 | 0.2728 |
| 76 | -0.5271 | 0.1641 | -0.1761 | 0.1692 | -0.535 | 0.1672 | -0.2469 | 0.2698 |
| 77 | -0.5543 | 0.1535 | -0.1689 | 0.1621 | -0.5624 | 0.1568 | -0.2448 | 0.2671 |
| 78 | -0.5812 | 0.1435 | -0.1622 | 0.1554 | -0.5894 | 0.1471 | -0.2432 | 0.2649 |
| 79 | -0.6081 | 0.1336 | -0.1556 | 0.1489 | -0.6164 | 0.1374 | -0.2416 | 0.2627 |
| 80 | -0.6352 | 0.1237 | -0.1491 | 0.1424 | -0.6436 | 0.1278 | -0.2401 | 0.2606 |
| 81 | -0.6619 | 0.1145 | -0.143 | 0.1364 | -0.6704 | 0.1187 | -0.2389 | 0.259 |
| 82 | -0.6882 | 0.1058 | -0.1374 | 0.1308 | -0.6969 | 0.1102 | -0.2382 | 0.2578 |
| 83 | -0.7151 | 0.0967 | -0.1315 | 0.1249 | -0.7239 | 0.1013 | -0.2371 | 0.2563 |
| 84 | -0.7426 | 0.0871 | -0.1252 | 0.1187 | -0.7515 | 0.092 | -0.2359 | 0.2546 |
| 85 | -0.7703 | 0.0775 | -0.1189 | 0.1124 | -0.7793 | 0.0826 | -0.2347 | 0.2529 |
| 86 | -0.7983 | 0.0677 | -0.1124 | 0.106 | -0.8075 | 0.073 | -0.2334 | 0.2511 |
| 87 | -0.8261 | 0.0584 | -0.1064 | 0.1 | -0.8353 | 0.064 | -0.2324 | 0.2497 |
| 88 | -0.8535 | 0.0496 | -0.1008 | 0.0945 | -0.8629 | 0.0554 | -0.2318 | 0.2488 |
| 89 | -0.8811 | 0.0409 | -0.0952 | 0.089 | -0.8906 | 0.0469 | -0.2312 | 0.2478 |
| 90 | -0.9086 | 0.0324 | -0.0899 | 0.0836 | -0.9183 | 0.0386 | -0.2308 | 0.2471 |
| 91 | -0.9367 | 0.0235 | -0.0842 | 0.078 | -0.9465 | 0.0298 | -0.2302 | 0.2461 |
| 92 | -0.9654 | 0.014 | -0.0781 | 0.0719 | -0.9753 | 0.0206 | -0.2293 | 0.2449 |
| 93 | -0.9944 | 0.0045 | -0.0719 | 0.0658 | -1.0044 | 0.0113 | -0.2284 | 0.2436 |
| 94 | -1.0238 | -0.0053 | -0.0655 | 0.0595 | -1.0339 | 0.0017 | -0.2274 | 0.2421 |
| 95 | -1.0534 | -0.0152 | -0.0592 | 0.0532 | -1.0637 | -0.0079 | -0.2264 | 0.2407 |
| 96 | -1.0832 | -0.0251 | -0.0528 | 0.0468 | -1.0936 | -0.0176 | -0.2254 | 0.2393 |
| 97 | -1.1134 | -0.0352 | -0.0462 | 0.0403 | -1.1239 | -0.0275 | -0.2243 | 0.2378 |
| 98 | -1.144 | -0.0456 | -0.0394 | 0.0335 | -1.1546 | -0.0376 | -0.2231 | 0.2361 |
| 99 | -1.1744 | -0.0556 | -0.0329 | 0.0272 | -1.1852 | -0.0474 | -0.2222 | 0.2348 |
| 100 | -1.2046 | -0.0652 | -0.0269 | 0.0211 | -1.2155 | -0.0567 | -0.2215 | 0.2338 |
| 101 | -1.2348 | -0.0745 | -0.0209 | 0.0152 | -1.2459 | -0.0659 | -0.221 | 0.233 |
| 102 | -1.2649 | -0.0836 | -0.0153 | 0.0096 | -1.2761 | -0.0747 | -0.2207 | 0.2324 |
| 103 | -1.2953 | -0.0928 | -0.0095 | 0.0039 | -1.3066 | -0.0837 | -0.2203 | 0.2318 |
| 104 | -1.326 | -0.1022 | -0.0036 | -0.002 | -1.3375 | -0.0928 | -0.2199 | 0.2311 |
| 105 | -1.3567 | -0.1113 | 0.0021 | -0.0077 | -1.3684 | -0.1018 | -0.2197 | 0.2306 |
| 106 | -1.3876 | -0.1204 | 0.0077 | -0.0132 | -1.3993 | -0.1106 | -0.2195 | 0.2302 |
| 107 | -1.4194 | -0.1304 | 0.0141 | -0.0196 | -1.4313 | -0.1204 | -0.2188 | 0.2292 |
| 108 | -1.4523 | -0.1414 | 0.0212 | -0.0267 | -1.4644 | -0.1312 | -0.2176 | 0.2276 |
| 109 | -1.486 | -0.153 | 0.0289 | -0.0342 | -1.4982 | -0.1425 | -0.2162 | 0.2257 |
| 110 | -1.5206 | -0.1655 | 0.0372 | -0.0424 | -1.533 | -0.1547 | -0.2142 | 0.2232 |
| 111 | -1.5556 | -0.1782 | 0.0456 | -0.0508 | -1.5681 | -0.167 | -0.2122 | 0.2206 |
| 112 | -1.5909 | -0.1911 | 0.0542 | -0.0593 | -1.6035 | -0.1795 | -0.2101 | 0.218 |
| 113 | -1.6271 | -0.2047 | 0.0634 | -0.0684 | -1.6399 | -0.1928 | -0.2076 | 0.2148 |
| 114 | -1.664 | -0.219 | 0.073 | -0.0779 | -1.677 | -0.2067 | -0.2048 | 0.2114 |
| 115 | -1.7012 | -0.2333 | 0.0827 | -0.0875 | -1.7143 | -0.2206 | -0.2021 | 0.2079 |
| 116 | -1.7386 | -0.2476 | 0.0924 | -0.0971 | -1.7518 | -0.2346 | -0.1993 | 0.2045 |
| 117 | -1.7764 | -0.2623 | 0.1023 | -0.1069 | -1.7898 | -0.2488 | -0.1964 | 0.2009 |
| 118 | -1.8145 | -0.277 | 0.1123 | -0.1168 | -1.828 | -0.2631 | -0.1935 | 0.1973 |
| 119 | -1.8534 | -0.2925 | 0.1229 | -0.1272 | -1.8671 | -0.2782 | -0.1903 | 0.1932 |
| 120 | -1.8932 | -0.3086 | 0.134 | -0.1382 | -1.907 | -0.2939 | -0.1866 | 0.1888 |
| 121 | -1.9334 | -0.3252 | 0.1453 | -0.1494 | -1.9474 | -0.3099 | -0.1828 | 0.1841 |
| 122 | -1.9743 | -0.3421 | 0.1571 | -0.161 | -1.9885 | -0.3264 | -0.1788 | 0.1792 |
| 123 | -2.015 | -0.3588 | 0.1685 | -0.1723 | -2.0294 | -0.3426 | -0.175 | 0.1746 |
| 124 | -2.0555 | -0.3749 | 0.1795 | -0.1833 | -2.07 | -0.3583 | -0.1716 | 0.1703 |
| 125 | -2.0962 | -0.3911 | 0.1906 | -0.1942 | -2.1109 | -0.374 | -0.1681 | 0.1661 |
| 126 | -2.1369 | -0.4071 | 0.2014 | -0.2049 | -2.1517 | -0.3895 | -0.1648 | 0.1621 |
| 127 | -2.1779 | -0.4232 | 0.2124 | -0.2158 | -2.1929 | -0.4052 | -0.1615 | 0.158 |
| 128 | -2.2193 | -0.4395 | 0.2235 | -0.2268 | -2.2345 | -0.421 | -0.1581 | 0.1538 |
| 129 | -2.2608 | -0.4557 | 0.2345 | -0.2376 | -2.2761 | -0.4367 | -0.1548 | 0.1498 |
| 130 | -2.3023 | -0.4717 | 0.2453 | -0.2483 | -2.3178 | -0.4523 | -0.1516 | 0.146 |
| 131 | -2.3446 | -0.4883 | 0.2567 | -0.2595 | -2.3603 | -0.4684 | -0.1482 | 0.1417 |
| 132 | -2.3876 | -0.5056 | 0.2685 | -0.2712 | -2.4035 | -0.4852 | -0.1444 | 0.1371 |
| 133 | -2.4311 | -0.5232 | 0.2806 | -0.2832 | -2.4472 | -0.5023 | -0.1404 | 0.1323 |
| 134 | -2.4753 | -0.5413 | 0.293 | -0.2955 | -2.4915 | -0.5199 | -0.1363 | 0.1273 |
| 135 | -2.5196 | -0.5594 | 0.3054 | -0.3078 | -2.536 | -0.5374 | -0.1321 | 0.1223 |
| 136 | -2.5641 | -0.5774 | 0.3178 | -0.32 | -2.5806 | -0.5549 | -0.1281 | 0.1174 |
| 137 | -2.6091 | -0.5957 | 0.3304 | -0.3324 | -2.6258 | -0.5727 | -0.1239 | 0.1124 |
| 138 | -2.6544 | -0.6143 | 0.3431 | -0.345 | -2.6713 | -0.5907 | -0.1196 | 0.1072 |
| 139 | -2.6953 | -0.6311 | 0.3541 | -0.3559 | -2.7124 | -0.6071 | -0.1159 | 0.1027 |
| 140 | -2.7313 | -0.6462 | 0.3632 | -0.3649 | -2.7485 | -0.6217 | -0.1127 | 0.0987 |
| 141 | -2.7645 | -0.6603 | 0.3712 | -0.3727 | -2.7818 | -0.6354 | -0.1097 | 0.0951 |
| 142 | -2.7936 | -0.6728 | 0.3776 | -0.379 | -2.811 | -0.6475 | -0.1073 | 0.0921 |
| 143 | -2.8214 | -0.6849 | 0.3835 | -0.3848 | -2.839 | -0.6593 | -0.105 | 0.0891 |
| 144 | -2.848 | -0.6966 | 0.389 | -0.3902 | -2.8657 | -0.6707 | -0.1027 | 0.0863 |
| 145 | -2.8708 | -0.707 | 0.3929 | -0.394 | -2.8886 | -0.6808 | -0.1009 | 0.0839 |
| 146 | -2.8901 | -0.7161 | 0.3956 | -0.3966 | -2.908 | -0.6896 | -0.0994 | 0.0819 |
| 147 | -2.9094 | -0.7253 | 0.3982 | -0.3991 | -2.9275 | -0.6985 | -0.0979 | 0.08 |
| 148 | -2.9286 | -0.7343 | 0.4006 | -0.4015 | -2.9467 | -0.7073 | -0.0965 | 0.0781 |
| 149 | -2.9463 | -0.7428 | 0.4025 | -0.4033 | -2.9645 | -0.7156 | -0.0952 | 0.0764 |
| 150 | -2.9632 | -0.7511 | 0.4041 | -0.4048 | -2.9815 | -0.7236 | -0.094 | 0.0747 |
| 151 | -2.9766 | -0.758 | 0.4043 | -0.4049 | -2.9949 | -0.7303 | -0.0932 | 0.0736 |
| 152 | -2.9861 | -0.7636 | 0.403 | -0.4036 | -3.0046 | -0.7358 | -0.0928 | 0.0729 |
| 153 | -2.9939 | -0.7685 | 0.401 | -0.4015 | -3.0124 | -0.7405 | -0.0926 | 0.0724 |
| 154 | -2.9991 | -0.7725 | 0.3979 | -0.3984 | -3.0177 | -0.7444 | -0.0927 | 0.0723 |
| 155 | -3.0053 | -0.7769 | 0.3953 | -0.3957 | -3.0239 | -0.7487 | -0.0926 | 0.0719 |
| 156 | -3.0129 | -0.7821 | 0.3934 | -0.3937 | -3.0316 | -0.7538 | -0.0922 | 0.0712 |
| 157 | -3.0199 | -0.7872 | 0.3911 | -0.3915 | -3.0386 | -0.7587 | -0.0919 | 0.0705 |
| 158 | -3.0271 | -0.7925 | 0.3891 | -0.3893 | -3.0459 | -0.7638 | -0.0914 | 0.0697 |
| 159 | -3.033 | -0.7973 | 0.3865 | -0.3867 | -3.0518 | -0.7685 | -0.091 | 0.069 |
| 160 | -3.0373 | -0.8016 | 0.3834 | -0.3835 | -3.0562 | -0.7727 | -0.0908 | 0.0685 |
| 161 | -3.0419 | -0.8062 | 0.3804 | -0.3804 | -3.0608 | -0.7771 | -0.0905 | 0.0679 |
| 162 | -3.0462 | -0.8107 | 0.3773 | -0.3773 | -3.0652 | -0.7815 | -0.0902 | 0.0672 |
| 163 | -3.0471 | -0.814 | 0.3729 | -0.3728 | -3.0662 | -0.7847 | -0.0902 | 0.067 |
| 164 | -3.0445 | -0.816 | 0.367 | -0.3669 | -3.0635 | -0.7866 | -0.0906 | 0.0672 |
| 165 | -3.0397 | -0.8172 | 0.3604 | -0.3602 | -3.0587 | -0.7878 | -0.0913 | 0.0677 |
| 166 | -3.0318 | -0.8173 | 0.3525 | -0.3523 | -3.0509 | -0.7879 | -0.0922 | 0.0686 |
| 167 | -3.0233 | -0.8173 | 0.3444 | -0.3442 | -3.0424 | -0.7879 | -0.0932 | 0.0694 |
| 168 | -3.0142 | -0.8172 | 0.3362 | -0.3359 | -3.0333 | -0.7878 | -0.0942 | 0.0703 |
| 169 | -3.0028 | -0.8163 | 0.327 | -0.3267 | -3.0219 | -0.7869 | -0.0954 | 0.0714 |
| 170 | -2.9894 | -0.8147 | 0.3171 | -0.3168 | -3.0085 | -0.7854 | -0.0968 | 0.0727 |
| 171 | -2.9776 | -0.8137 | 0.3077 | -0.3074 | -2.9967 | -0.7844 | -0.098 | 0.0739 |
| 172 | -2.9675 | -0.8133 | 0.2989 | -0.2986 | -2.9866 | -0.784 | -0.0991 | 0.0749 |
| 173 | -2.9578 | -0.813 | 0.2903 | -0.2899 | -2.9769 | -0.7837 | -0.1001 | 0.0758 |
| 174 | -2.9494 | -0.8133 | 0.2821 | -0.2817 | -2.9685 | -0.7839 | -0.101 | 0.0766 |
| 175 | -2.9399 | -0.8131 | 0.2735 | -0.273 | -2.959 | -0.7838 | -0.102 | 0.0775 |
| 176 | -2.9292 | -0.8126 | 0.2644 | -0.2639 | -2.9483 | -0.7833 | -0.1031 | 0.0785 |
| 177 | -2.9194 | -0.8124 | 0.2556 | -0.255 | -2.9385 | -0.7831 | -0.1042 | 0.0795 |
| 178 | -2.9099 | -0.8123 | 0.2469 | -0.2463 | -2.9291 | -0.783 | -0.1052 | 0.0804 |
| 179 | -2.8991 | -0.8118 | 0.2376 | -0.237 | -2.9182 | -0.7825 | -0.1063 | 0.0814 |
| 180 | -2.8869 | -0.8109 | 0.2279 | -0.2272 | -2.906 | -0.7816 | -0.1075 | 0.0826 |
| 181 | -2.8739 | -0.8097 | 0.2178 | -0.2172 | -2.893 | -0.7804 | -0.1088 | 0.0838 |
| 182 | -2.8598 | -0.8082 | 0.2074 | -0.2067 | -2.8789 | -0.779 | -0.1101 | 0.0851 |
| 183 | -2.8463 | -0.8069 | 0.1971 | -0.1963 | -2.8654 | -0.7777 | -0.1114 | 0.0864 |
| 184 | -2.8333 | -0.8059 | 0.187 | -0.1862 | -2.8524 | -0.7766 | -0.1127 | 0.0876 |
| 185 | -2.8199 | -0.8047 | 0.1766 | -0.1758 | -2.8389 | -0.7754 | -0.114 | 0.0889 |
| 186 | -2.8063 | -0.8035 | 0.1663 | -0.1654 | -2.8253 | -0.7742 | -0.1152 | 0.0901 |
| 187 | -2.7895 | -0.8011 | 0.1547 | -0.1538 | -2.8086 | -0.772 | -0.1168 | 0.0917 |
| 188 | -2.7692 | -0.7976 | 0.1417 | -0.1408 | -2.7882 | -0.7685 | -0.1188 | 0.0937 |
| 189 | -2.747 | -0.7933 | 0.128 | -0.1271 | -2.766 | -0.7643 | -0.1209 | 0.0959 |
| 190 | -2.7221 | -0.7882 | 0.1133 | -0.1123 | -2.741 | -0.7593 | -0.1233 | 0.0985 |
| 191 | -2.696 | -0.7826 | 0.098 | -0.0971 | -2.7149 | -0.7539 | -0.1258 | 0.1011 |
| 192 | -2.6687 | -0.7766 | 0.0824 | -0.0815 | -2.6875 | -0.7481 | -0.1284 | 0.1039 |
| 193 | -2.6388 | -0.7698 | 0.0657 | -0.0648 | -2.6576 | -0.7414 | -0.1313 | 0.107 |
| 194 | -2.6065 | -0.7621 | 0.0481 | -0.0472 | -2.6252 | -0.7339 | -0.1344 | 0.1103 |
| 195 | -2.5744 | -0.7545 | 0.0306 | -0.0296 | -2.593 | -0.7265 | -0.1375 | 0.1137 |
| 196 | -2.5426 | -0.747 | 0.0131 | -0.0122 | -2.5611 | -0.7192 | -0.1405 | 0.1169 |
| 197 | -2.5098 | -0.7392 | -0.0047 | 0.0057 | -2.5284 | -0.7116 | -0.1437 | 0.1203 |
| 198 | -2.4769 | -0.7314 | -0.0227 | 0.0236 | -2.4953 | -0.704 | -0.1468 | 0.1237 |
| 199 | -2.4418 | -0.7229 | -0.0415 | 0.0424 | -2.4602 | -0.6957 | -0.1502 | 0.1274 |
| 200 | -2.4044 | -0.7135 | -0.0612 | 0.0621 | -2.4226 | -0.6865 | -0.1538 | 0.1313 |
| 201 | -2.3661 | -0.7038 | -0.0812 | 0.0821 | -2.3843 | -0.6771 | -0.1574 | 0.1353 |
| 202 | -2.3265 | -0.6937 | -0.1017 | 0.1026 | -2.3446 | -0.6673 | -0.1612 | 0.1395 |
| 203 | -2.2893 | -0.6847 | -0.1213 | 0.1221 | -2.3073 | -0.6585 | -0.1646 | 0.1431 |
| 204 | -2.2549 | -0.6771 | -0.1396 | 0.1404 | -2.2728 | -0.6511 | -0.1675 | 0.1462 |
| 205 | -2.2214 | -0.6699 | -0.1575 | 0.1584 | -2.2392 | -0.6441 | -0.1702 | 0.1491 |
| 206 | -2.1898 | -0.6637 | -0.1746 | 0.1755 | -2.2076 | -0.638 | -0.1726 | 0.1516 |
| 207 | -2.1584 | -0.6576 | -0.1915 | 0.1924 | -2.1761 | -0.632 | -0.1748 | 0.154 |
| 208 | -2.1271 | -0.6517 | -0.2084 | 0.2094 | -2.1448 | -0.6262 | -0.177 | 0.1563 |
| 209 | -2.0977 | -0.6467 | -0.2245 | 0.2255 | -2.1153 | -0.6214 | -0.1789 | 0.1582 |
| 210 | -2.0701 | -0.6425 | -0.2398 | 0.2409 | -2.0876 | -0.6173 | -0.1804 | 0.1597 |
| 211 | -2.0408 | -0.6379 | -0.2558 | 0.2568 | -2.0583 | -0.6127 | -0.1821 | 0.1614 |
| 212 | -2.0101 | -0.6327 | -0.2723 | 0.2734 | -2.0275 | -0.6076 | -0.1839 | 0.1633 |
| 213 | -1.9793 | -0.6276 | -0.2888 | 0.2899 | -1.9966 | -0.6026 | -0.1856 | 0.1651 |
| 214 | -1.9476 | -0.6223 | -0.3056 | 0.3068 | -1.9649 | -0.5974 | -0.1874 | 0.1669 |
| 215 | -1.9175 | -0.6178 | -0.3218 | 0.323 | -1.9347 | -0.5929 | -0.1889 | 0.1685 |
| 216 | -1.8891 | -0.6141 | -0.3372 | 0.3385 | -1.9062 | -0.5893 | -0.1901 | 0.1696 |
| 217 | -1.8607 | -0.6105 | -0.3525 | 0.3539 | -1.8778 | -0.5857 | -0.1912 | 0.1706 |
| 218 | -1.8328 | -0.6073 | -0.3676 | 0.369 | -1.8499 | -0.5826 | -0.1921 | 0.1715 |
| 219 | -1.8036 | -0.6037 | -0.3833 | 0.3847 | -1.8206 | -0.5789 | -0.1932 | 0.1725 |
| 220 | -1.7726 | -0.5993 | -0.3997 | 0.4012 | -1.7895 | -0.5746 | -0.1945 | 0.1738 |
| 221 | -1.7412 | -0.5949 | -0.4162 | 0.4177 | -1.758 | -0.5703 | -0.1958 | 0.1751 |
| 222 | -1.7088 | -0.5902 | -0.4331 | 0.4347 | -1.7256 | -0.5656 | -0.1972 | 0.1764 |
| 223 | -1.6762 | -0.5855 | -0.4501 | 0.4517 | -1.6929 | -0.561 | -0.1985 | 0.1778 |
| 224 | -1.6434 | -0.5808 | -0.4671 | 0.4688 | -1.66 | -0.5564 | -0.1998 | 0.1791 |
| 225 | -1.6097 | -0.5759 | -0.4845 | 0.4862 | -1.6262 | -0.5515 | -0.2012 | 0.1804 |
| 226 | -1.575 | -0.5707 | -0.5022 | 0.504 | -1.5915 | -0.5463 | -0.2026 | 0.1819 |
| 227 | -1.5417 | -0.5661 | -0.5194 | 0.5213 | -1.558 | -0.5418 | -0.2038 | 0.1831 |
| 228 | -1.5096 | -0.5622 | -0.536 | 0.5379 | -1.5259 | -0.538 | -0.2047 | 0.1839 |
| 229 | -1.478 | -0.5587 | -0.5523 | 0.5544 | -1.4942 | -0.5344 | -0.2055 | 0.1846 |
| 230 | -1.4473 | -0.5557 | -0.5683 | 0.5704 | -1.4635 | -0.5314 | -0.2061 | 0.185 |
| 231 | -1.4161 | -0.5525 | -0.5844 | 0.5866 | -1.4322 | -0.5282 | -0.2067 | 0.1855 |
| 232 | -1.3843 | -0.5492 | -0.6008 | 0.603 | -1.4003 | -0.5249 | -0.2073 | 0.186 |
| 233 | -1.353 | -0.5463 | -0.6169 | 0.6192 | -1.3689 | -0.522 | -0.2077 | 0.1863 |
| 234 | -1.322 | -0.5435 | -0.6329 | 0.6353 | -1.3378 | -0.5192 | -0.2081 | 0.1865 |
| 235 | -1.2878 | -0.5397 | -0.6501 | 0.6526 | -1.3035 | -0.5153 | -0.2088 | 0.1871 |
| 236 | -1.2502 | -0.5345 | -0.6687 | 0.6712 | -1.2659 | -0.5102 | -0.2098 | 0.1881 |
| 237 | -1.2106 | -0.5287 | -0.688 | 0.6907 | -1.2261 | -0.5045 | -0.2111 | 0.1894 |
| 238 | -1.168 | -0.5217 | -0.7086 | 0.7113 | -1.1835 | -0.4976 | -0.2128 | 0.1911 |
| 239 | -1.1247 | -0.5146 | -0.7294 | 0.7321 | -1.1401 | -0.4906 | -0.2144 | 0.1928 |
| 240 | -1.0808 | -0.5074 | -0.7504 | 0.7532 | -1.0961 | -0.4835 | -0.216 | 0.1944 |
| 241 | -1.0343 | -0.4993 | -0.7724 | 0.7753 | -1.0495 | -0.4755 | -0.218 | 0.1965 |
| 242 | -0.9856 | -0.4903 | -0.7953 | 0.7982 | -1.0007 | -0.4667 | -0.2201 | 0.1987 |
| 243 | -0.9368 | -0.4815 | -0.8183 | 0.8212 | -0.9517 | -0.458 | -0.2222 | 0.2009 |
| 244 | -0.8877 | -0.4726 | -0.8413 | 0.8443 | -0.9024 | -0.4493 | -0.2243 | 0.2031 |
| 245 | -0.8376 | -0.4635 | -0.8646 | 0.8677 | -0.8522 | -0.4403 | -0.2264 | 0.2054 |
| 246 | -0.7869 | -0.4543 | -0.8882 | 0.8913 | -0.8014 | -0.4312 | -0.2285 | 0.2076 |
| 247 | -0.734 | -0.4442 | -0.9127 | 0.9158 | -0.7483 | -0.4214 | -0.2309 | 0.2101 |
| 248 | -0.6786 | -0.4333 | -0.9381 | 0.9413 | -0.6928 | -0.4107 | -0.2335 | 0.213 |
| 249 | -0.622 | -0.422 | -0.964 | 0.9672 | -0.636 | -0.3996 | -0.2362 | 0.2159 |
| 250 | -0.5637 | -0.4102 | -0.9906 | 0.9938 | -0.5776 | -0.388 | -0.2391 | 0.219 |
| 251 | -0.5065 | -0.3989 | -1.0166 | 1.0199 | -0.5202 | -0.3769 | -0.2417 | 0.2218 |
| 252 | -0.4508 | -0.3884 | -1.042 | 1.0454 | -0.4643 | -0.3666 | -0.244 | 0.2243 |
| 253 | -0.3949 | -0.378 | -1.0675 | 1.0709 | -0.4083 | -0.3563 | -0.2463 | 0.2268 |
| 254 | -0.3396 | -0.3679 | -1.0926 | 1.0961 | -0.3528 | -0.3464 | -0.2484 | 0.229 |
| 255 | -0.2836 | -0.3577 | -1.118 | 1.1215 | -0.2967 | -0.3364 | -0.2505 | 0.2313 |
| 256 | -0.2268 | -0.3473 | -1.1437 | 1.1472 | -0.2398 | -0.3262 | -0.2527 | 0.2336 |
| 257 | -0.1706 | -0.3373 | -1.1691 | 1.1727 | -0.1834 | -0.3163 | -0.2547 | 0.2357 |
| 258 | -0.1147 | -0.3275 | -1.1944 | 1.198 | -0.1273 | -0.3067 | -0.2566 | 0.2376 |
| 259 | -0.0566 | -0.317 | -1.2204 | 1.2241 | -0.0691 | -0.2964 | -0.2587 | 0.2399 |
| 260 | 0.0036 | -0.3058 | -1.2474 | 1.2511 | -0.0087 | -0.2853 | -0.261 | 0.2423 |
| 261 | 0.065 | -0.2942 | -1.2747 | 1.2785 | 0.0528 | -0.2739 | -0.2633 | 0.2449 |
| 262 | 0.1281 | -0.282 | -1.3027 | 1.3066 | 0.1161 | -0.262 | -0.2659 | 0.2476 |
| 263 | 0.1912 | -0.27 | -1.3307 | 1.3346 | 0.1794 | -0.2502 | -0.2683 | 0.2502 |
| 264 | 0.2541 | -0.2582 | -1.3586 | 1.3626 | 0.2425 | -0.2385 | -0.2706 | 0.2527 |
| 265 | 0.3183 | -0.2461 | -1.3869 | 1.3909 | 0.3069 | -0.2266 | -0.2731 | 0.2553 |
| 266 | 0.3833 | -0.2337 | -1.4155 | 1.4196 | 0.3721 | -0.2144 | -0.2755 | 0.258 |
| 267 | 0.4445 | -0.2218 | -1.4451 | 1.4492 | 0.4334 | -0.2028 | -0.2778 | 0.2604 |
| 268 | 0.5014 | -0.2104 | -1.4758 | 1.48 | 0.4905 | -0.1915 | -0.28 | 0.2628 |
| 269 | 0.5561 | -0.1993 | -1.5071 | 1.5114 | 0.5454 | -0.1806 | -0.2821 | 0.265 |
| 270 | 0.6072 | -0.1887 | -1.5394 | 1.5436 | 0.5966 | -0.1702 | -0.2841 | 0.267 |
| 271 | 0.6577 | -0.1781 | -1.5722 | 1.5765 | 0.6473 | -0.1597 | -0.2861 | 0.2691 |
| 272 | 0.7077 | -0.1673 | -1.6057 | 1.61 | 0.6974 | -0.1491 | -0.2881 | 0.2711 |
| 273 | 0.7545 | -0.157 | -1.6401 | 1.6444 | 0.7443 | -0.1389 | -0.29 | 0.2731 |
| 274 | 0.7986 | -0.1469 | -1.6753 | 1.6797 | 0.7886 | -0.1289 | -0.2918 | 0.2749 |
| 275 | 0.8429 | -0.1368 | -1.7108 | 1.7152 | 0.833 | -0.119 | -0.2936 | 0.2767 |
| 276 | 0.8872 | -0.1267 | -1.7465 | 1.751 | 0.8774 | -0.109 | -0.2954 | 0.2785 |
| 277 | 0.9305 | -0.1167 | -1.7827 | 1.7872 | 0.9208 | -0.0992 | -0.2971 | 0.2802 |
| 278 | 0.9733 | -0.1067 | -1.8193 | 1.8238 | 0.9637 | -0.0894 | -0.2988 | 0.2818 |
| 279 | 1.0132 | -0.0971 | -1.8568 | 1.8613 | 1.0038 | -0.0799 | -0.3003 | 0.2834 |
| 280 | 1.05 | -0.0879 | -1.8951 | 1.8996 | 1.0407 | -0.0708 | -0.3018 | 0.2848 |
| 281 | 1.0854 | -0.0788 | -1.934 | 1.9385 | 1.0761 | -0.0618 | -0.3032 | 0.2861 |
| 282 | 1.1187 | -0.07 | -1.9735 | 1.9781 | 1.1096 | -0.0531 | -0.3046 | 0.2873 |
| 283 | 1.1537 | -0.0608 | -2.0135 | 2.0181 | 1.1447 | -0.044 | -0.306 | 0.2887 |
| 284 | 1.1909 | -0.0511 | -2.0538 | 2.0584 | 1.1819 | -0.0344 | -0.3075 | 0.2901 |
| 285 | 1.228 | -0.0412 | -2.0946 | 2.0992 | 1.2192 | -0.0247 | -0.3091 | 0.2916 |
| 286 | 1.2662 | -0.031 | -2.136 | 2.1406 | 1.2574 | -0.0146 | -0.3107 | 0.2932 |
| 287 | 1.3035 | -0.0209 | -2.1778 | 2.1825 | 1.2948 | -0.0046 | -0.3123 | 0.2947 |
| 288 | 1.3398 | -0.0108 | -2.2203 | 2.225 | 1.3312 | 0.0053 | -0.3139 | 0.2962 |
| 289 | 1.377 | -0.0005 | -2.2632 | 2.2679 | 1.3685 | 0.0155 | -0.3155 | 0.2978 |
| 290 | 1.4147 | 0.0101 | -2.3067 | 2.3113 | 1.4063 | 0.0259 | -0.3172 | 0.2994 |
| 291 | 1.4494 | 0.0203 | -2.3509 | 2.3556 | 1.4412 | 0.036 | -0.3187 | 0.3008 |
| 292 | 1.4813 | 0.0301 | -2.396 | 2.4007 | 1.4731 | 0.0457 | -0.3202 | 0.3022 |
| 293 | 1.5115 | 0.0398 | -2.4418 | 2.4465 | 1.5035 | 0.0553 | -0.3216 | 0.3034 |
| 294 | 1.5394 | 0.0493 | -2.4884 | 2.493 | 1.5314 | 0.0646 | -0.3229 | 0.3046 |
| 295 | 1.5672 | 0.0589 | -2.5355 | 2.5402 | 1.5593 | 0.0741 | -0.3243 | 0.3058 |
| 296 | 1.5953 | 0.0686 | -2.5832 | 2.5879 | 1.5874 | 0.0837 | -0.3257 | 0.307 |
| 297 | 1.6215 | 0.0782 | -2.6317 | 2.6363 | 1.6138 | 0.0932 | -0.327 | 0.3082 |
| 298 | 1.6466 | 0.0877 | -2.6809 | 2.6855 | 1.6389 | 0.1026 | -0.3283 | 0.3093 |
| 299 | 1.6732 | 0.0974 | -2.7299 | 2.7346 | 1.6656 | 0.1122 | -0.3296 | 0.3104 |
| 300 | 1.7014 | 0.1073 | -2.7789 | 2.7835 | 1.6939 | 0.1219 | -0.3308 | 0.3115 |
| 301 | 1.7303 | 0.1172 | -2.828 | 2.8326 | 1.7229 | 0.1318 | -0.3321 | 0.3126 |
| 302 | 1.7607 | 0.1273 | -2.8771 | 2.8817 | 1.7533 | 0.1417 | -0.3334 | 0.3137 |
| 303 | 1.7904 | 0.1374 | -2.9267 | 2.9313 | 1.7831 | 0.1517 | -0.3346 | 0.3148 |
| 304 | 1.8193 | 0.1474 | -2.9769 | 2.9815 | 1.8122 | 0.1616 | -0.3358 | 0.3158 |
| 305 | 1.8493 | 0.1575 | -3.027 | 3.0316 | 1.8422 | 0.1716 | -0.3369 | 0.3167 |
| 306 | 1.8798 | 0.1677 | -3.0774 | 3.082 | 1.8728 | 0.1817 | -0.3381 | 0.3177 |
| 307 | 1.9096 | 0.1779 | -3.1284 | 3.133 | 1.9027 | 0.1917 | -0.3392 | 0.3186 |
| 308 | 1.9388 | 0.1881 | -3.18 | 3.1846 | 1.932 | 0.2019 | -0.3403 | 0.3195 |
| 309 | 1.9677 | 0.1983 | -3.2321 | 3.2367 | 1.961 | 0.212 | -0.3415 | 0.3204 |
| 310 | 1.996 | 0.2086 | -3.2848 | 3.2894 | 1.9894 | 0.2222 | -0.3425 | 0.3213 |
| 311 | 2.0251 | 0.219 | -3.3376 | 3.3422 | 2.0186 | 0.2324 | -0.3436 | 0.3222 |
| 312 | 2.055 | 0.2294 | -3.3906 | 3.3951 | 2.0485 | 0.2427 | -0.3447 | 0.323 |
| 313 | 2.0848 | 0.2399 | -3.4439 | 3.4485 | 2.0785 | 0.2531 | -0.3457 | 0.3238 |
| 314 | 2.1149 | 0.2505 | -3.4976 | 3.5022 | 2.1087 | 0.2636 | -0.3467 | 0.3246 |
| 315 | 2.1426 | 0.2608 | -3.5523 | 3.5568 | 2.1365 | 0.2738 | -0.3477 | 0.3254 |
| 316 | 2.1676 | 0.2709 | -3.6079 | 3.6124 | 2.1615 | 0.2839 | -0.3486 | 0.326 |
| 317 | 2.1914 | 0.281 | -3.6642 | 3.6687 | 2.1854 | 0.2938 | -0.3495 | 0.3266 |
| 318 | 2.2131 | 0.2908 | -3.7213 | 3.7258 | 2.2072 | 0.3035 | -0.3503 | 0.3271 |
| 319 | 2.2342 | 0.3006 | -3.7789 | 3.7834 | 2.2283 | 0.3133 | -0.351 | 0.3275 |
| 320 | 2.2547 | 0.3104 | -3.8371 | 3.8416 | 2.2488 | 0.323 | -0.3518 | 0.328 |
| 321 | 2.2732 | 0.32 | -3.8961 | 3.9006 | 2.2674 | 0.3325 | -0.3524 | 0.3283 |
| 322 | 2.2901 | 0.3295 | -3.9559 | 3.9603 | 2.2843 | 0.342 | -0.3531 | 0.3286 |
| 323 | 2.3074 | 0.3391 | -4.0159 | 4.0203 | 2.3018 | 0.3515 | -0.3537 | 0.3289 |
| 324 | 2.3253 | 0.3488 | -4.0762 | 4.0806 | 2.3197 | 0.361 | -0.3543 | 0.3291 |
| 325 | 2.3428 | 0.3584 | -4.1369 | 4.1413 | 2.3373 | 0.3706 | -0.3549 | 0.3293 |
| 326 | 2.3605 | 0.3681 | -4.198 | 4.2023 | 2.355 | 0.3802 | -0.3554 | 0.3295 |
| 327 | 2.3766 | 0.3777 | -4.2598 | 4.2641 | 2.3712 | 0.3897 | -0.3559 | 0.3296 |
| 328 | 2.3911 | 0.3871 | -4.3223 | 4.3266 | 2.3857 | 0.3991 | -0.3563 | 0.3297 |
| 329 | 2.4052 | 0.3965 | -4.3853 | 4.3896 | 2.3999 | 0.4084 | -0.3567 | 0.3297 |
| 330 | 2.4185 | 0.4059 | -4.4489 | 4.4531 | 2.4133 | 0.4178 | -0.3571 | 0.3296 |
| 331 | 2.4351 | 0.4159 | -4.5128 | 4.5169 | 2.4299 | 0.4278 | -0.3576 | 0.3298 |
| 332 | 2.4554 | 0.4268 | -4.577 | 4.5811 | 2.4503 | 0.4386 | -0.3584 | 0.3302 |
| 333 | 2.4774 | 0.4382 | -4.6417 | 4.6458 | 2.4723 | 0.4498 | -0.3592 | 0.3307 |
| 334 | 2.5021 | 0.4501 | -4.7068 | 4.7108 | 2.4971 | 0.4616 | -0.3602 | 0.3313 |
| 335 | 2.5276 | 0.4624 | -4.7723 | 4.7763 | 2.5227 | 0.4737 | -0.3612 | 0.3321 |
| 336 | 2.5539 | 0.4748 | -4.8383 | 4.8423 | 2.5491 | 0.486 | -0.3622 | 0.3328 |
| 337 | 2.5829 | 0.4879 | -4.9046 | 4.9086 | 2.5782 | 0.4989 | -0.3634 | 0.3337 |
| 338 | 2.6144 | 0.5015 | -4.9714 | 4.9753 | 2.6098 | 0.5124 | -0.3647 | 0.3348 |
| 339 | 2.645 | 0.5151 | -5.0388 | 5.0427 | 2.6405 | 0.5258 | -0.366 | 0.3359 |
| 340 | 2.6747 | 0.5286 | -5.1068 | 5.1106 | 2.6703 | 0.5392 | -0.3672 | 0.3368 |
| 341 | 2.705 | 0.5424 | -5.1753 | 5.1791 | 2.7007 | 0.5528 | -0.3685 | 0.3379 |
| 342 | 2.7351 | 0.5562 | -5.2444 | 5.2482 | 2.7309 | 0.5665 | -0.3698 | 0.3389 |
| 343 | 2.7676 | 0.5706 | -5.3139 | 5.3177 | 2.7635 | 0.5808 | -0.3712 | 0.3401 |
| 344 | 2.8026 | 0.5857 | -5.3839 | 5.3876 | 2.7987 | 0.5956 | -0.3727 | 0.3414 |
| 345 | 2.8384 | 0.6009 | -5.4543 | 5.458 | 2.8346 | 0.6107 | -0.3743 | 0.3428 |
| 346 | 2.8755 | 0.6166 | -5.5253 | 5.5289 | 2.8718 | 0.6261 | -0.376 | 0.3443 |
| 347 | 2.9117 | 0.6321 | -5.5968 | 5.6003 | 2.9081 | 0.6414 | -0.3776 | 0.3457 |
| 348 | 2.9465 | 0.6475 | -5.6688 | 5.6723 | 2.943 | 0.6566 | -0.3791 | 0.347 |
| 349 | 2.9815 | 0.6629 | -5.7413 | 5.7448 | 2.9782 | 0.6719 | -0.3806 | 0.3483 |
| 350 | 3.0161 | 0.6784 | -5.8144 | 5.8178 | 3.0129 | 0.6872 | -0.3821 | 0.3495 |
| 351 | 3.0511 | 0.6941 | -5.8879 | 5.8913 | 3.0481 | 0.7026 | -0.3836 | 0.3508 |
| 352 | 3.0867 | 0.71 | -5.962 | 5.9654 | 3.0838 | 0.7183 | -0.3852 | 0.3522 |
| 353 | 3.1219 | 0.7258 | -6.0366 | 6.0399 | 3.119 | 0.734 | -0.3867 | 0.3535 |
| 354 | 3.1567 | 0.7417 | -6.1118 | 6.115 | 3.154 | 0.7497 | -0.3882 | 0.3547 |
| 355 | 3.1936 | 0.7582 | -6.1874 | 6.1906 | 3.191 | 0.7659 | -0.3898 | 0.3561 |
| 356 | 3.2327 | 0.7751 | -6.2635 | 6.2666 | 3.2303 | 0.7827 | -0.3915 | 0.3577 |
| 357 | 3.273 | 0.7924 | -6.3401 | 6.3431 | 3.2707 | 0.7997 | -0.3933 | 0.3593 |
| 358 | 3.315 | 0.8102 | -6.4171 | 6.4201 | 3.3129 | 0.8172 | -0.3952 | 0.361 |
| 359 | 3.3572 | 0.828 | -6.4947 | 6.4976 | 3.3552 | 0.8348 | -0.3971 | 0.3627 |
| 360 | 3.3993 | 0.846 | -6.5728 | 6.5757 | 3.3974 | 0.8525 | -0.3989 | 0.3644 |
| 361 | 3.4427 | 0.8643 | -6.6514 | 6.6542 | 3.441 | 0.8706 | -0.4009 | 0.3662 |
| 362 | 3.4871 | 0.8829 | -6.7305 | 6.7332 | 3.4855 | 0.8889 | -0.4029 | 0.368 |
| 363 | 3.5289 | 0.9011 | -6.8104 | 6.8131 | 3.5275 | 0.9069 | -0.4048 | 0.3698 |
| 364 | 3.5679 | 0.919 | -6.8911 | 6.8937 | 3.5667 | 0.9246 | -0.4065 | 0.3713 |
| 365 | 3.6056 | 0.9368 | -6.9725 | 6.9751 | 3.6045 | 0.9422 | -0.4082 | 0.3728 |
| 366 | 3.6409 | 0.9543 | -7.0547 | 7.0573 | 3.64 | 0.9594 | -0.4098 | 0.3742 |
| 367 | 3.6763 | 0.9719 | -7.1376 | 7.14 | 3.6754 | 0.9768 | -0.4115 | 0.3755 |
| 368 | 3.7117 | 0.9897 | -7.221 | 7.2234 | 3.711 | 0.9944 | -0.4131 | 0.3769 |
| 369 | 3.7452 | 1.0073 | -7.3052 | 7.3075 | 3.7446 | 1.0117 | -0.4146 | 0.3782 |
| 370 | 3.7772 | 1.0247 | -7.3901 | 7.3923 | 3.7767 | 1.0289 | -0.4161 | 0.3794 |
| 371 | 3.8097 | 1.0423 | -7.4756 | 7.4777 | 3.8093 | 1.0463 | -0.4176 | 0.3806 |
| 372 | 3.8426 | 1.0601 | -7.5616 | 7.5637 | 3.8424 | 1.0639 | -0.4191 | 0.3819 |
| 373 | 3.8752 | 1.078 | -7.6482 | 7.6502 | 3.8751 | 1.0815 | -0.4207 | 0.3831 |
| 374 | 3.908 | 1.096 | -7.7354 | 7.7373 | 3.9081 | 1.0993 | -0.4222 | 0.3843 |
| 375 | 3.9392 | 1.1139 | -7.8234 | 7.8252 | 3.9394 | 1.117 | -0.4237 | 0.3855 |
| 376 | 3.9686 | 1.1315 | -7.9121 | 7.9138 | 3.9689 | 1.1344 | -0.4251 | 0.3866 |
| 377 | 3.9974 | 1.1492 | -8.0014 | 8.0031 | 3.9978 | 1.1519 | -0.4264 | 0.3876 |
| 378 | 4.0253 | 1.1669 | -8.0915 | 8.0931 | 4.0258 | 1.1694 | -0.4278 | 0.3886 |
| 379 | 4.0551 | 1.185 | -8.182 | 8.1835 | 4.0557 | 1.1873 | -0.4292 | 0.3897 |
| 380 | 4.0872 | 1.2037 | -8.273 | 8.2744 | 4.0879 | 1.2057 | -0.4307 | 0.391 |
| 381 | 4.1198 | 1.2226 | -8.3645 | 8.3659 | 4.1207 | 1.2244 | -0.4323 | 0.3922 |
| 382 | 4.154 | 1.2419 | -8.4566 | 8.4578 | 4.155 | 1.2434 | -0.434 | 0.3936 |
| 383 | 4.1881 | 1.2614 | -8.5493 | 8.5504 | 4.1893 | 1.2626 | -0.4356 | 0.395 |
| 384 | 4.2222 | 1.2809 | -8.6426 | 8.6436 | 4.2235 | 1.2819 | -0.4373 | 0.3964 |
| 385 | 4.2577 | 1.3009 | -8.7364 | 8.7374 | 4.2591 | 1.3016 | -0.439 | 0.3978 |
| 386 | 4.2943 | 1.3211 | -8.8308 | 8.8317 | 4.2959 | 1.3216 | -0.4408 | 0.3993 |
| 387 | 4.3294 | 1.3413 | -8.9259 | 8.9267 | 4.3311 | 1.3415 | -0.4425 | 0.4008 |
| 388 | 4.3631 | 1.3613 | -9.0218 | 9.0225 | 4.3649 | 1.3612 | -0.4442 | 0.4022 |
| 389 | 4.3963 | 1.3814 | -9.1184 | 9.1189 | 4.3983 | 1.3811 | -0.4459 | 0.4035 |
| 390 | 4.4286 | 1.4014 | -9.2156 | 9.2161 | 4.4307 | 1.4008 | -0.4475 | 0.4048 |
| 391 | 4.4616 | 1.4217 | -9.3135 | 9.3138 | 4.4639 | 1.4209 | -0.4492 | 0.4062 |
| 392 | 4.4956 | 1.4423 | -9.4119 | 9.4122 | 4.4981 | 1.4412 | -0.4509 | 0.4076 |
| 393 | 4.5292 | 1.463 | -9.511 | 9.5112 | 4.5317 | 1.4616 | -0.4526 | 0.409 |
| 394 | 4.5627 | 1.4838 | -9.6108 | 9.6108 | 4.5654 | 1.4822 | -0.4543 | 0.4104 |
| 395 | 4.5913 | 1.5023 | -9.7013 | 9.7012 | 4.5941 | 1.5004 | -0.4556 | 0.4114 |
| 396 | 4.6145 | 1.5181 | -9.7816 | 9.7814 | 4.6174 | 1.516 | -0.4564 | 0.412 |
| 397 | 4.6347 | 1.5326 | -9.8562 | 9.856 | 4.6377 | 1.5301 | -0.457 | 0.4123 |
| 398 | 4.6505 | 1.5449 | -9.9224 | 9.9221 | 4.6536 | 1.5422 | -0.4571 | 0.4122 |
| 399 | 4.6643 | 1.5565 | -9.9857 | 9.9853 | 4.6675 | 1.5534 | -0.4572 | 0.412 |
| 400 | 4.6762 | 1.5673 | -10.0464 | 10.0459 | 4.6796 | 1.564 | -0.457 | 0.4116 |
